# MITEs in the promoters of effector genes allow prediction of novel virulence genes in *Fusarium oxysporum*

**DOI:** 10.1186/1471-2164-14-119

**Published:** 2013-02-22

**Authors:** Sarah M Schmidt, Petra M Houterman, Ines Schreiver, Lisong Ma, Stefan Amyotte, Biju Chellappan, Sjef Boeren, Frank L W Takken, Martijn Rep

**Affiliations:** 1 Plant Pathology, Swammerdam Institute for Life Sciences, University of Amsterdam, Science Park 904, 1098 XH, Amsterdam, the Netherlands; 2 Department of Plant Pathology, University of Kentucky, 201F Plant Science Building, 1405 Veterans Drive, 40546-0312, Lexington, KY, USA; 3 Laboratory for Biochemistry, Wageningen University, Dreijenlaan 3, 6703HA, Wageningen, the Netherlands; 4 Current address: Fachgebiet Medizinische Biotechnologie, Institut für Biotechnologie, Technische Universität Berlin, Gustav-Meyer-Allee 25, Berlin, Germany

## Abstract

**Background:**

The plant-pathogenic fungus *Fusarium oxysporum* f.sp*.lycopersici* (*Fol*) has accessory, lineage-specific (LS) chromosomes that can be transferred horizontally between strains. A single LS chromosome in the *Fol4287* reference strain harbors all known *Fol* effector genes. Transfer of this pathogenicity chromosome confers virulence to a previously non-pathogenic recipient strain. We hypothesize that expression and evolution of effector genes is influenced by their genomic context.

**Results:**

To gain a better understanding of the genomic context of the effector genes, we manually curated the annotated genes on the pathogenicity chromosome and identified and classified transposable elements. Both retro- and DNA transposons are present with no particular overrepresented class. Retrotransposons appear evenly distributed over the chromosome, while DNA transposons tend to concentrate in large chromosomal subregions. In general, genes on the pathogenicity chromosome are dispersed within the repeat landscape. Effector genes are present within subregions enriched for DNA transposons. A miniature Impala (mimp) is always present in their promoters. Although promoter deletion studies of two effector gene loci did not reveal a direct function of the mimp for gene expression, we were able to use proximity to a mimp as a criterion to identify new effector gene candidates. Through xylem sap proteomics we confirmed that several of these candidates encode proteins secreted during plant infection.

**Conclusions:**

Effector genes in *Fol* reside in characteristic subregions on a pathogenicity chromosome. Their genomic context allowed us to develop a method for the successful identification of novel effector genes. Since our approach is not based on effector gene similarity, but on unique genomic features, it can easily be extended to identify effector genes in *Fo* strains with different host specificities.

## Background

The tomato pathogenic fungus *Fusarium oxysporum* forma specialis *lycopersisci* (*Fol*) posses a two-partite genome. Eleven of the 15 chromosomes of the sequenced strain (*Fol4287*) are syntenic with chromosomes of the sister species *Fusarium verticilloides* and the more distantly related *Fusarium graminearum*, displaying high sequence similarity and conservation of gene order [1]. These core chromosomes contain all housekeeping genes and few transposable elements (TEs). Additionally, *Fol4287* possesses four chromosomes that are devoid of housekeeping genes and accommodate 74% of the whole genome TE content and 95% of the class II TEs (DNA transposons). The four chromosomes and two smaller regions at the ends of two core chromosomes comprise the lineage-specific (LS) part of the *Fol* genome. The genes encoded in LS regions differ in their phylogenetic history from the genes on the core chromosomes [1,2]. The term lineage-specific (LS) reflects the largely clonal structure of the *Fo* species complex. *Fo* reproduces asexually and consists of many clonal lineages, which, if pathogenic, are grouped into host-specific *formae speciales* (ff. spp.) [3]. While some ff. spp. are monophyletic, others are composed of several clonal lineages that appear to have independently acquired the ability to infect the same host plant [4-6]. This polyphyletic origin was likely caused by horizontal transfer of chromosomes encoding host specific virulence genes between *Fo* lineages, thereby allowing the distinction of members of a f. sp., not by overall genetic relatedness, but by the presence or absence of certain LS chromosomes [1].

In *Fol*, one LS chromosome (chromosome 14 of *Fol4287*) largely defines the pathogenic phenotype of this f. sp., i.e. the ability to cause wilt disease in tomato. Horizontal transfer of this pathogenicity chromosome from a tomato pathogenic isolate to a non-pathogenic isolate during co-cultivation resulted in novel tomato-pathogenic lineages, demonstrating that it contains genes that promote infection of tomato [1]. Among these genes are all known *Fol4287* effector genes called *SIX* (Secreted In Xylem) genes. Like many other plant pathogens, *Fol* utilizes small, secreted proteins to promote virulence by manipulating its plant host and suppressing host defense responses, typically through interaction with host proteins [7,8]. Six proteins are small, commonly cysteine-rich, lack homology to other proteins and have a signal peptide for secretion [8]. Six of the seven previously described Six proteins are encoded on the pathogenicity chromosome; the genomic location of *SIX4*, whose gene product is recognized by the tomato resistance proteins I and I-1, is unknown because it is not present in the sequenced race 2 isolate *Fol4287*. Although *SIX* genes were likely acquired by horizontal transfer of the pathogenicity chromosome, they are not functionally independent of the core genome. Their expression requires the transcription factor Sge1 (*SIX* gene expression 1), which is encoded on a core chromosome [9]. It is unknown whether Sge1 regulates *SIX* gene expression directly or indirectly, for example through the action of other transcription factors.

Effector genes in other plant pathogens, such as *Magnoporthe oryzae*, *Leptosphaeria maculans* or *Phythophthora infestans*, are also found proximal to TEs and TEs have been proposed as the underlying agents that provide a plastic environment for the emergence of new virulence traits [10-12]. The potential of TEs to affect genome structure is a consequence of both their mobility and their inherent structure. Generally, two different TE classes are distinguished by their transposition intermediate: RNA or DNA. Class I TEs (or retrotransposons) transpose via a “copy-paste mechanism” by copying themselves into an RNA-intermediate before inserting at a new site, while class II TEs (or DNA transposons) leave the donor site to reintegrate at another site via a “cut-paste mechanism”, although the original copy can also be retained [13]. Class I TEs are either flanked by terminal inverted repeats (TIRs), long terminal repeats (LTRs) or simple non-coding regions. Class II TEs are usually flanked by TIRs [14]. Special TE families are the MITEs (Miniature Inverted-repeat Tranposable Elements), non-autonomous class II TEs of short length, which are thought to have evolved from autonomous TEs by deletion of their transposase ORF [15]. Recombination between identical or highly similar TEs can cause structural rearrangements like deletions, inversions, duplications and translocations depending on the orientation and genomic location of the recombining TE members [16]. For an asexual fungus like *Fol,* TE-mediated recombination might represent a mechanism to create genetic variation in the absence of meiotic recombination. Next to gross structural rearrangements, TEs also contribute to evolution of novel phenotypes by transposition into new sites. For example, insertion of the hAT transposase Drifter into the coding sequence of an ancestral *SIX1* homolog (*SIX1-H*) disrupted the open-reading frame (ORFs) of *SIX1-H*, thus creating an effector pseudogene [8]. In another case, insertion of a Hornet-like transposon at the *SIX4* locus of a japanese race 3 *Fol* isolate created a fusion protein, which was no longer recognized by the corresponding I-1 tomato resistance protein [17]. TE insertion might also influence gene expression when it occurs within a promoter.

To further our understanding of the molecular basis of pathogenicity of *Fol* towards tomato, we conducted a detailed annotation of the predicted proteins encoded by the *Fol* pathogenicity chromosome. In addition, to advance our understanding of the potential role of the genomic context of effector genes in gene evolution or expression, we also annotated TEs on this chromosome. We thus obtained a detailed picture of the genomic landscape of the pathogenicity chromosome. Within this TE-rich landscape, we recognized mini-clusters of *SIX* genes. *SIX* genes are associated with two MITEs: a mimp upstream in all cases and, frequently, an mFot5 downstream. Using promoter deletions at two *SIX* gene loci, we studied the influence of the mimp on *SIX* gene expression. Finally, we were able to exploit the consistent presence of a mimp in the promoters of *SIX* genes and other virulence-associated genes to develop a method to identify candidate effector genes in *F. oxysporum*.

## Results

### Non-TE genes on the *Fol* pathogenicity chromosome group into a small set of functional classes

**Figure 1:**
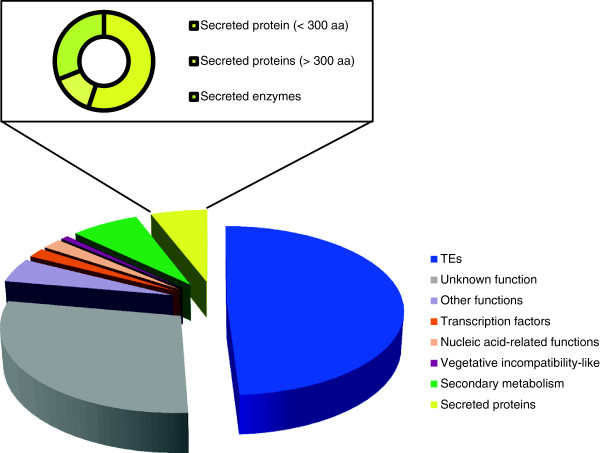
**TEs dominate on the pathogenicity chromosome.** TEs and non-TE genes are presented as percentage of the total TE/gene content. Genes coding for secreted proteins (including *SIX* genes) constitute one of the best-represented classes.

Non-TE ORFs occupy only 13% of the DNA space on the pathogenicity chromosome of *Fol*, which consists of four supercontigs (sc) (sc 22, 36, 43, 51) in the most recent *Fol* genome assembly (Li-Jun Ma, personal communication, Table 1). Most of the manually curated 245 non-TE ORFs on this chromosome encode proteins of unknown function, which are annotated as hypothetical proteins or proteins with domains of unknown function (140 ORFs, Figure 1). Some of these unknown proteins have homologous sequences in *F. oxysporum* or in other fungi (Additional file 1). Two functional groups stand out among the predicted products of the remaining 103 non-TE ORFs: secreted proteins (29) and proteins involved in secondary metabolism (35). Other functional groups include transcription factors (11), proteins with nucleic acid related functions (10), heterokaryon incompatibility (Het) proteins (4), transporters (3), cyclins (3) and other intracellular functions (17), such as GTPases and protein kinases (Figure 1). As reported by Ma *et al*., there are no genes for housekeeping proteins on the pathogenicity chromosome [1]. Among the predicted secreted proteins, we find nine secreted enzymes, such as oxidoreductase, chitinase and glucanase, and 20 secreted proteins of unknown function. Sixteen of the latter encode proteins smaller than 300 amino acids. Among those are the previously described effector genes *SIX1*, *SIX2*, *SIX3*, *SIX5*, *SIX6* and *SIX7*[18-20]. Proteins encoded on the *Fol* pathogenicity chromosome that are likely involved in secondary metabolism [1] include methyl transferases (6), cytochrome P450s (6) and glycosyltransferases (3). A putative secondary metabolite gene cluster on sc51 includes genes for three cytochrome P450s, a glycosyltransferase, a methyltransferase, a squalene-hopene cyclase and a homolog of Tri7, an acetyltransferase that is part of the trichothecene gene cluster in *Gibberella zeae*[21]. The genes in this putative secondary metabolite cluster are expressed during tomato infection (Additional file 2) and might therefore be important for pathogenicity of the fungus.

**Table 1:** Space occupied by TEs and non-TE ORFs on the pathogenicity chromosome

	**In bp**	**Percent of sequence**
non-TE ORFs	324923	13
TEs	581563	24
total	2457923	100

Currently, it is not known how *F. oxysporum* can transfer chromosomes horizontally from one strain to another. One hypothesis is that horizontal chromosome transfer (HCT) occurs via anastomosis tubes – specialized, unbranched tubes that connect conidia or hyphae [2,22]. Anastomosis tubes result in heterokaryon formation between two fungal individuals [23]. This heterokaryon is only viable if the individuals have the same *HET* (Heterokaryon incompatibility) genotype; otherwise it undergoes a characteristic cell death reaction called an incompatibility reaction [24]. Four genes on the pathogenicity chromosome encode proteins with similarity to Het proteins in other fungi (FOXG_14188, FOXG_14292, FOXG_14283, FOXG_14284). Het proteins like Het-E from *Podospora anserina* often harbor NACHT (NAIP, CIIA, HET-E, TP1) domains, or a highly divergent nucleoside phosphorylase (Pfs) linked with protein-binding modules such as Ankyrin repeats [25]. FOXG_14188 encodes a protein with a NACHT domain, FOXG_14292 a protein with a Pfs domain and Ankyrin repeats, FOXG_14283 a protein with a Pfs, an ATPase and Ankyrin repeats and FOXG_14284 a protein with Pfs and Ankyrin repeats (Additional file 1). The presence of *HET*-like genes on the pathogenicity chromosome may be seen to contradict HCT via anastomosis tubes, because additional *HET* loci would raise the chance of incompatibility between strains, involving programmed cell death of fused compartments. On the other hand, incompatibility does not appear to be a barrier to HCT [1]. Moreover, we do not know whether the *HET*-like genes on the pathogenicity chromosome are really involved in incompatibility.

Since transfer of the pathogenicity chromosome is sufficient to confer pathogenicity towards tomato, the virulence genes on it must be expressed in the new host strain. We know that there is crosstalk between the core and pathogenicity chromosome, because the core chromosome-encoded Sge1 controls *SIX* gene expression [26]. The presence of eleven genes encoding transcription factors on the pathogenicity chromosome suggests that transcription of genes on the pathogenicity chromosome may also be controlled by the chromosome itself. Among the transcription factors encoded on the pathogenicity chromosome are three copies of *FTF1,* which is induced upon plant infection [27], suggesting that at least a subset of the transcription factors encoded on the pathogenicity chromosome may be required for transcriptional reprogramming during plant infection.

Next to transcription factors, nine other genes encode proteins with nucleic acid-related functions (Figure 1). Most of these proteins are predicted to function in structural rearrangements of DNA or in chromatin modifications. FOXG_16427 encodes a poly(ADP)-ribose polymerase (Parp1) which binds to damaged or single-stranded DNA to recruit DNA repairing enzymes [28]. Other genes encode putative components of the RNA silencing machinery, including closely spaced genes for an RNA-dependent RNA polymerase (FOXG_16453), an RNA interference and gene silencing protein (FOXG_16455) and a RNaseH domain-containing protein (FOXG_16456). FOXG_14161 encodes a protein homologous to the eukaryotic conserved kinetochore protein Mis12 that is involved correct segregation of daughter chromatids during mitosis and meiosis [29]. FOXG_14165 encodes a protein with a BAH (bromo-adjacent homology) domain which may interact with gene silencing components [30]. Similarly, FOXG_14186 encodes a chromodomain protein that typically recruits protein complexes to chromatin and reads the epigenetic code by recognizing lysine methylation [31]. Proteins involved in chromatin modification and RNA interference might influence gene expression during pathogenicity.

### The *Fol* pathogenicity chromosome harbours a large diversity of transposable elements

To exhaustively identify TEs and TE relics on the *Fol* pathogenicity chromosome, we performed a self-BLASTN of the genome sequence, then identified multi-copy sequences and sorted them into non-redundant families. Secondly, we looked for inverted repeats (IRs) of at least 19 bp encompassing at most 5 kb of sequence. This expanded the set of identified TEs relative to an initial survey [1]. Taken together, TEs occupy about twice as much (24%) chromosomal DNA space as non-TE ORFs (13%, Table 1).

Both Class I and Class II TEs (full length and fragments) are present in approximately equal numbers (266 Class I, 249 class II, Table 2) on the pathogenicity chromosome, which is surprising because retrotransposons often dominate the TE fraction of a given genome [11,32-34]. For annotation of the TE classes we followed the classification system proposed by Wicker and colleagues that comprises both mechanistic and enzymatic criteria [14]. Class I TEs all transpose by transcribing themselves into an RNA intermediate, then reverse-transcribing the RNA by a TE-encoded reverse transcriptase and inserting into a new genomic region. There are three orders of class I TEs: Long-terminal-repeat (LTR) TEs, long-interspersed nuclear elements (LINE) and short interspersed nuclear elements (SINE).

**Table 2:** **Transposable elements on the *Fol4287 *pathogenicity chromosome**

**Classification**				**Designation**^1^	**Number**	**Full length**
**Order**	**Superfamily**	**Family**	**Number**			**number**
**Class I (retrotransposon)**			**237**		**237**	**58**
LTR	Gypsy/Ty3		24	MAGGY-like retrotransposon (3 types)	16	4
				**Skippy**	8	1
	Copia/Ty1		55	NHT2-like retrotransposon (5 types)	51	1
				Pcretro3-like retrotransposon	4	1
	unclassified		20	Yaret2	20	6
	solo-LTR		28	Yaret2 solo-LTR	12	12
				Gollum (NHT2-like retrotransposon type 3 LTR)	16	10
LINE			68	MGR583-like LINE element	31	3
				Yaret1	25	3
				Yaret1-like	12	0
SINE			32	**Foxy**	32	10
unrelated			10	**Marsu**	10	7
**Class II (DNA transposons) - Subclass 1**			**208**		**208**	**108**
Crypton			1	FoCrypton	1	1
TIR	Tc1/mariner	Pogo	41	**Fot2**	2	2
				**Fot3**	7	2
				**Fot4**	1	0
				**Fot5**	23	6
				Fot6	6	3
				Fot8	2	0
		Tc1	3	**Impala**	3	0
		hAT	70	**Folyt1**	3	3
				Folyt2	1	0
				Frodo	5	3
				**Hornet**	16	7
				**Drifter**	1	1
				NhORF4-like	2	1
				Sam	1	1
				YahAT1	6	5
				YahAT2	9	4
				YahAT3	3	1
				YahAT4	2	1
				YahAT5	4	2
				YahAT6	7	3
				YahAT7	10	1
		Mutator	20	**Hop**	1	0
				Hop3	6	4
				Hop4	5	0
				Hop5	2	1
				Hop6	6	0
		MITE	73	**mimp (unclassified)**	2	2
				**mimp1**	24	16
				**mimp2**	7	6
				**mimp3**	6	5
				**mimp4**	17	16
				**mFot5**	14	11
				Gimli	3	0
**Class II (DNA transposons) - Subclass 2**			**9**			
				Helitron	9	8
**Class II (DNA transposons) - unclassified**			**40**			
				unclassified	40	2
**total number of TEs**			**494**		**494**	**176**

^1^ designations in bold letters indicate TEs that have been described in *Fusarium oxysporum* before.

LTR retrotransposons are similar to retroviruses and encode multiple enzymatic domains including Gag (a viral coat protein), protease, RNaseH, reverse transcriptase and integrase, flanked by long terminal repeats [14]. Within the LTR order we identified members of the Gypsy/Ty3 (27) and Copia/Ty1 (59) superfamilies, a novel class I TE named Yaret2, which encodes integrase (IPR001584), RNaseH (IPR012337), reverse transcriptase (IPR013103) and a Zinc-finger (IPR001878) domain, as well as two novel solo-LTR families. Solo-LTRs can be the result of intrachromosomal or intraelement recombination between the LTRs, thereby removing the internal domains and creating a solo LTR at the excision site [35]. Several of these LTR transposons have been previously recognized in *Fo* or in other pathogenic fungi. Nht2, for example, is also present on a LS chromosome of *Fusarium solani*[36].

LINE elements lack the LTRs that are characteristic for the retroviral-like class I TEs. In this order we identified 31 MGR583-like elements and 34 Yaret1 and Yaret1-like elements (24 and 10, respectively). MGR583 accompanies the effector gene *AVR-PITA* in some *M. oryzae* isolates [12]. The latter two are novel LINEs. Foxy (32 copies) represents the only TE of the SINE class on the pathogenicity chromosome. Foxy appears to be an active TE that is specific for *Fusarium* species [37]. Foxy elements are the most abundant class I TEs in *Fol* and they are evenly distributed over the pathogenicity chromosome and also throughout core chromosomes [1,38] (this manuscript). This dispersed distribution pattern is also apparent for the other class I TEs. Finally, we detected 10 copies of Marsu, which is a retrotransposon that cannot be classified as LTR, LINE or SINE. Copies of Marsu were first described in *Fo* f. sp. *phaseoli* where they were found downstream of the *FTF1* gene [27]. Ramos *et al*. speculated that the Marsu element might be responsible for gene duplication events of *FTF1*[27]. For most retrotransposon classes on the pathogenicity chromosome, we find only few full-length copies. Marsu is the marked exception: seven of the ten copies are full-length. Marsu copies are present in other *Fol4287* LS regions, and two copies reside on core chromosomes. Although we did not detect identical copies within the genome sequence of *Fol4287*, the presence of moderately divergent copies and many full-length copies suggest that Marsu elements have been active relatively recently.

**Figure 2:**
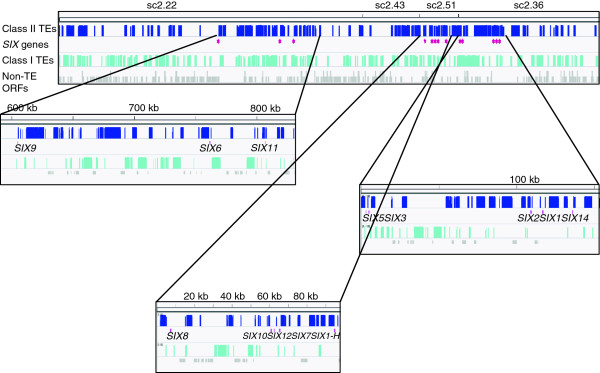
***SIX *genes reside in class II TE-enriched chromosomal subregions.** TE densities and *SIX* gene locations were displayed in the IGV Genome Browser. Supercontigs are ordered according to their position in the optical map of chromosome 14, ignoring gaps between them. The positions of the *SIX* genes are indicated by stars. Numbers above the enlarged windows refer to position (kb) in the respective supercontig (sc).

Compared to class I elements, class II elements are less evenly distributed on the chromosome and many aggregate in large chromosomal subregions (Figure 2). Class II elements are divided into two subclasses. Among subclass I we identified one Crypton copy. Cryptons encode a tyrosine-recombinase to cut and rejoin recombining DNA strands. They were first identified in human pathogenic fungi and were later found to be domesticated in vertebrates [39,40]. There are more Crypton copies present on other LS chromosomes, but none on core chromosomes. Within subclass II we identified nine Helitron copies. Helitrons are unusual class II TE; instead of a ‘cut and paste’ mechanism they transpose via a rolling-circle mechanism [14]. With this transposition mechanism they often capture host genes and thus contribute to genome evolution [15]. At least eight of the nine Helitron copies on the pathogenicity chromosome are intact; one is truncated by a sequence gap (Additional file 1). All copies are 99-100% identical in sequence, and there are intact Helitron copies on core chromosomes, suggesting that Helitrons are still active.

The best-represented order of class II TEs are the Terminal Inverted Repeat (TIR) TEs (Table 2). These TEs consists of a transposase ORF flanked by TIRs [14]. Among the TC1/mariner superfamily, we found multiple, diverse Fot lineages belonging to the pogo family. This finding confirms the previously shown preferred localization of pogo elements on LS chromosomes [41]. We observed a similar diversification of Hop elements belonging to the Mutator family. Five Hop classes are present with one to 13 copies, most of which are not full-length, although Hop has been shown to be active in *Fo*[42]. Most TE families, including three Folyt copies and 16 Hornet copies, belong to the hAT family. Folyt has been identified as an expressed and active transposable element in *Fol* by transposon trapping [43]. Hornet1 was discovered during analysis of transposons in *Fo* f. sp. *melonis*[44]. The only copy of the hAT transposon Drifter adjoins the truncated effector gene *SIX1-H*[45]. Overall, as previously shown for some genomic regions in *Fom*, class II TEs seem to preferentially insert into or close to each other, creating class II TE-enriched subregions on the *Fol* pathogenicity chromosome.

These subregions are also enriched for MITEs. MITEs are non-autonomous TEs, which basically consist of TIRs flanking a short non-coding DNA sequence. Three different classes of MITEs are present on the pathogenicity chromosome: 55 mimps (miniature Impalas), three Gimlis and 14 mFot5s (of which one is interrupted by a retrotransposon). MITEs require an associated transposase for transposition. Often, this associated transposase has similar TIRs [15]. For mFot5 transposition, two TEs encoding intact Fot5 transposases on the pathogenicity chromosome might facilitate transposition. Mimps are transposed by the Impala transposase, which was shown to be active in the melon pathogenic strain *Fo* f. sp. *melonis* by transposon tagging [46,47]. However, in *Fol4287* all three Impala copies, which reside on the pathogenicity chromosome, do not encode a full-length transposase, suggesting that mimps are presently not actively transposed in *Fol4287*. The large diversification of the mimp lineages with members of more than four families and without two identical copies also suggests that mimps are not presently active in *Fol4287*.

### Mimps are associated with promoters of *SIX* genes

**Figure 3:**
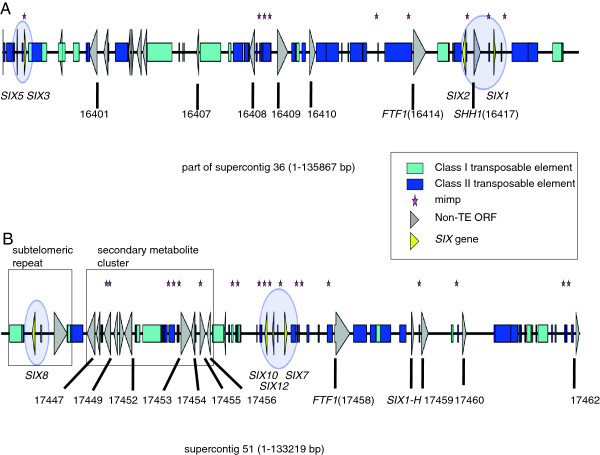
**Details of two class II TE-enriched chromosomal subregions with *SIX *gene mini-clusters.** Schematic representations of two equally large regions of the *Fol4287* pathogenicity chromosome: (**A**) part of supercontig 2.36 and (**B**) supercontig 2.51. Numbers represent FOXG gene numbers. Italic descriptions highlight interesting genes as reference points. Boxes indicate the telomeric repeat region and the putative secondary metabolite gene cluster. The genomic maps were drawn to scale.

*SIX* genes tend to reside in chromosomal subregions that are enriched for class II TEs, sometimes as mini-clusters (Figure 2, Additional file 1). For example, *SIX1* and *SIX2* form a mini-cluster with one intervening gene (salicylate hydrolase homolog (*SSH1*)) and two intervening mimps, flanked by another mimp and a Fot5 (Figure 3, see below). *SIX3* and *SIX5* form another mini-cluster with an intervening mimp, with nearby mFot5 and Fot5 fragments. This mini-cluster is flanked on both sides by inverted repeats, suggesting that this mini-cluster might be able to be transposed (Figure 3, Additional file 1).

**Figure 4:**
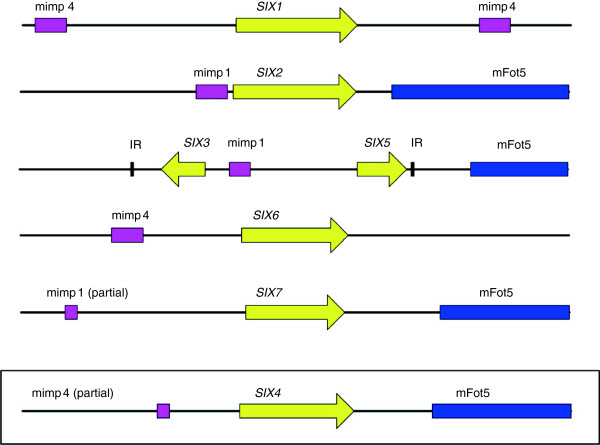
***SIX *genes harbor a mimp in their promoter.***SIX* gene loci including 1500 bp up-and downstream of the *SIX* ORF are drawn to scale. A miniature Impala (mimp, pink box) is present in the promoters of *SIX1 – SIX3* and *SIX5 – SIX7* in *Fol4287* (race 2 isolate). The *SIX4* locus (boxed) is not present in the race 2 isolate. It was sequenced and analyzed in the race 1 isolate *Fol004*. Downstream of some *SIX* genes is another MITE, mFot5 (dark blue box). Inverted repeats (IR) flanking the *SIX3/SIX5* locus are represented by thick black lines.

A closer inspection of the *SIX* gene promoters, which we pragmatically define as 1500 bp upstream of the start codon, revealed the presence of a mimp in the promoters of *SIX1, SIX2, SIX3, SIX5, SIX6* and *SIX7* (Figure 4). The mimp in the *SIX1* locus was revealed by re-sequencing, because in the *Fol4287* genome assembly there is a sequence gap upstream of the *SIX1* ORF. Another sequence gap separates a mimp from *SIX7*. We were not able to bridge this gap by PCR and therefore cannot rule out that the distance between the mimp and *SIX7* is bigger than 1.5 kb or that there is another mimp present that is closer to the *SIX7* start codon. The avirulence gene *SIX4/AVR1* of race 1 *Fol* strains, which is not present in *Fol* 4287 (race 2), also harbors a mimp in its promoter sequence (Figure 4). The pathogenicity chromosome harbors more than half of the mimps present in the *Fol4287* genome (Table 3). The other copies are mainly present on the three other LS chromosomes with the exception of four mimps on core chromosomes, as observed before [48]. Only a subset of the mimps on the pathogenicity chromosome is present in putative promoters (i.e. within 1500 bp of a predicted start codon). While *SIX1-7* all harbor a mimp in their promoter, only 8.3% of all annotated non-TE ORFs on the pathogenicity chromosome do so. This association of mimps with *SIX* gene promoters is highly significant (chi-square test p = 5.25E-16 for association by chance of mimps with the six known *SIX* genes on the *Fol4287* pathogenicity chromosome). Additional annotated ORFs with a mimp in the promoter region encode a bZIP transcription factor, an integral membrane protein, an alpha-N glucosaminetransferase, the Ftf1 transcription factor (2 copies), a catalase-peroxidase, the oxidoreductase Orx1, a homolog of the *Verticillium dahliae* avirulence protein Ave1, a methyltransferase, a cytochrome P450 and a squalene-hopene cyclase. The latter three genes belong to the putative secondary metabolite cluster that is co-expressed during plant infection (see above). Likewise, *FTF1* has previously been shown to be expressed during plant infection [27]. The catalase-peroxidase and Orx1 are secreted in the xylem sap of *Fol*-infected tomato plants [19] (this manuscript). Overall, therefore, mimps seem to be preferentially associated with the promoters of genes that are expressed during plant infection.

**Table 3:** **Distribution of mimps in the *Fol4287 *genome**

	**Number of mimps**	**Mimp per Mb**
pathogenicity chromosome	54	21,14
other LS chromosomes	45	3,26
core chromosomes	4	0,09

To see whether additional, potentially regulatory elements may be enriched in *SIX* gene promoters, we analyzed the promoter sequences of *SIX1*, *SIX2*, *SIX3*, *SIX5*, *SIX6* and *SIX*7 for enriched k-mers. Several overlapping 6 to 9mers were significantly enriched within these promoters. The most frequent of these form the sequence TCGGCAGTT (see Methods for details). Perfect matches to this sequence are present in the *SIX1* and *SIX3*/*SIX5* promoters. Compared to the entire gene set of the *Fol4287* genome, the association between the presence of at least one or two of the 6mers TCGGCA, GGCAGT and the 7mer GGCAGTT and the 1000 bp upstream region of effector genes appears to be significant (Additional file 3).

Finally, we also examined the 1500 bp downstream of the STOP codons of *SIX* genes. mFot5 is present downstream of *SIX2*, *SIX4, SIX5* and *SIX7* (Figure 4). The association of this MITE with the *SIX* genes is weaker than the mimp association with the *SIX* gene promoters, because it is not present downstream of all the *SIX* genes on the pathogenicity chromosome.

### *SIX1* gene expression is not dependent on the presence of a mimp in the promoter

**Figure 5:**
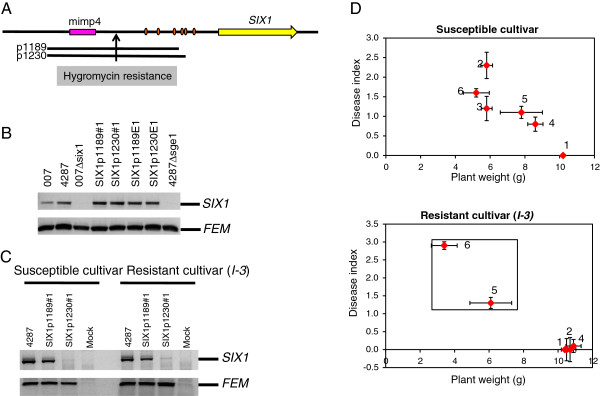
**Deletion of the mimp in the *SIX1 *promoter does not impair *SIX1 *expression, but a small region with a conserved motif is required for *SIX1 *expression during plant infection.** (**A**) Schematic representation of the *SIX1* locus. Black lines: deleted promoter fragments (deletion length in bp); pink box: mimp; yellow arrow: *SIX* gene; orange circles: sequence matching AAGTCGGCAGTT[AG] motif enriched in *SIX1-7* promoters. (**B**) *In vitro* expression of *SIX1* in the promoter deletion strains. Mycelium of the indicated *Fol* strains was collected after growth in minimal medium, From the collected mycelium RNA was extracted and RT-PCR was performed to detect transcripts of *SIX1* and, as control, the constitutively expressed *FEM* gene. (**C**) *In planta* expression of *SIX1* in the promoter deletion strains. Ten days old susceptible (without resistance genes) or resistant (encoding the I-3 resistance protein that recognizes Six1) tomato seedlings were inoculated with the indicated *Fol* strains or with water (mock). Roots were harvested 9 dpi (days post inoculation, RNA was extracted and RT-PCR was performed as described above. (**D**) Disease assay of tomato plants. Ten days old seedlings of susceptible or resistant tomato seedlings (as above) were inoculated with the indicated strains or with water. Three weeks after inoculation disease was scored by determining the plant weight above the cotyledons and by phenotypic scoring according to a disease index ranging from zero (no disease) to four (heavily diseased or dead plants). (1) mock, (2) WT, (3) SIX1p1189#1, (4) SIX1p1189#2, (5) SIX1p1230#1, (6) SIX1p1230#2. Please note that, SIX1p1189#1 was not included in the bioassay with the resistant cultivar, because it is not pathogenic (see D). A black box marks interactions where recognition of Six1 by I-3 is broken or where no disease is caused. Error bars indicate the 95% confidence interval of the mean.

We next wanted to know whether the mimp or the putative regulatory elements enriched in the *SIX* gene promoters are directly involved in transcriptional regulation of the *SIX* genes. To test this, we designed two constructs to replace different parts of the *SIX1* promoter with a hygromycin resistance cassette. Both deletion constructs included the mimp, the difference between the constructs being that the SIX1p1189 construct (1552 to 363bp upstream of the translation start site) deletes only three of the six conserved *SIX* gene promoter 12 mers, while the SIX1p1230 construct (-1552 bp to -323 bp) deletes five of these 12 mers (Figure 5A).

First, we tested whether *SIX1* was still expressed in the promoter deletion strains *in vitro*. Most *SIX* genes are not highly expressed *in vitro*, their expression is only switched on upon plant infection. However, a low amount of *SIX1* transcript is detectable *in vitro*[26]. To our surprise, *SIX1* was expressed in both SIX1p1189 and SIX3p1230 promoter deletion strains despite the absence of a large part of the *SIX1* upstream region (Figure 5B).

Six1 is recognized by the tomato resistance protein I-3 and triggers disease resistance in tomato plants carrying the *I-3*-gene, thereby prohibiting extensive fungal infection [49]. Upon plant infection, *SIX1* was only expressed from strains with the shorter SIX1p1189 deletion in both susceptible and resistant tomato cultivars. All transformed strains remained pathogenic towards tomato without Fol resistance genes, indicating that they were not affected in pathogenicity (Figure 5D). Consistent with the *in planta* expression pattern, only the wild type and strains with the SIX1p1189 promoter deletion were avirulent on the resistant tomato cultivar. In contrast, *I-3* tomato cultivars that were infected with *Fol* strains carrying the SIX3p1230 promoter deletion were diseased, indicating the absence or reduced accumulation of the Six1 avirulence protein (Figure 5D). Taken together, deletion of the mimp did not impair *SIX1* expression *in vitro* or in *in planta* and this mimp is therefore not required for transcriptional regulation of the *SIX1* gene. However, a promoter region including two TCGGCA elements appears to be required for *SIX1* expression during plant infection.

### *SIX3/SIX5* promoter deletions reveal complex regulation at this locus

**Figure 6:**
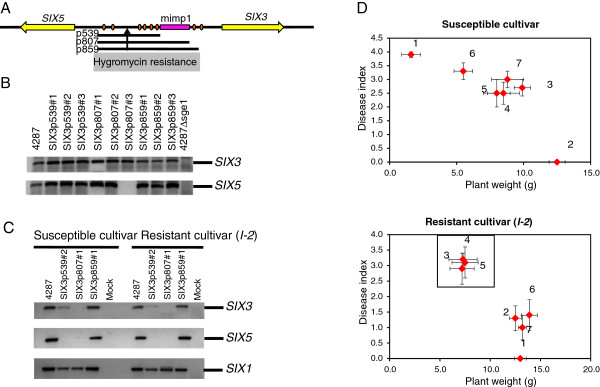
**Deletion of the mimp in the shared promoter region of *SIX3 *and *SIX5 *does not affect expression of the two genes. (A)** Schematic representation of the *SIX3/SIX5* locus. Black lines: deleted promoter fragments (length of the deletion in bp; pink box: mimp; yellow arrow: *SIX* gene; orange circles: sequence matching AAGTCGGCAGTT[AG] motif, which is enriched in *SIX1-7* promoters. (**B**) *In vitro* expression of *SIX3* and *SIX5* in the promoter deletion strains. Mycelium of the indicated *Fol* strains was collected after growth in minimal medium, RNA was extracted and RT-PCR was performed. (**C**) *In planta* expression of *SIX3* and *SIX5* in the promoter deletion strains. Roots of ten days old susceptible (without resistance genes) or resistant (encoding the I-2 resistance protein that recognizes Six3) tomato seedlings were inoculated with the indicated *Fol* strains or with water (mock). Roots were harvested 9 dpi (days post inoculation), RNA was extracted and RT-PCR was performed as described above. Expression of *SIX1* was used as a control. (**D**) Disease assay of tomato plants. Performed as above. Error bars indicate the 95% confidence interval of the mean. Numbers indicate the strains with which the tomato plants were inoculated: (1) mock, (2) *Fol007* (WT), (3) SIX3p539#2, (4) SIX3p807#1, (5) SIX3p807#2, (6) SIX3p859#1, (7) SIX3p859#2. A black box marks interactions where recognition of Six3 by I-2 is broken or where no disease is caused.

To further investigate the functional role of mimps in effector gene expression, we also designed promoter deletion constructs for the *SIX3-SIX5* locus. *SIX3* and *SIX5* share the same 1365 bp upstream sequence. This bidirectional promoter allowed us to test the expression of two different *SIX* genes with the same promoter deletion constructs. Like *SIX1*, *SIX3* is also recognized by a tomato resistance protein, I-2 in this case, and expression of *SIX3* and *SIX5* is low but detectable *in vitro*[18,26]. We designed three promoter deletion constructs: SIX3p539 (1095 to 520 bp upstream of the transcription start site), SIX3p807 (-1095 to -252 bp) and SIX3p859 (-1059 to -200 bp). SIX3p539 deletes six of the nine TCGGCA elements, but does not include the mimp, SIX3p807 includes the six TCGGCA elements and the mimp and SIX3p859 additionally deletes one more TCGGCA element (Figure 6A). Again, none of these promoter deletions impairs expression of *SIX3* or *SIX5 in vitro* (Figure 6B). During plant infection, a reduced level of *SIX3* mRNA was detected in *Fol* strains carrying the SIX3p539 deletion, but not in strains with the SIX3p807 deletion. *SIX5* is not expressed in either SIX3p539 or SIX3p807 deletion strains (Figure 6C). Remarkably, both *SIX3* and *SIX5* are expressed during plant infection in *Fol* strains carrying the most extensive promoter deletion, SIX3p859 (Figure 6C). With one exception, all tested strains were still able to cause disease on susceptible tomato cultivars and are thus not generally impaired in pathogenicity (Figure 6D). Only strains with the SIX3p859 deletion trigger a resistance response in tomato plants carrying the *I-2* resistance gene, while the *Fol* strains with the SIX3p539and SIX3p807 promoter deletions break I-2-mediated resistance, consistent with the Six3 protein not being produced by these strains (Figure 6C). Although in the *Fol* strains carrying the SIX3p539 promoter deletion a residual amount of *SIX3* transcript is present, these strains are virulent. This may be explained by the additional requirement of *SIX5* for *I-2*-mediated resistance (manuscript in preparation).

From this set of experiments in two *SIX* gene loci, we can conclude that the mimps are not required for regulation of *SIX* gene expression. On the other hand, deletion of a short region containing a single TCGGCA element in the promoter of *SIX1* abolishes *SIX1* expression suggesting that this motif might represent a transcription factor-binding site (Figure 5C). In contrast, at the *SIX3-SIX5* locus additional deletion of a region containing the same motif restores expression of both *SIX3* and *SIX5* during plant infection (Figure 6C).

### The presence of mimps in the promoters of *SIX* genes enables prediction of novel effector candidates

Next, we wanted to test whether we can use the consistent presence of a mimp in the upstream region of the *SIX* genes to predict novel effector candidates. We searched the *Fol4287* genome for the presence of a mimp TIR within 2 kb upstream of an ORF encoding a protein with an N-terminal signal peptide for secretion (as defined by SignalP). We also analyzed the xylem sap proteome of *Fol*-infected tomato plants by mass spectrometry to see which of the predicted effectors are secreted by the fungus during plant infection.

By the *in silico* search for mimp-association we predicted 16 effector genes in *Fol4287*, which are located on chromosomes 3, 6 and 14. These include three of the known *SIX* genes on the pathogenicity chromosome: *SIX2*, *SIX3*, *SIX6*. *SIX1* and *SIX7* were not identified because of sequencing gaps in the *Fol4287* genome assembly (see above). *SIX5* is a small gene comprising three exons. The first exon is unusually short and ends directly after the encoded signal peptide for secretion. Therefore, *SIX5* escaped signal peptide prediction (by SignalP) and thus was not identified with our approach.

Besides the known *SIX* genes, we identified nine genes coding for small secreted proteins and four genes coding for secreted enzymes with a mimp in the upstream region (Table 4). The latter comprise several multi-copy genes in the *Fol4287* genome: two (non-identical) *ORX1* copies, two copies of a gene coding for a catalase-peroxidase, two copies of a gene coding for a metalloprotease and three copies of a gene coding for an endo-polygalacturonase (Table 4). Both Orx1 and the catalase-peroxidase proteins were identified with mass spectrometry in the xylem sap of *Fol*-infected tomato plants, suggesting that they may play a role during plant infection.

**Table 4:** Novel effector candidates identified by searching for genes with a mimp IR in their promoter

**Gene description**			**Mimp in promoter**^**1**^		**SP**^**2**^	**Protein in xylem sap**
**Encoded protein**	**Chromosome**	**FOXG or genomic location**	**Identified in search**	**Distance mimp IR-ATG [bp]**		
***SIX *genes**						
Six1 (corrected)	14	FOXG_16418 (incorrect)	no^3^	1192	yes	yes
Six2	14	FOXG_16416	yes	211	yes	yes
Six3	14	FOXG_16398	yes	232	yes	yes
Six5	14	SC36[3273-3407]	no^4^	1132	yes	yes
Six6	14	FOXG_14246	yes	668	yes	yes
Six7	14	SC51[65216-65875]	no	(sequence gap)	yes	yes
**Novel effector candidates**						
Six8	14, 14	FOXG_17445, FOXG_16464	no^4^	109	yes	yes
Six8b	3, 3, 6, 6	SC18[1122404-122824],SC18[862700-863120], SC41 [221648-222068], SC21[219855-220275]	yes	1026, 1972	yes	no
Six9	14	FOXG_14223	yes	249	yes	yes
Six10	14	FOXG_17457	no^4^	384	yes	yes
Six11	14	SC22[806692-807024]	yes	322, 852	yes	yes
Six12	14	SC51[62415-62753]	no^5^	837	no	yes
Six13	6, 6	FOXG_17131 (5' extended), SC42[126863-127192]	yes	1971	yes	yes
Six14	14	SC36[135867-136180]	yes	211, 258, 681, 1215	yes	yes
FoAve1	14	SC36[201730-202101]	yes	788	yes	no
conserved secreted protein	14	FOXG_14254	yes	1312	yes	no
secreted protein	15	SC38[202206-202388]	yes	1717	yes	no
secreted protein	14	SC51[127492-128836]	yes	1236	yes	no
**Secreted enzymes**						
Orx1	14	FOXG_14258;FOXG_14236	yes	554	yes	yes
catalase-peroxidase	6, 14	FOXG_17130, FOXG_17460	yes	921	yes	yes
metalloprotease	3, 6	SC47[78991-79260], SC42[41025-41294]	yes	394	yes	no

^1^ distance between the mimp IR and the ATG start codon.

^2^ predicted signal peptide.

^3^ not identified because of a sequence gap in the genome assembly.

^4^ not identified because of a short first exon.

^5^ not identified due to absence of a signal peptide.

Next to these two enzymes, we obtained protein sequences for four of the nine predicted effector proteins from the xylem sap proteome. Additionally, we identified three more small proteins in the xylem sap of infected tomato plants that were not predicted by our *in silico* search. We named the genes for which we found the protein products in xylem sap *SIX8* - *SIX14*; one additional gene we named *SIX8b* for its high similarity to *SIX8* (Table 4). Upon inspection of the regions upstream of their respective genes we could always identify a mimp. *SIX8*, *SIX10* and *SIX12* were not found with the *in silico* search because no signal peptide was detected. Similar to *SIX5, SIX8* and *SIX10* have a short first exon and therefore the signal peptide was not recognized by SignalP. *SIX12* is an unusual effector gene: it does not encode a protein with a canonical signal peptide for secretion.

In contrast to the other *SIX* genes in *Fol*, *SIX8* is not a single gene, but is present in two copies on the pathogenicity chromosome (sc36 and sc51) and in subtelomeric regions on chromosomes 2, 3 and 7 in a repeated block of around 7400 bp. This block includes incomplete copies of the class II TEs Marsu and YahAT7, a Foxy and a gene encoding an unknown protein (Figure 3, Additional file 1). The repeated sequences flanking the *SIX8* genomic block on sc36 suggest that *SIX8* is present in a subtelomeric region. Furthermore, two copies of a related gene, *SIX8b*, are present on chromosomes 3 and 6 each in the *Fol*4287 genome. In total, there are nine *SIX8* and four *SIX8b* copies in the Fol4287 genome sequence. Both *SIX8* and *SIX8b* appear to be preceded by a complex structure of (partial) mimps and mimp IRs (Additional file 4).

Like the *SIX1-7* genes described above, the newly identified *SIX* genes, as well as several additional potential effector genes for which we did not find evidence for expression *in planta*, reside in class II TE-rich subregions (Figure 2, Table 4, Additional file 1). *SIX11* resides in a region that includes *SIX6*, three genes coding for conserved secreted proteins, one gene for a MFS transporter and one for a fumarate reductase/succinate dehydrogenase, a *FTF1* homolog and the *ORX1* gene. *SIX14* is part of a cluster containing *SIX1, SSH1* and *SIX2* (Figure 3). *SIX10* and *SIX12* make up a mini-cluster with *SIX7*. Similar to the *SIX3/SIX5* mini-cluster, *SIX12* is flanked on both sides by inverted repeats, suggesting that it may be mobilized by a transposase that recognizes these IRs. *SIX13* (FOXG_17131 - 5^′^ extended) is part of a duplicated region on chromosome 6 (sc 42), which is different from the interchromosomal duplication shared with chromosome 3.

Taken together, we have developed a method to predict novel effector genes in genomes of *F. oxysporum* based only on the following characteristics: (1) coding for small, secreted proteins, (2) harboring mimps or inverted repeats of mimps within 2 kb upstream of the start codon. We validated this method by mass spectrometric analysis of the xylem sap of *Fol*-infected tomato plants and confirmed *in planta* secretion of several predicted novel candidate effectors. These novel *SIX* genes represent ideal candidates for functional analysis.

## Discussion

### Effector genes on the *Fol* pathogenicity chromosome are associated with chromosomal subregions enriched in class II transposable elements

TEs dominate the *Fol* pathogenicity chromosome with large aggregates of class II TEs and more evenly distributed class I TEs. Interspersed within this TE-rich landscape are mostly single non-TE ORFs, a putative secondary metabolite cluster and the *SIX* gene mini-clusters. In many plant and fungal species with expanded genomes, retrotransposons are mainly responsible for genome expansion. Their mode of replication, which involves creating new copies during every transposition cycle, can rapidly increase genome size. Often, a single or few class I TEs account for the majority of TEs present in a genome. The maize genome, for example, consists of 76% class I TEs, with the Gypsy family element huck and the Copia element ji together accounting for nearly one quarter of the genome sequence [33]. Similarly, in the obligate fungal pathogen *Blumeria graminis* f. sp. *hordei*, the class I TE I (Line/Sine) alone occupies 17.2% of the entire genome space [32]. On the *Fol* pathogenicity chromosome, we do not observe such a massive expansion of retrotransposons. Instead, large aggregates of class II TEs are associated with genes involved in pathogenicity, such as the *SIX* gene mini-clusters. The tendency of class II TEs to concentrate in subchromosomal regions might result from recombination of their IRs with IRs of the same or a similar TE family. Occasionally, *SIX* genes might be trapped between the IRs and subsequently transposed together with the TE, resulting in the observed presence of *SIX* genes within class II TE-enriched chromosomal subregions. Support for this hypothesis stems from the observation that IRs directly flank *SIX12* and the *SIX3/SIX5* mini-cluster, although the transposase recognizing these IRs remains unknown. Similarly, the highly dynamic genomic location of the small, subtelomeric gene family *AVR-Pita* within the *M. oryzae* population has been attributed to the retrotransposons Inago-1 and Inago-2, which flank *AVR-Pita*. These are thought to be involved in multiple translocation events of *AVR-Pita*, thereby facilitating a cycle of loss and gain of recognition by rice cultivars encoding the cognate Pita resistance protein [12]. Next to retrotransposons, some DNA transposons have also been observed proximal to fungal effector genes. In *Leptosphaeria maculans*, putative effectors are clustered in AT-blocks together with three significantly over-represented TEs (one class I and two class II) [11]. Clustering of virulence genes might provide a selective advantage, because all captured genes experience the same genomic environment, e.g. an open or closed chromatin structure, thereby being simultaneously amenable for transcriptional regulation [50]. This might facilitate coordinated gene expression during plant infection.

### MITEs and *Fol* evolution

Mimps are always found within 1500 bp upstream the translation start site of *SIX* genes as well as upstream of several other genes that are expressed during plant infection. Mimps are uniformly small in size, ranging from 200 – 220 bp. Their central region has no coding capacity and is flanked by ~27 bp TIRs that resemble the TIRs of the Tc1/mariner transposase Impala [51]. Impalas have been shown to transpose mimps in a heterologous system [52]. However, none of the Impala copies in the *Fol* genome are intact, suggesting that mimps are not currently transposed in *Fol4287*. In the past there appear to have been several bursts of mimp amplification resulting in at least six mimp subfamilies present in *Fol*[51]. Strikingly, more than half of the mimps in *Fol4287* are present on the pathogenicity chromosome and the other mimps, with four exceptions, are restricted to the LS regions (Table 3) [51].

mFot5s reside downstream of the *SIX1/SSH1/SIX2*, the *SIX3/SIX5* and the *SIX10/SIX12/SIX7* mini-clusters as well as downstream of the solo *SIX9* gene (Figure 3, Additional file 1). mFot5 is also part of the putative secondary metabolite cluster that is co-expressed during *Fol* infection of tomato plants (Figure 3, Additional file 2). Downstream of *SIX11* is no mFot5, but a full-length Fot5. The same is true for *ORX1*, which encodes an oxidoreductase that is secreted by *Fol* during tomato infection. mFot5 is a pogo-like MITE, less than 500 bp long with TIRs similar to those of the Fot5 transposon. In contrast to the lack of intact Impalas for mimp transposition, *Fol4287* possesses around 64 intact Fot5 transposase ORFs that could mobilize mFot5s [41].

### What could be the function of mimps in promoters of effector genes?

Strikingly, mimps are not only present in *SIX* gene promoters, but also in the promoters of several other genes that are expressed during plant infection. Among these are the gene for the oxidoreductase Orx1 and two genes of the presumptive secondary metabolite gene cluster. One possible scenario is that the mimp is a domesticated TE, which has adopted a function as transcription factor binding site, perhaps for Sge1, the transcription factor regulating *SIX* gene expression [26]. We tested this by deleting fragments of varying length in the promoter of *SIX1* and the bidirectional, shared promoter of *SIX3* and *SIX5*. In a strain in which the mimp in the promoter of *SIX1* was deleted (*Fol4287*SIX1p1189), *SIX1* expression *in vitro* and *in planta* was the same as in wild type. Likewise, *SIX3* and *SIX5* expression was not affected in a strain in which the mimp was absent in their shared upstream region (*Fol4287*SIX3p859). Therefore, we can rule out a direct involvement of the mimp in transcriptional regulation of *SIX* gene expression.

We did, however, observe that the presence or absence of other promoter regions affect gene expression at the *SIX1* and the *SIX3*/*SIX5* locus. By comparing two different promoter deletions, we found that *SIX1* expression *in planta* requires a 41 bp region that includes one of the conserved TCGGCA elements that we found to be enriched in the *SIX* gene promoters (Figure 5). In contrast, *SIX3* and *SIX5* are not expressed from the two shorter promoter deletion strains, but expression of both genes is restored in the strain with the longest promoter deletion. The longest deletion additionally includes one of the TCGGCA elements (Figure 6A), which in this instance may mediate the action of a transcriptional repressor. The association of this element with upstream regions of effector genes is statistically significant (see Methods and Additional file 3 for details). Also, a perfect match to the extended motif (AAGTCGGCAGT) is present in the upstream regions of three genes encoding enzymes that we found in our analysis of the xylem sap proteome: *FOXG_11769* on chromosome 10, encoding a glycosyl hydrolase and the closely related *FOXG_14234* on the pathogenicity chromosome and *FOXG_17180* on an unpositioned scaffold, encoding a peroxidase-catalases. Nevertheless, the function of this putative regulatory sequence remains to be established.

Interestingly, *SIX1* as well as *SIX3* and *SIX5* were weakly expressed in all promoter deletion strains *in vitro*, but not *in planta*. In absence of a plant host, *SIX* gene expression is usually very low [26], while it is strongly induced upon plant infection [53]. *SIX* genes are only needed during plant infection; therefore the fungus might actively suppress *SIX* gene expression in the absence of a plant host. One way of suppressing gene expression is by modification of chromatin to a repressive, closed state. Repressive chromatin structures often involve histone modifications such as H3K9 methylation [54]. One origin of such repressive chromatin structures is TE silencing, often guided by small RNAs transcribed from the TE [55]. In plants of the Solanaceae family, MITEs proximal to *R* gene loci were shown to produce small RNAs that are recruiting the histone methylation machinery for TE silencing resulting in the formation of closed chromatin [56]. TE silencing of the MITEs surrounding the SIX genes might likewise create a repressive chromatin environment, which may serve as a first layer of *SIX* gene regulation. Upon stress, such as during plant infection, TEs might be derepressed as shown for the oomycete pathogen *Phythophthora ramorum*[57], thus creating an open chromatin structure. Binding of transcriptional activators or repressors would be possible in an open chromatin state and provide the basis for a second layer of regulation of *SIX* gene expression.

### Identification of novel effector candidates

We identified eight novel (candidate) effector genes based on the presence of a mimp in their promoters and/or the presence of their protein product in xylem sap of infected plants. Five of these genes (*SIX8b*, *SIX9*, *SIX11*, *SIX13*, *SIX14*) were identified by the *in silico* search and validated by the analysis of xylem sap from infected tomato plants. Like the previously identified *SIX1* and *SIX7* genes, *SIX10* escaped the *in silico* identification due to a sequencing gap close to its promoter. *SIX12* encodes an unusual effector lacking a recognizable N-terminal signal peptide for secretion via the classic Endoplasmatic-Reticulum/Golgi route. Nevertheless, the Six12 protein is present in the xylem sap of infected tomato plants and therefore might be secreted via an unconventional protein secretion route [58]. *SIX13* encodes the only effector known so far that is located on a LS chromosome other than the pathogenicity chromosome. We also identified *FoAVE1* as a gene harboring a mimp in its promoter, but we did not detect the FoAve1 protein in the xylem sap nor detected *FoAVE1* mRNA in infected plants (results not shown). Apparently, in the strains used here *FoAVE1* is not expressed during infection, although it was shown to be able to elicit *Ve1*-mediated resistance in a heterologous system [59]. *FoAve1* might be part of a silent effector reservoir together with the other three genes that encode small, secreted proteins and harbor a mimp in their promoters, but are not expressed during infection.

Some of the genes we identified here have been subject to gene or segmental duplications. *ORX1* is present in two similar but not identical copies on the pathogenicity chromosome (FOXG_14258, FOXG_14236; Additional file 1). Two other genes, FOXG_17460 on the pathogenicity chromosome and FOXG_17130 on chromosome 6, both encode a metalloprotease. Apart from a missing 3’ end of FOXG_17460 due to a sequencing gap, the two genes and their promoters are identical, indicating a recent duplication event. *SIX13* is also duplicated. In both cases the duplicated gene copies do not harbor a mimp in their promoter. *SIX8b* is present in four identical copies due to an intra- and interchromosomal segmental duplication within and between chromosome 3 and 6 [1]. This duplicated chromosomal segment corresponds to another small chromosome that can be transferred horizontally from the strain *Fol007*[1]. Progeny strains possessing both the pathogenicity chromosome and the other small chromosome are more aggressive towards tomato than progeny strains with only the pathogenicity chromosome. At present, we do not know which gene(s) on the small chromosome (corresponding to sc18 in the *Fol4287* genome) contributes to pathogenicity towards tomato – Six8b was not found in the xylem sap proteome.

In summary, mimps are associated with the promoters of all small *in planta* secreted proteins, as well as several enzymes. Our strategy for *in silico* detection of effector genes in *F. oxysporum* is limited by three factors: 1) imperfect conservation of the IRs of a mimp, 2) sequencing gaps in the genome assembly and 3) absence of a canonical N-terminal signal peptide for secretion. The impact of first two factors may be alleviated by more advanced methods for mimp detection and genome assembly. The third factor, absence of a canonical signal peptide, can be either due to secretion via an unconventional route or to a failure of SignalP to predict a signal peptide, as was the case for *SIX5* or *SIX8*. In the latter case, incorporation of gene structure (intron/exon) predictions or transcript sequences will be helpful. Overall, our approach presents a powerful tool to predict novel effectors and other virulence factors in *F. oxysporum*.

## Conclusions

Class II TEs are much less evenly distributed over the *Fol* pathogenicity chromosome than class I TEs. Effector genes reside as single genes or mini-gene clusters within class II TE-enriched chromosomal subregions. Two MITEs are closely associated with effector genes. A (partial) mimp is always present in effector gene promoter regions and a mFot5 is frequently present downstream of the effector gene mini-clusters. We could exclude a direct involvement of the mimp in effector gene expression by making promoter deletion strains for two effector gene loci followed by gene expression analysis and tomato pathogenicity assays. Overall, the unique association of effector genes and mimps allowed us to develop a method to successfully predict candidate effector genes. For most of these genes, the corresponding protein was found by mass spectrometry in the xylem sap of tomato during *Fol* infection. Our method can easily be extended to predict novel effector genes in *Fo* strains with different host specificities.

## Methods

### Plant lines and fungal strains

The following tomato (*Solanum lycopersicum*) lines were used (*Fol* resistance genes between brackets): 90E402F (I-1) [60,61]; 90E341F (I-2) [62] and E779 (I-3) [60], C32 (no I gene) [63]. The following *Fol* strains were used: *Fol007* (race 2), *Fol4287* (race 2), *Fol004* (race1), *Fol4287*SIX1p1189, *Fol4287*SIX1p1230, *Fol4287*SIXc3p539, *Fol4287*SIX3p807, *Fol4287*SIX3p859, *Fol4287*Δsge1, *Fol007*Δsix1. See Rep *et al*. [45] for a more detailed description of the wild type *Fol* strains.

### Identification and annotation of TEs

Repetitive DNA elements were identified by performing a self-BLASTN against the *Fol4287* genome sequence, then using a custom PERL script (Amyott S.G. et al.,. manuscript in preparation), which identifies multi-copy sequences and sorts these repeated sequences into non-redundant families. Additional TEs with terminal inverted repeats were identified by search the *Fol4287* genome for inverted repeats of at least 19 bp encompassing at most 5 kb of sequence. Blast was used to find all instances (full length or partial) in the *Fol4287* genome. Additional file 5 contains prototypes for all newly identified TEs.

### Promoter deletion constructs

The promoter deletion constructs for the *SIX1* and *SIX3/SIX5* promoters were made by PCR amplification sequences of the sequences flanking the part of the promoter that was to be deleted for homologous recombination, and their insertion in front of and behind the hygromycin resistance gene in the vector pRW2h (see below). For *SIX1*: for both deletion constructs a 829 bp upstream fragment was cloned into pRW2h [64] between the *Pac*I and *Acc*65I sites and a1093 bp and 1052 bp downstream fragment, for the SIX1p1189 construct and the SIX1p1230 construct respectively, were cloned into pRW2h between at the *Xba*I site. For *SIX3*: a 1001 bp upstream fragment was cloned into pRW2h between the *Pac*I and *Acc*651 sites and a 1332 bp (SIX3p539), a 1064 bp (SIX3p807) and a 1012 bp (SIX3p807) downstream fragment was cloned into the *Xba*I site of pRW2h. Transformation of these constructs to *Fol4287* was done with Agrobacterium as described earlier [65].

### Tomato disease assay

Ten days old tomato seedlings were inoculated with a fungal spore suspension and disease was scored after three weeks as described earlier [49]. The outcome of the disease assays was quantified in two ways: 1) average plant weight above the cotyledons and 2) phenotype scoring according to a disease index ranging from zero (no disease) to four (heavily diseased or dead) [49].

### *Fol* gene expression analysis

For *in vitro* expression analysis, *Fol* mycelium was harvested after three days growth at 25°C and 175 rpm in minimal growth medium (3% sucrose, 1% KNO3 and 0.17% yeast nitrogen base without amino acids and ammonia). For *in planta* expression analysis, ten days old tomato seedlings were inoculated with fungal spores suspensions as described above and roots were sampled eight or nine days after inoculation. From the collected material, RNA was isolated using TRIzol reagent (Gibco) followed by phenol-chloroform extraction. The isolated RNA was used to make cDNA using Promega Rnasin (ribonuclease inhibitor) and Gibco Superscript II RNaseH Reverse transcriptase according to the manufacturer’s instructions. Primers used for RT-PCR analysis are listed in Additional file 6.

### Identification of novel effector candidates

Based on published sequences of prototypes of mimp1-4 as well as mimps present in promoters of SIX1-7, a consensus mimp 3’ IR was defined as ‘TT[TA]TTGCNNCCCACTG’. A PERL script was used to find instances of this pattern in the genome sequence of *Fol4287*, downloaded from the broad website (http://www.broadinstitute.org/annotation/genome/fusarium_group/MultiHome.html). For 150 of the 158 matches to this pattern, the next dinucleotide was ‘TA’, which is the required target site for mimps and Impalas.

For each mimp IR match, all open reading frames (ORFs) starting with an ATG and of at least 25 codons within 2000 bp downstream of the IR were selected. The ORFs were translated and the translation products submitted to signal peptide prediction by SignalP (http://www.cbs.dtu.dk/services/SignalP/). If positive, the instance was recorded (mimp IR sequence, translation product of ORF and their positions in the *Fol4287* contig). The sequence surrounding this instance was retrieved and manually inspected to define the full ORF of the candidate effector gene.

### *In silico* promoter analysis

To find potential regulatory elements in promoters of effector genes, we first identified enriched k-mers in the concatenated upstream regions of *SIX1*, *SIX2*, *SIX3*, *SIX5*, *SIX6* and *SIX*7, using Compseq (http://emboss.bioinformatics.nl/cgi-bin/emboss/compseq). As upstream regions we used here the sequences between the upstream mimp and the ATG, to avoid identification of sequences within mimps (especially the conserved inverted repeats). We looked for enriched 6mers, 7mers, 8mers and 9mers in both strands. Among the most frequent 6mers and 7mers, we found two classes: (1) A diversity of AT-sequences and (2) a small set of overlapping sequences that were present in one or more instances in all – or all but one – upstream regions. The most frequent sequence elements of the second class were the 6mers TCGGCA (16), GGCAGT (14), CGGCAG (11) and GCAGTT (11) and the 7mers GGCAGTT (11), TCGGCAG (9) and CGGCAGT (7). The overlap of these 6mers and 7mers is the 9mer TCGGCAGTT. This is also the most frequent 9mer, which occurs 6 times in the upstreams regions, namely in those of *SIX1* (2X), *SIX3* (1X) and *SIX5* (3X). Except two palindromic AT-rich sequences (TTTTAAAA and TATATATA), the most frequent 8mers matched this 9mer: TCGGCAGT (6), CGGCAGTT (7), or extend it: GGCAGTTA (6). Two other frequent 8mers extend the sequence on the other end: **AAG**TCGGC (4) and **AG**TCGGCA (4). Additional overlapping, enriched 9mers (each occuring 3 times) further extend the combined sequence to the consensus AAGTCGGCAGTT[AG]A.

To assess the significance of the occurrence of this motif in the upstream regions of effector genes, we analysed the 17708 upstream regions of Fol4287 genes, defined as 1000 bp upstream of the predicted translational start codon. This analysis is summarized in Additional file 3. Briefly, we calculated the probability that the frequency with which the two most frequent (and overlapping) 6mers, TCGGCA and GGCAGT, and to the most frequent 7mer, GGCAGTT (a one base extension to the second 6mer), occur at least once or twice in the upstream regions of effector genes is by chance association. We did this both for the original set of effector genes used to find the pattern (*SIX1-3* and *SIX5-7*), and for the entire set of identified effector genes (including *SIX8b*). All p values were lower than 0.05. The weakest association was between at least one TCGGCA element and the original set (p = 0.024) and between at least one GGCAGT and the entire set (p = 0.015). Association with at least twice occurences were more significant in all cases. Association with the entire set was slightly more significant for the TCGGCA element (at least once or at least twice) and for at least twice occurences of the GGCAGT element. The other associations were weaker with the entire set of effector genes.

### Xylem sap collection, mass spectrometry and label free quantitative proteomics

*Fol007* was used for tomato inoculation. Four-week-old tomato plants C32 were inoculated, after removing part of the root system, with a *Fol* spore suspension (5 × 10^6^ spores mL^−1^) or with water as a negative control, and potted. Fourteen days post inoculation (dpi), xylem sap was collected as described [66,67]. Briefly, stems were cut below the second true leaf and the plant was placed in a horizontal position. Then, for minimal 6 h sap bleeding from the cut surface was collected in tubes placed on ice. The collected xylem sap was stored at -20°C.

For label-free protein quantification 25 plants per inoculum were inoculated with Fol007 or water. Xylem sap was isolated as described above from four independent biological replicates. A fraction of the sap was used for immunoblotting, the remainder was concentrated with a Centricon plus-70 (Millipore) unit to a final volume of 200-300 μl. The protein concentration was determined with the bicinchoninic acid method (Sigma). After trichloroacetic acid/aceton precipitation protein isolated from inoculated plants with water or *Fol007* was dissolved in sample buffer at equal concentration (1.5 μg/μl) and 30 μl per sample was loaded on the SDS-PAGE. SDS–PAGE was performed with Hoefer Mighty Small SE250 minigel equipment (Amersham Biosciences, AB, Uppsala). After a short run, the Coommassie PageBlue™ (Fermentas) was used to visualize the proteins in the SDS-PAGE. For each xylem sap sample one gel slice containing all proteins was cut from the Coomassie-stained gel. In-gel digestion was performed as described by Rep et al. [67]. The peptides obtained after this digestion were analyzed by nanoLC-MS/MS as described by Lu, et al [68]. Raw data from the LTQ-Orbitrap were analyzed with MaxQuant software [69,70] to identify the proteins and allow label-free relative quantification. MaxQuant 1.1.36 settings were used according to the description by Peng, et al [71]. The *Fol* protein database used for the analysis was obtained from Fusarium Comparative Genome website (http://www.broadinstitute.org/annotation/genome/fusarium_group/MultiHome.html) and supplemented by adding the sequences of known Six proteins that are not annotated in the public database. A “contaminant” database was used that contains proteins such as trypsin and human keratins [71]. Bioinformatics analysis of the MaxQuant workflow and the statistical analysis of the abundances of the identified proteins were performed using Perseus (available at http://www.MaxQuant.org) [70]. Only proteins identified with at least two peptides, of which one should be unique, were kept.

## Competing interests

The authors declare that they have no competing interests.

## Authors’ contributions

SMS and MR designed experiments, carried out the studies and analysis and wrote the manuscript. PMH helped generate the expression data and tomato pathogenicity test. IS made the promoter deletion constructs. LM and SB generated the MS analysis of the tomato xylem sap proteome. SA helped identifying transposable elements in the *Fol4287* genome. BC generated expression data of the secondary metabolite cluster. All authors read and approved the final manuscript.

## Supplementary Material

Additional file 1 **Detailed annotation of the *Fol4287* pathogenicity chromosome.** Click here for file

Additional file 2 **A putative secondary metabolite gene cluster of *Fol* is expressed during tomato infection.** Roots of ten days old susceptible (without resistance genes) tomato seedlings were inoculated with conidiospores of *Fol004*. Roots were harvested 8 dpi (days post inoculation). From the collected roots RNA was extracted and (RT-) PCR was performed to detect transcripts of the indicated genes. Numbers represent FOXG numbers of the *Fol4287* reference genome. Marker sizes are indicated on the right. C: cDNA, G: genomic DNA. Click here for file

Additional file 3 Significance of the association between the TCGGCA element and upstream regions of effector genes. Click here for file

Additional file 4 **Complex repeat structure in *SIX8, SIX8b *and *SIX14 *upstream regions.** The most upstream sequence shared between the *SIX8* and *SIX8b* loci (dark grey, blue and green highlighted) is more similar between *SIX8* and *SIX8b* loci than the coding sequences and the immediate upstream sequences (light grey). The *SIX8b* upstream region is the most complex. Compared to that of *SIX8*, there are: (a) a mimp4 insertion, (b) a Han insertion, (c) an inversion and duplication (indicated with < signs), (d) a mimp1 insertion, (e) a partial mimp3 and (f) an extra sequence that includes an mFot5. A total of 9 mimp-related inverted repeats are present, of which two are interrupted by a TE. Part of the *SIX14* upstream region is almost identical to a part of the *SIX8b* upstream region (green/blue highlighted including the mimp4) – except that the Han insertion is missing in the *SIX14* locus. In both cases, a mimp1 is present immediately downstream of this region but, though similar in sequence, these mimp1 insertions appear to be independent. Blue capital letters: effector ORF (introns in lower case); Green capital letters: mimp; Dark red capital letters: mFot5; Orange capital letters: Han; Gray highlight: shared between *SIX8* and *SIX8b* loci only; Light gray highlight: similarity between *SIX8* and *SIX8b* upstream (leader/promoter) sequences; Blue highlight: mimp-like inverted repeat sequence, present one or more times in *SIX8*, *SIX8b* and *SIX14* loci (numbers of likely orthologous sequences correspond between the three loci – note that mimp-IR1 does not conform to the consensus sequence for mimp inverted repeats); Green and dark green highlight: sequences present one or more times in *SIX8*, *SIX8b* and *SIX14* loci; Yellow highlight: TGCCGA motif; Bold: target site duplications associated with TE insertions. Click here for file

Additional file 5 **Newly identified TEs of *Fol.*** Click here for file

Additional file 6 Primers used in this study. Click here for file
