# Supplementary figures and images for: MITEs in the promoters of effector genes allow prediction of novel virulence genes in Fusarium oxysporum
Source: BMC Genomics. 2013 Feb 22;14:119. doi: 10.1186/1471-2164-14-119 (PMC3599309; doi:10.1186/1471-2164-14-119)

## Slide 1
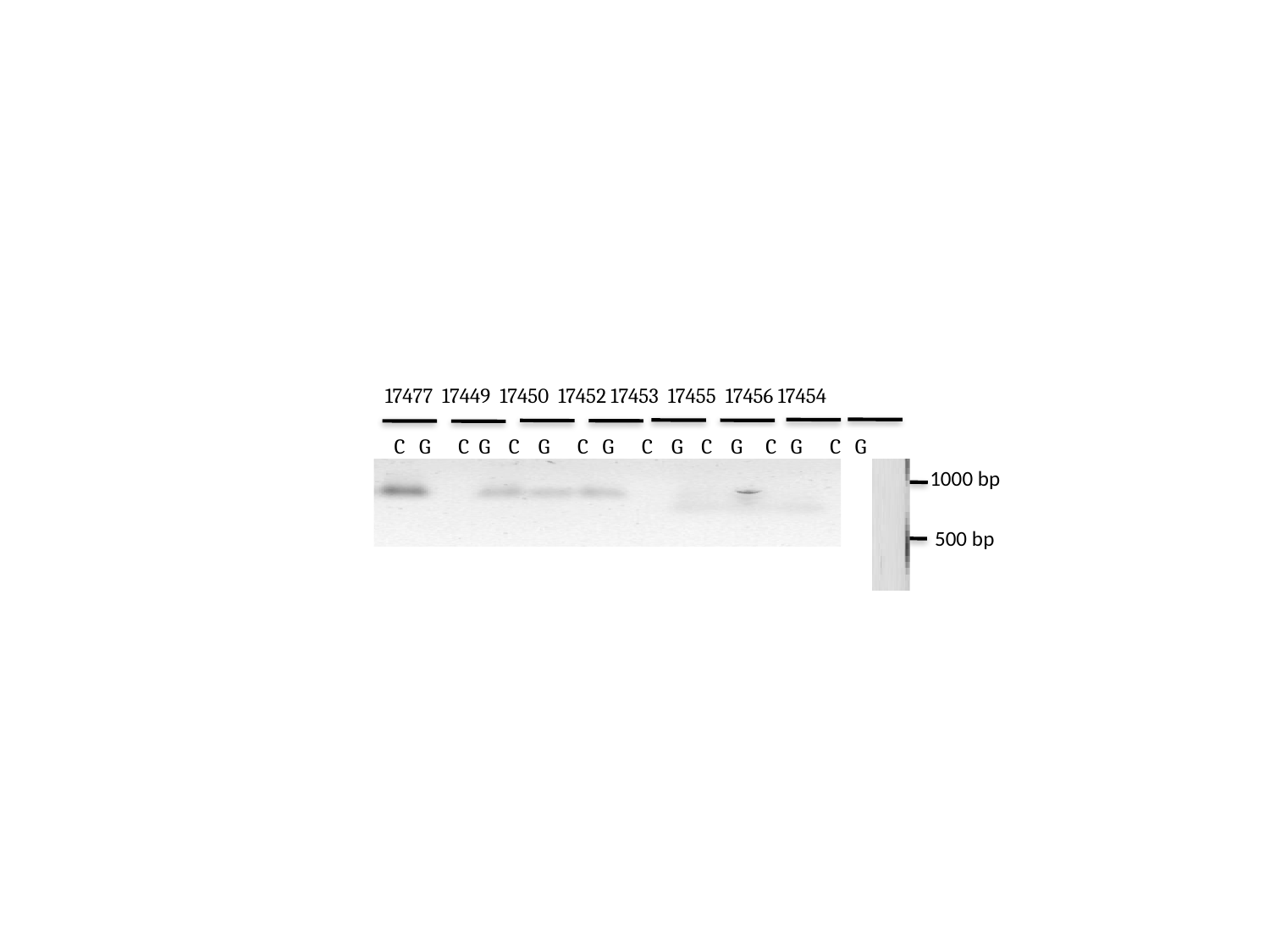

17477 17449 17450 17452 17453 17455 17456 17454
 C G C G C G C G C G C G C G C G
1000 bp
 500 bp

Supplement: Additional file 2 — A putative secondary metabolite gene cluster of Fol is expressed during tomato infection. Roots of ten days old susceptible (without resistance genes) tomato seedlings were inoculated with conidiospores of Fol004. Roots were harvested 8 dpi (days post inoculation). From the collected roots RNA was extracted and (RT-) PCR was performed to detect transcripts of the indicated genes. Numbers represent FOXG numbers of the Fol4287 reference genome. Marker sizes are indicated on the right. C: cDNA, G: genomic DNA. [file 1471-2164-14-119-S2.pptx]
