# Supplementary material for: MITEs in the promoters of effector genes allow prediction of novel virulence genes in Fusarium oxysporum
Source: BMC Genomics. 2013 Feb 22;14:119. doi: 10.1186/1471-2164-14-119 (PMC3599309; doi:10.1186/1471-2164-14-119)
Supplement: Additional file 3 — Significance of the association between the TCGGCA element and upstream regions of effector genes. [file 1471-2164-14-119-S3.doc]

**Significance of the association between the TCGGCA element and upstream regions of effector genes**

As described in Methods, overlapping enriched k-mers in the promoters of *SIX1*, *SIX2*, *SIX3*, *SIX5*, *SIX6* and *SIX*7 overlap to form the consensus sequence AAGTCGGCAGTT[AG]A. In total, we found 23 instances of the 6mers TCGGCA or GGCAGT in the above-mentioned promoters. These 23 instances add up to the following logo (<http://weblogo.berkeley.edu/logo.cgi>):


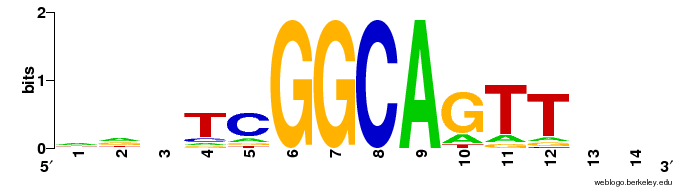


To assess the significance of the occurrence of this motif in the upstream regions of effector genes, we analysed the 17708 upstream regions of Fol4287 genes, defined as 1000 bp upstream of the predicted translational start codon. These were downloaded from the Broad Institute (<http://www.broadinstitute.org/annotation/genome/fusarium_group/Downloads.html>). The only two matches to the full consensus (AAGTCGGCAGTT[AG]A) are in the upstream region of *SIX3 (FOXG_16398)* and *FOXG_05964*. The only gene with three matches to the most conserved 9mer (TCGGCAGTT) in the 1000bp upstream region is, again, *SIX3* (*SIX5* is presently not annotated in the genome sequence). We found four upstream regions with two instances of this 9mer: those of *SIX1* (which is misannotated in the genome sequence), *FOXG_02437* and *FOXG_10020*, and the shared upstream region of *FOXG_15332* and *FOXG_15333*.

We next counted the matches to the two overlapping 6mers most frequently found in our initial analysis, TCGGCA and GGCAGT, and to the most frequent 7mer, GGCAGTT (a one base extension to the second 6mer). The 17708 upstream regions (both strands) have the following number of matches to these sequences:

| # matches | 0 | 1 | 2 | 3 | 4 | 5 | 6 | 7 | 8 | 9 |
| --- | --- | --- | --- | --- | --- | --- | --- | --- | --- | --- |
| TCGGCA | 10883 | 5099 | 1394 | 275 | 42 | 9 | 5 | 1 |  |  |
| GGCAGT | 11252 | 5051 | 1164 | 216 | 23 |  |  |  |  | 2 |
| GGCAGTT | 15475 | 2080 | 140 | 11 | 2 |  |  |  |  |  |

The number of matches of these sequence elements in effector gene promoter regions (1000 bp, both strands) are as follows:

|  | TCGGCA | GGCAGT | GGCAGTT |
| --- | --- | --- | --- |
| *SIX1* | 5 | 3 | 3 |
| *SIX2* | 0 | 1 | 1 |
| *SIX3* | 5 | 4 | 4 |
| *SIX4* | 2 | 3 | 1 |
| *SIX5* | 4 | 4 | 4 |
| *SIX6* | 2 | 1 | 1 |
| *SIX7* | 1 | 1 | 0 |
| *SIX8* | 5 | 2 | 0 |
| *SIX8b* | 1 | 0 | 0 |
| *SIX9* | 0 | 0 | 0 |
| *SIX10* | 0 | 0 | 0 |
| *SIX11* | 1 | 1 | 1 |
| *SIX12* | 2 | 0 | 0 |
| *SIX13* | 0 | 2 | 0 |
| *SIX14* | 1 | 0 | 0 |

From these numbers, we calculated the Chi square probability that the frequency with which these sequences occur at least once or twice in the upstream regions of effector genes is by chance association, both for the original set of effector genes used to find the pattern (*SIX1-3* and *SIX5-7*), and for the entire set of identified effector genes (including *SIX8b*):

|  | | *SIX1-3, SIX5-7* (n=6) | | | *SIX1-14* (n=15) | | |
| --- | --- | --- | --- | --- | --- | --- | --- |
|  |  | Exp. | Obs. | p | Exp. | Obs. | p |
| TCGGCA | at least once | 2.70 | 5 | 0.024 | 5.40 | 11 | 2.1E-3 |
|  | at least twice | 0.68 | 4 | 2.6E-6 | 1.36 | 7 | 3.8E-7 |
| GGCAGT | at least once | 2.19 | 6 | 1.2E-3 | 5.47 | 10 | 0.015 |
|  | at least twice | 0.48 | 3 | 1.3E-4 | 1.19 | 6 | 4.3E-6 |
| GGCAGTT | at least once | 0.76 | 5 | 1.8E-7 | 1.89 | 7 | 7.1E-5 |
|  | at least twice | 0.052 | 3 | 1.2E-38 | 0.13 | 3 | 1.2E-15 |
