# Supplementary material for: MITEs in the promoters of effector genes allow prediction of novel virulence genes in Fusarium oxysporum
Source: BMC Genomics. 2013 Feb 22;14:119. doi: 10.1186/1471-2164-14-119 (PMC3599309; doi:10.1186/1471-2164-14-119)
Supplement: Additional file 4 — Complex repeat structure in SIX8, SIX8b and SIX14 upstream regions. The most upstream sequence shared between the SIX8 and SIX8b loci (dark grey, blue and green highlighted) is more similar between SIX8 and SIX8b loci than the coding sequences and the immediate upstream sequences (light grey). The SIX8b upstream region is the most complex. Compared to that of SIX8, there are: (a) a mimp4 insertion, (b) a Han insertion, (c) an inversion and duplication (indicated with < signs), (d) a mimp1 insertion, (e) a partial mimp3 and (f) an extra sequence that includes an mFot5. A total of 9 mimp-related inverted repeats are present, of which two are interrupted by a TE. Part of the SIX14 upstream region is almost identical to a part of the SIX8b upstream region (green/blue highlighted including the mimp4) – except that the Han insertion is missing in the SIX14 locus. In both cases, a mimp1 is present immediately downstream of this region but, though similar in sequence, these mimp1 insertions appear to be independent. Blue capital letters: effector ORF (introns in lower case); Green capital letters: mimp; Dark red capital letters: mFot5; Orange capital letters: Han; Gray highlight: shared between SIX8 and SIX8b loci only; Light gray highlight: similarity between SIX8 and SIX8b upstream (leader/promoter) sequences; Blue highlight: mimp-like inverted repeat sequence, present one or more times in SIX8, SIX8b and SIX14 loci (numbers of likely orthologous sequences correspond between the three loci – note that mimp-IR1 does not conform to the consensus sequence for mimp inverted repeats); Green and dark green highlight: sequences present one or more times in SIX8, SIX8b and SIX14 loci; Yellow highlight: TGCCGA motif; Bold: target site duplications associated with TE insertions. [file 1471-2164-14-119-S4.doc]

SIX8 locus

gtgtttaccactgccacaggccttgatcgtggcacagtcattgggggttc

ccgtctcagggtatttcatacatcggcaccgatcccagtcttctgccgag

gcagtagttaagtacctaaaagcttaattagaaatcgagcaaatggggtt

cgaatctactctttacagcaaaaccattgcaagcgcgggattcatggttg

gtaacacgagaagttagggtattgaagttcaggcgttgcggagacaacta

atgagtctttccgatattttctgaattgggtcgtatctaccgtctaacca

acggaccagacgtgcggcaaggagaagtgtttctctttgtgaggtaaaat

atgttcgtcgcttaggattaaaacttcacttccggcgatagagtaatagt

tgggcagaacgcaggtttctcttcctttaagtacttaaagcacattgata

atccatatattattaataaccctgttatagta**ta**ccgtgtggtgcaataa mimp-IR1

gtttgaggatctttgaaggtcgtgttcatctgccgatttgatttatctgc

cggtct**tg**tgtactagtatttaggttaaagcggcagaactaccttttact

tgtattcttttagtgtttcggcatatagtttaagctttcattcatattac

agtggggtgc**aataa**gtttgaggatctttgaaggtaccgaagcagccggt mimp-IR2

ctagccggattagatcctgttcatctgccgatttgatttacagtatgtta

cagggtaattatactttatactctacccgtatcaaaagaacagcttttag

gagagatttaagcagcgaagtgcgccatataagactaaagatgtatttaa

tgatggactctccctctatttcctcgttcccactcattcttttgtaccgc

accaagcgtccgggcttaccttcccctcagttccacttcctaagtaagtc

tttacgtccttacttataccctctcaatactatatacggcagctATGCAA

CCCTTACGCATTCTTTTACTTTTCCCCCTAGCTGTCTCTGTGGCTGCTAC

GCCTATTGATAAGAGgtatgtctgattctcatgaatacatctgtcacaca

ttaatatataaagCCTCGACCAAGCCGCCACTATTGAAGAAACTGTTCAT SIX8

CAACCTCATTCTCATGACGAGAGAGCCCTTGTCGAACGgtaggtaattta

gtatagatttgacgttgcttctaatgtaaatcacagCGACACCAGTGGCA

TTTTACTCGCCTGCATAACAGGTGCCGGTTCGGCGTTTCAGGCATATGCA

GGATGCTATCTCACTGCATTTCGTAATGACCCGCGCACTCTTACGTTGAG

GATGGACAAAACTAGGGGTGAACGGATATCGAACGTGCTTGTTATTTTGA

GCGGGGGAGCGCTCTCACATGCGGTGGAGGAGGTAGTCCAAATCGCGCCG

GGTGCGGTGAGGAACTTAGCTACTTTAGGTGCATCGACTGTCCAGTTTCT

ACACAATTTCCGCTAGctagtgttgtgtgttgtgtggattaggcccgatg

gggataaggagcaagcaggtgcggcattatatgggtgggttcccaatagt

cagcggcagtagttagcagcaaagtataacttcatataactattgccttt

gaggaatcaaacttacgcatattgttgtcgtattaatttccagtggaagc

ttaatagtctcgggacttgttcttgtttaagattaattggcagaaataat

SIX8b locus

ggctaaaatacagtacgtacgtacgtacgtgtacaagaaattgcatcact

agaggcagtagttaagtacctaaaagcttaattagaaatcgagcaaatgg

ggttcgaatctactctttacagcaaaaccattgcaagtgcgggattcatg

gttggtaacacgggaaggtagcgtattgaagttcaggcttcacggagata

actatcgagtctttccgatattttctgaactgggtcgtatctaccgtata

tctaaccaacggatcagacgtgcggcaaggagaagtgtttccctttgtga

ggtaatatatgttcgtcgcttaggattaaactccagcgatagagtaatag

ttgggcagaacgcaggtttctcttcctttaagtacttaaagcacattgat

aatccatatattattaataaccctgttatagta**ta**ccgtgtggtgcaata mimp-IR1

agtttgaggatctttgaaggtcgtgttcatctgccgatttgatttatctg

ccggtct**ta**CAGTGGGATGCAAAAAGTATTCGCAGGTGTACAGGTAGGTC

TGATGCTCGGAAGCTAGTCAGGGGTAGCTTGTCAGGTCCTCAATACCTTA mimp4

TATTACACCTAAATCAGGGGGGTTACATAGTATCAAGTACCTAAGCTAAG

CTAAGCTACTAACTAGAGAAGCTAGGGCCGATCAACGCGACTTTCACACC

TGCGAATACTTTTTGCATCCCACTG**ta**tgtactagtatttaggttaaagc

ggcagaactacctttgacctgtattcttttagtgtttcggcatatagttt

aagctttcattcatat**ta**cagtggggagc**aataa**TGTCAGATATTAAGGT mimp-IR2 (part)

GAATTGTGTCTCTTCATTCTGTCGCAATCGAAGGACTATTGAGACCTTAT

ATCTTCGACTGGTTCCTTCTAGGACTTGAGTCCTAGAAAGTCCTAGATGA Han

AAGTCCTACGATCTAAGTTTAGGACTCCAAACTTTAATCCTGAACCTTAA

TCCTGAAGTTGAGGGTGAACCCTCCAATATTCAACA**aataa**gtttgagga mimp-IR2 (part)

tctctgaaggtaccgaagcagcccgtctagccggattagatccttgttac

atatctgtcggtcgtgttcatctgccgattttatttatctgccggtctta

tgtactagtatttaggttgaagcggcagaactacggatacttcagtctgg

attcaaaact**ta**CAGTGGGATGCAATAAGTTTGAATACCTTTTGAAGTAC < mimp-IR3 (part)

TGATACCCCCTGTCTAGCCTATTTGGAGTTCAGCCTGCTTCTAATCTGAG

GAAGAAGCTCGATCTAGCCAATCAGTATATACTTATTCGCCGTGCAACAA mimp1

CTGATATGCAGGTGGTAGCTGTCTCAAAGAGGCTAGCGGCAAAAGAAGAT

ACACGCTCGGTATTCAAACTTATTGCATCCTACTG**ta**ttgcatcccactg < mimp-IR3 (part)

**ta**atatgaatgaaagcttaaactatatgccgaaacactaaaagaatacag <

tacaagtcaaaggtagttctgccgctttaacctaaatactagtacataag <

accggcagataaataaaatcggcagatgaacacgaccgacagatatgtaa <

caaggatctaatccggctagacgggctgcttcggtaccttcaaagatcct < mimp-IR4

caaacttattgcaccccactg**TA**GATATCTACCTGATATAGCTAGTCAGG

CTTCTGACAGGGCTAGAGCGAAAAGGCAGATACTTCATCTTGGATTCAAA mimp3 (partial)

CTTATTGCACACCACTG**ta**cttcggcttatagtttaagcttttattttta

ccacagtaggagtagtcggcctagccagatccgattcttattataaatct

gccgaattcttctgccggtcttatatactagtatttaagttcaagcggca

gaattatctttaataatacaaccgacaggtatatggta**ta**ACGTGTTTGA

TAAGTGAGTGGGTCAGACAAGTAAGTGAGTCACTGCACTGTATAGTATTA

AAAAACCTACCCTATAGTATCTTTTTACTCTAAATAAAATAGATAAAAAT

TTAAAAAAAACATCTTTAAGAAAAGTATAATTAACCTTATTATAAAATAT mFot5

TTTTTTAAGATTTTTAGCTATTTTATTTTATATAGTAAAGCTTAATTATA

CAGTATAAAATACAGTATAAATAGGTTTTAAGGTTTATAGCTATATAGAA

TTTTTCTAAAAATCTTAAAAAATCTATATGATATATTTTAAGACTTATAT

ATAGGCTACTAAAATTACAGCCAATTTAGGCTTAGTTTGAAATTTTAAAG

AACTTTTTAAACCCCGGATTTAAGGTGGAAATAAGATTGACCCACTCACT

TGGATGACCCACTCACTTATCAAACACGT**ta**gttaaactttattttttaa

taaactttaaaatgtaccctgtaataacagagaatggagcttttaaatta

tgttataagggaattaaactctatactctacctgtactaaaagtaaagct

tttaagagagatttaagcggggaagtgggccttataagactagaaacata

tttaatgttagactctccctctattctctcgttcccggacatcctttcat

actgcgtcaagagtccgggtttacccacccttagttacgcctcttaaata

agtcttcgcgaccttataaaccctcctcaataccataaacggcaactATG

CATCCCTCACGCATGCTTTTACTCCTCCCCCTAGCCGTCTCTGTGGCTAC

TACTCATATTAAGCAGAGgtatgtctgattctcatgaatacgtccgtcac

acttatcagtacatcaagGGACGACCAAGCCGCAACTATTGAAGAAGCTA SIX8b

TTAATGAACCTCATTCTCTTGACGAGAGAGCCCTTGTTGAACGgtaagta

gcttgatatagattttagggttggtctaatttaaattacagCGACACCCG

TGGCATTTTACTCGCCTGCATAACGGGTGCTGGCACGGCGTTTCAGGCAT

ATACAGGATGCTATCTCTCCGCATTTCGTAATGACCCCCGCACTCTGGCG

GTAAGGGTGGACAGAACTTTTGGTGGAAGGACGTCGAACGTGCTTGTTAC

CTTGAGCGGGGGAGCGCTGGCACGGGCGGTAACTGAAGTGGTGCGAGTCT

TGCCGGGTGGGGTGGTGCACATTTTTACACAAGGTGCAAGTACTGTCTCG

TTTTCCAACAGTGGCACATAGgcagtcccatccgggtcagggttagcagc

tatgtctaaggatttcaataggggaaaagctgggacttggtgaaaattgc

cttcccttgaaaaaaaaagagactcttttccctgtagctaggtcaatcat

gggatgcacgtgatattatatacatcattctataccccgtgttggaggcg

SIX14 locus

tctctttatttctcaaatgctcatctttctattaagtcttataacatgag

cgtatgacatcctcttcttgtcatgaacgcgaggtttttgttcctgtagg

tgtcttcaatgcactcttgcttagccccaaacctggtaatagcctagttc

actagtataccgtgtggagccatcagctttgaggctttctgaaatgtccg

tgttcatctgccgatttgatttatctgccggtct**ta**CAGTGGGATGCAAA

AAGTATTCGCAGGTGTACAGGTAGGTCTGATGCTCGGAAGCTAGTCAGGG

GTAGCTTGTCAGGTCCTCAATACCTTATATTACACCTAAATCAGGGGGGT mimp4

TACATAGTATCAAGTACCTAAGCTAAGCTAAGCTAAGCTACTAACTAGAG

AAGCTAGGGCCGATCAACGCGACTTTCACACCTGCGAATACTTTTTGCAT

CCCACTG**ta**tgtactagtatttaggttaaagcggcagaactacctttgac

ctgtattcttttagtgtttcggcatatagtttaagctttcattcatat**ta**

cagtggggagc**aataa**gtttgaggatctctgaaggtaccgaagcagcccg mimp-IR2

tctagccggattagatccttgttacatatctgtcggtcgtgttcatctgc

cgattttatttatctgccggtcttatgtactggtatttaggttaaagcgg

cagaactacctttgacctgtattcttttagtgtttcggcatgtagtataa

gctttcattcatacag**ta**CAGTGGGATGCAATAAGTTTGAATACCTTCTA

AAGTACTGATACCCCCTGTCTAGCTTATTTGATGTTCAGCCTGCTTCTAA

TCTAAGGAAGAAGCTCGATCTAGCCAATCACTATATTCTTATTAGCCGTG mimp1

CAAAAGCTGATATGTACGTGGTAGCTGGCTTAAAGAAGCTAGCAGTAAAA

GAAGATACACGCCCGGTATTCAAACTTATTGCATCCCACTG**ta**gcctttg

tttttgaagttgattgagatgacatcccctggattgattccgcccaacgg

atcatagttaataatatgatgcgcggcgacatttacagtcccagcgtcga

aagccgagccttgttcgcgaaatgttatacaattcccagccgccaatcta

acacataaagaccccgcacctgacagcacattatgtgagacggcgaccta

gaacaa**ta**CAGTGGGATGCAAAAAGTATTCGCAGGTGTGTAAGCCGCCCT

AGCTTCTCTACTTAGTAGATAAGCTTAGCTTAGGTACTCGATACTAGGTT

ACCCCCTTGATTTAATTGTAATATAAGGTATTGAGGACCTGACAAGCTAC mimp4

CCCTGACTAGCTTCCGAGCATCAGACCTACCTGCACACCTGCGAATACTT

TTTGCACCCCACTG**ta**tagagttccaaaagtaccgtactttgtaggagcg

ttttattactctcattgtatgactagtcctgtctgatgccagcttccttg

ttaggttaaagtcagtggcggcaggggcaattaattactacgtagtagag

gcatacgtaatcccttaaggtcccaccacctaaccaaaaaatatataaag

tgcgactggacttctgccaaccgtaaatcagaattgccttagatttatac

tgtcaccactatcttgccacctATGCATACCGAGTATTTATTTCTACTTT

TGATCCCAATGGGGGCTGTGTCGCAGCGTATCCTCGGCTGCCGCATGCCG SIX14

AACGGATCCTTAAACCCAAGTCCCAATATATGTAACCAAGCAGGAGGTAG

TTTCAGGTCGGGATCGgtaagggcgccattagcgatatgacgcattcgga

actaacaggaagtaagCGAGGGTGCTGCACCCGGAACACCCGGGACGGTC

CCGTTGTCACTGAATCCCGATTCATCAGCGGATGTAATAAAAATGGTGGT

TTTGTCAGTTCCAAAGAGATTCTAGCCACCAGCTGTTAAggttcgcttag

gaatgtggatgggggtcactttaacccgaggtgaggatacttaagtgtgt

tcgagcctagtttcattcccgtacaccctgtggtgtcttaattattctgg

atggtcagtgatagtgatggctgtttct
