# Supplementary material for: MITEs in the promoters of effector genes allow prediction of novel virulence genes in Fusarium oxysporum
Source: BMC Genomics. 2013 Feb 22;14:119. doi: 10.1186/1471-2164-14-119 (PMC3599309; doi:10.1186/1471-2164-14-119)
Supplement: Additional file 5 — Newly identified TEs of Fol. [file 1471-2164-14-119-S5.doc]

>Nht2-like retrotransposon type 1

TGTCGCAGGCCGGGCATGGTCTCAATGATCCTCAGGAACAACAGCCCTTA

GTAATCAAGCTGGGTGATTTAAAGTAAAACCTGTGGGTTGGAATACCCCA

CACAGAAGACAATCAATCAACTCACTACTGCTGCTCATCATACTCACTCT

AACCCTTTAAGCACGCTCAGTGCGACAGTTATGAGCCCGGAGACTCTTCC

GCTACGTCGCACGCGATATTTATCACCATTACCAGCTTTCCTGTCAACCT

GGACGCAATGAAATCTTTCTTCCGACAGGAAATTCTCTTCAGAGAAACCG

GAACGAAGTTGGACCGGTTGCATTATGCTTACTGCGGGGTTCATCACCAG

CCGCGAAAGCAGCAACTACAGAGAAGACTCAGAAAAACTGATATTTTCAT

AATGATTTCAATTAAAAAGGAAGCTAACGCATTAAATAGAAGTCAACTAA

AAAGTGAAGTTAACATTGCCTTTACCAAAAATCATCAAACACAAGATGTC

GATCAAAGACCATAACCTCGCTATGCTCAACGCCCGTGAAGACTGGACGA

ACTGGATTAATTCTATCGAGGACCTTGCTGTGCGTAATGATGTCTGGAAC

TATTGTGATCTAGAAGGGATCGAAAATCTGGTCTTTACTGCGACCAAACC

ATCAGACTCAGCGAGCAAGGATACGATCCAGAAATACCACAGCCTCCAGG

CTATCTACGAGAGCGAGAAGAAGAAATATGACAAGGTCTCTGATCGCATC

GACCTCACGGTGTGTCAGGAGTTCAAGCAACACTACCTCGGAATCCATGA

CGTCCGCGGCAAACTCATCGCGCTGGCTGATAGCATCCAACCCACCGCGA

AAGATCAGAAGCAGAATGTCAGGACCGAGTTCGAATCGCTTAAGAAAGGC

CCTGGCAACACTAGTCTTGACAAATGGCTTAGCCGCTGGCCTGCTCTCGT

CAGCAACGCCAAGCGTTACAAGATCGAGAATCTCAGCGAGTCACAGATTT

GTGATGCGTTCATTGAAGCATCTCGTGAAGTCAACCCACCGTTCTACAAC

TACATGAAGTCCAAAGAAGCTCAGGTTGAGAACGAGAAGAACCTCATCAA

GGAGGCTGCAAAAGCCATGGAGAAGATCTCAAACGCGTTTCTCACCGCGC

TTAACGAGATTAATCCTGACCATGCCAGTAACGGCTCTATCACCGTTGCA

AGCTCGGCTGATTCTGAAGAGGACGAAAATGATCAAAATGATGATGCCAC

TATTGTTCAGAAAGCTCAGAATACAACGAACAGAGCTCTCAGGGCCTTCC

GCAAGCTCAACCCAACCCTCTCCGAGAAGAAGATCACGATCGGATTCTGT

ATTAGGCAATTCAGAACAATGGCACCACCGAAGGAGAACGCTACTAGGGG

ACGAGCAGCCCATGCCACATTCCAAGATAGGAAGCATGGCAATGGCTGGG

ACTCGCAAAACGAGGAGAGCCAGCCCCAGCAGAAAAGACAGCGAACACAA

CCTTCATCTTCGAGCACAAACCCTCAACAGTGCGTATGTGGCATGTCTCA

CAAGTATGCTGACTGCTACTACCTAAATCCTTCGAAGGCACCAGAAGGAT

GGACACCAGCCATCCAAGTCCAATCGAAAGTCATCACTGCAGTCAAAGGG

AGCAGGAGGCTTCGGACAAACATCGAAAAGAACTTCAGGAGAAACAAGAT

CAACCTACCTAAGTTCTGGCAGTCTGACAGTACGGAGAATCAACACAAGA

CTCCGACAGAAGACGAGACTTCAAACACTGCTGCCGCGTTACCACGCAAA

TCAAGAGCTGCATTTGTCACGTCAAAGTTCGCACTCAGCACAACCGCAAA

AGACGAGTACGGCGACTGCTTTCGACTCGACAACTGCGCGGATACGCATG

TCTGCAATGACCTATCTCGCTTCACTAAATACAAGCCTCTGGACAATGAA

GTCATCGAGTTTGGCGACTCAGGCACATACATCACGGGTGTCGGCAATGT

CACAGTCCATGTTGATACCCCATCGGGTCCAGGCTTAATCCAGATCGAGA

ATGTTGCCCATGTTCCCGGATTCCACTGGAACCTCATCAACACGCACTCT

CTCGAACAACAAGGCCTCTATTTTAATACTAGGACCTGTTGGATGGAGTA

TTCGGATGGATCAAATGCCTTCAAGGCAACAAAGTATGGTGCTTTCCGAG

TGGTCGAACCTTGCATGAAGGACGCTGTCTTCAAGGCAAAGTCGCACCAA

GAGGCTGTGAAGTCATTTGCAATGGCGACCAAGTCGAGAACACCTCAAGT

GGCAATAGCATCTATGGATATCTGGCACGCAAGGCTTGGCCATATCCGTA

AGGAAGCTCTCGAACACGTGCCTCAAGTAGTCGAAGGAGTCGCACTTGGC

ACTCACGACTTTGAACGAAAATCTGAGCTTTGTCCCGAATGCCAACTCGG

ACAGGCGCACCAGCAAATCTCACGTGTACCAACCTGGAGGGGAACCTACC

CTTTCGAAAGGATACATCTCGACCTTGTCGATATGGAGGAAGCCTTCAAC

GCCGACTCCTGGGTCGCTCACTTTTATTGCGACCACTCGGCTTACCACGT

GTCCTTCAATCTCCCGAACAAGGCTCAAGAGGAACTCCTGTCGGCTACGC

AAGAGTTTCTCGCCATTACAAACGACAACTGGGGTTTCACTACGCGGTAC

ATCCGATCAGATGGCGAGAAAGGTCTGGGCAAGAAATGGAAGCATTTCAT

TACAATGAAAGGAATCATCTTCAATCCATCTCCACCAGATACGCCAGATC

AGAACGGTCCTGCCGAACGTTCTGGGGGGGTGATCATGACTATCGCTCGC

AAGCTTCGAATACAGGGAAATCTACCTCAGAAACTCTGGCCCTACATCGT

TGCTCATGCTACCCGCCTCCTCAATCGCATCCCGGTTCAACGAAAACAAT

GGCGAACTCCGTTTGAGATGGTTCATGGCAGAAAGCCGAACCTCTCTCAT

CTCAAAATCATTGGCTCTCTCGCCTATGTATTGATCAAGAACAAGAAAGC

TCGTCCGGCCAGAGCGAAACTACAAGAAAACGCGCTCACGGGATGGCTTG

TCGGATTCGACGCAACTAATATCCACAAAGTTTGGATTCCACACCTCGAT

CGTGTTATTGTATCTCGTGATGTTCAAATTGACGAGAAAGTCATGTACGA

TCCACAGCTTGCCACAACTCGTCCTGAATCAGGACAGGCTCTCGCCATTA

CGGTCAACGAAGTTGATCTGGACGAAGAAGATGTTGAGCCATTGCCTATC

ACGGAGGATATAGCAACATCAGTTCCTGTTTCGATCCAAACAGAAGTAGA

GCCACCTCCACTAGGGTTGCTTCTCACACCACAAAGCTCACCTGAACGAG

CAACAGCAGGGGAAATACAGGTTGAACAGCCAGACGTGTTGCGGCCCCCG

AATCAAGCAGCTCTCCATCAAGCATCATCGCATCCTCGGACAAATATGTC

AGGAGAAGAGGCACCCAGAAAGGAAGGAACAACGGACGCCGAGGTGCTCC

AGAACCTTCGGACAACTTCTGGGAGAAACATCAAACTGTCTCTGAGAGGT

CAGGATGCAATCGAAACATTCAAACCGCGCTCCACCCTACATCGAGTTCA

GAAGCGAGCACGAAGACAGGCATACACCCTACGACTGGAACGCGCGAAGC

TGGGACATCAGATCGCCCACGCCTTCGCAGCTGCACGGTCAATCCGAATC

CATCGGAGGGACCTGCTACCACCACCTGAATTCTGGCATCAACTAAAACG

GCACCCTGAGAGAGAAGGCTTCAAACGAGCTGCAGGCGCCGAGATCAACT

CACTCAAGGAGAAGAAATCCTTCCAACTAGTTGATTGTCCAGAAGGCAAA

CAGATACTGCCGCTCAAATGGGTCTTTACCTACAAACTGGATGATACAGG

ACATCTCATTCGTCATAAAGCCCGCATCTGTGTCAGAGGTGATCTCCAAC

ACCACTCTGGTGAAGACATCTACGCAGCAACCGGTGCTTATCGAAGCTTC

CGGATTCTGATGGCTCTCGTCTGTGCGTTTGGGCTGATATGTCACCAAGT

TGACTTCAAGAACGCCTTCGTCAACGCTGAAATGGACGAGGAGGTCTACA

CCACATGCCCACCGGGATATGGTCAATCAGGAAAGGTCTGGAGACTGCTC

AAAGCGCTCTATGGTCTGCGCAAGTCACCAAAACTGTGGTACCATGAACT

TGTACGGTTCCTCAAAGACCTGGGATTTGAACAATGCCCAGATGAACCAT

GCATTCTCATTAACAACGAGACTCATCTTGTCTTGTTTCTTTACGTCGAT

GATCTTCTCATCATCGCACAATCTGACTACCTCCAACAAGTCAACGAATT

CAAAGCAGCAGTAAACGGCAAATACGGGATTAAGGATTTGGGAGAAGCAA

TCACCTTCCTCAACATCAGGATCCTTCGAGATACCAAGGCCAAGAAGCTC

TGGATCTGCCAGGATGGATATATCGACAAACTTTGTAACAAGTTCGGGAT

CGACAAATCAATGAGAACGACAACTCCTCTCATTCCGTCGTACCGTCCCC

AACCTTTCGAAGGACAGGCCACTATTCGACAGATAACAGAGATGCAAGAA

AAGGTAGGATCAGTTCTGTATGCGGCAGTAGTAACAAGACCAGATGTCTC

CTATGCAGCTAGCCAGTTAAGCCAATTCGCTATGAACCCTTCACCAGAGC

ACCTGCGGTATGCGAACAGAGTTCTCTCATATCTCCAGACCACACGGTAC

TATGCCATCGAGTTCTCGGGCTCTGTGGATAAGGCCACAGCAGTAGAGAC

AGGCGATGATGAAGTACTACAGCAGTCAAGCGACGCATCGTTCGCGGATG

ATCCAGAGACTCGAAGATCCACCCAGGGCTATCTCATGAAGTTGTTTAGC

GGGGCAATCATGTGGCAGTCTTCAAAACAGAAGACTGTAACCACGTCAAC

GACTGAAGCGGAACTGTTATCGCTGTCTCATACAGCGAGGGAGACAATAG

CTCTATACCGACTGTTCGGTCAGATATAATTCGATCCAGAACACCAACCA

CGCATCCTCTGTGACAATCAACAAACAGTGGGACTGGTCCAGAAAGAGAG

ACCACAACTAACATCAAAGTTAAGACACGTCGACATCCACAATTTTTGGC

TAAGACAGATCCATCGAGACGGCAAAATTACAGTTCAATGGGTTCCAACA

ACAGACATGCCTGCAGATGGCTTCACAAAACCTCTTTCAGCAGAAAAGCA

TTCTCACTTCGTGAAACAATTGGGCCTGGTTGATATCTCACTGCGGATTG

ATCCAGAACACGTTTCAGAGGAGGATATCGATCAGGATGATATGCAGATC

TCATCTGATACAGACTGAAATCTGGGGGGGTGTGTCGCAGGCCGGGCATG

GTCTCAATGATCCTCAGGAACAACAGCCCTTAGTAATCAAGCTGGGTGAT

TTAAAGTAAAACCTGTGGGTTGGAATACCCCACACAGAAGACAATCAATC

AACTCACTACTGCTGCTCATCATACTCACTCTAACCCTTTAAGCACGCTC

AGTGCGACA

>Nht2-like retrotransposon type 2

TGTCGCAGGGTAGGCATTAGTAATCTAGCTGTATGCTTTAAGTAACTCTG

CGGGATGGAATACCCCGCACAAGAGACAACTTAAGACATCAATCAACACA

TTTTGCTGCTGCTCATCATATTCACTCTACCTTTAAGCACGCTTAGTGCG

ACAGTTATGAGCCCGGAGACTCTTTCATTGCCTCTCTCCATCATGATCAG

CATCTTCTCTGCCGCAAGCCAAGTCCATCGCCATCAGCGTCTTCTGCTAT

CAACTTCGTCGTAATGAGAGCTTTCTCTCAAATTGAAAATCTCCTCAGGG

GAATCGGCACGAAGTTGAGCCGACTGACGCAATGCTGGTTTCCGGTTCAT

AGTCGGATGTATCAGATGAAGCGATATGAAACAGCAACTTGAAACCCGCA

AAAGATTTTAACAGAGAAGACTCCTAAAATCTAACAATCATGATCTCTTT

AATCAGCAAGAAGCTAACACATTAATCAGAAGTCAATTCAAAAAGGACGC

TATCCTTTTCTTGACTAAATGTCACAAAATGTCGATCAAAGATCACAATC

TCACCTTGCTTAACTCCCGCGAGGACTGGACCAACTGGATCAACTCTATT

GAGGATCTTGCTGTGCGCAATGACGTCTGGAGCTACTGTGATCCAGAAGG

GATTGAAAACTTAGTCTTAACGGCGACCAAACCATCGGACTCAGCAAGCA

AGGACACGATCCAGAAATATCACAGCCTCCAGGCTATCTATGATAGCGAA

AGGAAGAAATACGACAAGGTCTCTGATCGCATTGACCTCACAGTGTGTCA

GGAGTTCAAGCAGCACTACCTCGGAATCCATGATGTCCGCGGCAAACTTA

TTGCGCTGGCTGATAGCATCCAACCCACTGCGAAAGACCAGAAGCAGAAT

GTTAGGACTGAGTTTGAAAAGCTTAAGAAGGGCCTTAGCAACACCAGTCT

CGATAAATGGCTCAGCCGCTGGCCTGCTCTCGTTAACAACGCTAAACGTT

ACAAGATAGAGAACCTCAGCGAGTCGCAGATCTGTGATGCATTCATTGAA

TCATCTCGCGAAGTAAACCCACCGTTCTACAACTACATGAAGTCCAAGGA

AGCTCAGGTTGAAAGCGAGAAAAACCTCGTCAAGGAGGCTGCAAAAACCA

TGGAGAAGATCTCAAATGCGTTTCTCACTGCGCTCAACGAGATTAATCCC

GACCATGCCAGTAGCGGTTCCGCCACTGTCGCAAGTTTGACTGATTCTGA

AGAGGAAGAAAACGGTGAAGATGACGATGTCACCATTGTTCAGAAAGCTC

AGAATACAGTAAATAGAGCACTCAAGACCTTCCGCAAACTCAGTCCAACC

CTCTCCGAGAAGAAGATTACTATCGGATTCTGTATTAAGCAGTTCAGGAC

AATGGCACCACCAAAGGAAAAAGCTATTAGAGGACGAGCAGCCCACGCCA

CTCTTCAAGGAAGAAAGCATAGCAATGATTCAGATTCGCAGAACGAAGAG

GATCAGCCGCAACAGAAAAGACAGCGGACGGGGCATCCATCCTCGAACAT

GGGTTTCCGAAATTGCGTCTGCGGCATGTCCCACAAGTACACTGAGTGTT

ATTATCTTAATCCTTCGGGTGCGCCAGAGGGATGGACACCAATCGTTCAA

ATACAGTCGAAAATCATCACTACAGTCAAGGGAAGCAAACGCCTTAAAGA

AAACATCGAAAGGAACCTCAAGAGAAGCAATATTGACTTGCCTAAGTTCT

GGCCGTCAGATATTACAAAGAATCAACCCAAGCATCTAGCAGAGGACGAG

ACTTCAAGCACAGATAAATTTCTATCCAAATCAAGAGCCGCATTTGTTAC

GTCAAGGTTTGCGTTTGGCACAACCGCAAAAGACGAGTACGGTGATTGTT

TCCGACTTGACAATTGTGCGGATACACATGTCTGCAATGACCTGTCTCGC

TTCACTAATTACACACCACTCGATGATGAAACCATAGAGTTTGGCAACTC

AGGCACGCACCTCACAGGCATAGGAAATGTCACGGTCCATGTCGACACTC

CAACAGGCGCAGGTCTAATTCAACTCGAGAACGTTGCTTATGTCCCTGGA

TTCCATTGGAACCTCATCAATACGCACTCTCTCGAACAACAAGGACTTTA

TTTCAATACGAGGACTTGTTGGATGGAATATTCAGATGGATCAAATGCCT

TTAAAGTAACTAAACATGGTGCTTTTCGAGTCGCCGAATCCCACATCAAG

GACATTATATTCAGGGCTCAATCTCATAAAGAGGCAGCGCAATCCTTTGC

AATGGCTACTAAATCGAGAACGCCTCAAGTTGCGACAGCTTCTATGGATA

CCTGGCATGCAAGGCTAGGCCATATTCGAAAGGAGGCTCTTGAACACATG

CCTCAAGCAGTCGAGGGAGTCGCACTCGGGACTCGCAACTTCGAACGAAA

GTCGGAACTCTGTCCTGAATGTCAACTCGGACAAGCGTATCAACAAATCT

CCCGCATACCACCGTGGAGAGGATCTTACCCCTTCGAGAAGATACACCTT

GATCTTATCGACATGGAGGAAGCATTCAACGCTGACTCCTGGGTCGCTCA

TTTCTATTGCGACTACTCAGCTTACCATATCACCTTTAACCTCCCAAACA

AGAATCAAGATGAACTTCTTTCTATAACACAAGAGTTTCTAGCCATTACA

AATGACAACTGGGGTTTCACTACGCGATACATCCAATCGGATGGCGAAAA

GGGTCTGGGAAAGAGATGGAAGTATTTCATTGCAATGAATGGAATTACAT

TCAACTCATCTCCACCAGATACGCCAGATCAGAACGGTCTTGCCGAACGT

TCTGGGGGGGTGATCATAGCTATCGCTCGCAAGCTTCGAATACAGGGAAA

TCTACCTCAGAAACTCTGGCCCTACATCGTTGCTCATGCTACTCGCCTCC

TCAATCGCATCCCAGTTCAACGAAAACAATGGCAAACTCCATTTGAAATG

GTTCATGGCCGAAAGCCCAACCTCTCTAATCTCAAAATCATTGGTTCGCT

TGCCTACGTCTTAATCAAGAACAAGAAGGCCCGACCAGCCAAAGCGAAGT

TACAAGAAAATGCAGTCATGGGATGGCTTGTCGGGTTGGACGCAACCAAT

ATCTATAAAGTTTGGGTTCCACACCTTGATCGTGTAATTGTATCTCGGGA

TGTTCAAGTTGATGAGAAAGTCATGTACGATCCACAGCTTGCCACAACTC

TTCCCGAATCGGGACAGACTCTCGCGATCACGATCAATGAAGTTGATCTC

GAAGAAGAGGATATCGAGCCATTGTCTATCATGGAGAATACAGCAACTTC

GGTTCCTGTTTCGATACAACCGGAAGCAGAACTATCCCGACCAGGATTGC

TTCTTACACCACAAATCTCACCTGAACGATCAATAGCAGGAGAGATACAG

GTTGCACCACCCGTAGATGAACCGCCAACATTGCCGCGGCCCACGAGTCC

AGTAATTTTCCACCAAGCATCACCACGTCCTCAGACAACAATACAACTAG

GATCACCCAGAAAGAAAAAGATAACGGACGCTTCAGTGCTCCAGAATCTC

AGAACAACTTCTGGAAGGATTATCAAATTGTCACAGAAAGGACAAGATGC

AATCGAAACATTCAAACCACGCCTTACTCGGCAACAAATCCATAAGCAAG

CGCGAAGACACGCTCATGCTCTACGATTGGAACGCGCTAAGCTGGGACAG

CAACTCGCCCACGCTTTCGCATCAGCACGTTCAATACGAACCCAACGAAA

GGACTTGCCTCCACCTCCTGACTTTTGGCATCAATTGAAATGGCATCCTG

AGAGACAAGGCTTCAAACGAGCCTCAGACGCCGAGATCAAGTCACTCAAG

GAGAAAGGGACATTCGAGCTAGTCGATTACCCTGAAGGCAAACAAGTTCT

ACCACTCAAATGGGTCTTTACCTACAAACTGGACGATGCAGGTTACCTCA

TTCGTCACAAAGCTCGCATCTGTGTCAGAGGCGATCTCCAACACCACTCT

GGTGAAGACATCTACGCAGCAACCGGTGCTTATCGAAGCTTCCGGATTCT

GATGGCTCTCATCTGTGCGTTTGGGCTGATATGTCACCAAGTTGACTTCA

AGAACGCCTTCGTTAACGCTGAAATGGACGAGGAGATCTACACCACATGC

CCACCGGGATATGGTCAATCAGGAAAGGTCTGGAGACTGCTCAAAGCGCT

CTACGGTCTGCGTAAGTCACCAAAGCTCTGGTTCAATGAGCTTGCATCGT

TTCTTAAAGATCTGGGATTTCAACATTGCCCAGATGAACCCTGTATCCTC

ATTAATAATGAGACTCAACTCATTCTCTTTCTATACGTAGATGATCTTCT

CATCATCGCGCAACCTGAATGCCTTCAACAAGTCAACAATTTCAAAGCAG

CAGTACACAGCAAATACGAGATCAAAGATTTGGGCGAAGCAATCTCCTTC

CTCAACATCAGAATCCTCCGAGACCTCAATGCTAAGAAGCTTTGGATCTG

CCAAGATGGATATATCAACAAACTCGGTGTCAAGTTCGGGATCGATCAGT

CAATGAGAACGGCAACGCCTCTCACTTCATCCTATCGTCCCCAATCTTTC

GAAGGACAAGCCACTATACAACAGATCACAGAGATGCAAGAAAAGGTAGG

ATCGATTCTGTACGCAGCAGTAGTATCAAGACCAGATATCTCTTATGCAG

CTAGCCAGTTAAGCCAGCACACAATGAACCCTTCACCTGAACACCTGCGG

TATGCAAACCGAGTTCTCTCATATCTCCAGACCACACAGTACTATGCCAT

CGAGTTCTCAGGCTCTGTAGATAATGCTACAGAGGTAGAGCCAGGCGATG

ATGAAGTACTACAGCAGTCAAGCGACGCATCGTTCGCGGATGATCCAGAG

ACTCGAAAATCCACCCAGGGCTATCTCATGAAGTTGTTTAGCGGGGCGAT

CATGTGGCAGTCTTCGAAGCAGAAGACTGTAACCACGTCAACGACTGAAG

CGGAACTGTTATCGCTGTCTCACACAGCGAGAGAGACTATAGCTCTATAC

CGACTGTTCGAACAGATACAGTTCGATCCACAACACCAACCACGCATCCT

CTGTGACAATCAACAAACAGTAGGACTGGTCCAGAAAGAGCGACCACAAC

TAACATCAAAGTTAAAACATATCGATATCCACAACTTTTGGCTAAGACAG

ATCCATAAAGACGGCAAAATTACAGTTCAATGGGTTTCTACAACAGACAT

GCCTGCAGATGGCTTCACAAAACCCCTTTCAGCAGAAAAGCATTTTCATT

TCGTGAAACAATTAGGTCTGGTTGATATCTCATCGCGGATTGATCCAGAA

TACGTTTCAGGAGAGGATATTGATCAAGATGATATACAGATCTCATCTGA

TACAGAGTGAAATCTGAGGGGGTGTGTCGCAGGGTAGGCATTAGTAATCT

AGCTGTATGCTTTAAGTAACTCTGCGGGATGGAATACCCCGCACAAGAGA

CAACTTAAGACATCAATCAACACATTTTGCTGCTGCTCATCATATTCACT

CTACCTTTAAGCACGCTTAGTGCGACA

>Nht2-like retrotransposon type 3

TGTCAGACATTACGTGTATTCGGTTCATTGATGTGAATTGGTGTATTCAT

GTTGTGTTCTGTTGAAATCTGCCCCTTAGAAGTTTTACCATTTCGGCTGT

AATCTTAAAGTATCCCTGCTTGCACGTGAGGCCCCAGCACCTGTTCAACA

CACCCCCCCAGACTGGCCATCGTGGTTCAGTCTGTGCAGTCCGTATTCCT

ATTGTCAGGTTGGCCATCCTTCCGATCCCTTATTCGGCTCTTCCTCCATC

GAGATGTTCCCCTGTTCTGACAAAGCCCTCCGCAGTGAGTTCCTTGGTCC

TTCTCTCTTGAATCCTGTCCTTGATATCAACTAGGCCAACCTGTGTGACG

AACTTCTGGAACTGCTGAGCGGGTAATGCCTTCGTGAGGCCGTCCGCAAT

CATCTCGGTGGTGGGAGTATGCCGGACCGAGATCCGGTTAGCGAGTGCTT

CCTGGCGTAGCCAGTGGTTATGGATGTCGACGTGTCGAAGTTTGGTCTGC

AGGAGCGCCACGTCGGAATTAATAAGCCGAATGGTCTGCGCGTTATCGCA

CTGAATGATAATGCTTCTGTTGTCAAGGGTCACGCCGAGCTCGGTGATTA

ACCTGGAGATGAACATGGACTCCTTCGCAGCCTGCGCAAGTGCCAGCAAC

TCCGCCTCTGTTGTAGAGGTAGTCACGGTGTCCTGCTTGTTGGCGCGCCA

TCCAATAGTCCCTCCAAATAGCTTCATCACGTAGGCTTGTGAGCTCTTCC

GATCGATTGTGTTGTCGGCAAAGGAAGCGTCACTGTACACATCAAAAGTG

TCTGTTCCGCCGAGTTGTAGTGCGAGTGCCCTGGTGTTTTGCAGATACCG

GAATAGGTGGTCCAAGGCCTCATGGTGATCATCGCTCGGGTTCATGTTGA

ATCGACAAAGTCGAGAAACAGCAAAGGCAATATCAGGTCGAGTGATTACT

GCCAGGTAGAGCACTGATCCGGTCTTGCGTTGATACTGCTGTATTGACGC

TGTTGATGCCCGCTCTTCGTTCGGAAGAAGTTCTTTTGTGGTCATTGGAG

TGAGGGAAGGCTTTCCCCGTTGAATCCTAAAATGACTAGTGATCTTATCC

ACGAATGACGATTGCGACAGCCAAATCAGACGTTGGTTTCGGTCGCGAAT

AATCTCAATTCCAAGAAACCACTGTAGCGTATCTCCTCCAGAGAGTTGGT

ATTTTTCTCGAAGCTTGCCTATTGTCCATTCGACCGATTTTTGATTGGTC

TTCTCGTAGGCAACAATGAGGTCGTCCACATAGAAGAATATCAATATTCC

TCCTTTTCGAGAGACAGCATGGCTCGTGAGGTACAGACTTGAACCCAATG

TTCGTAAGGGTGGTGGTTAGTTCCTTTTGCCAAAGTAATGGTGACTGGCG

CAAACCGTAGAGTGCTTTCTGAAGTTTGAGAATCAGGCCAGGCTTTCGGT

AGCCTCCGGGCATCCGCATGTAGACGTCCTGCTTCAGTTCAGCATTGACA

AAGGCGTTGACGACATCATACTGGAGAAGTTCTAAATCAAAGCGAGCAGC

TATAGCCATGAGTGTTCGGAAAGATCTGCCGGCAAGTGTGGATGCATAGG

TATCCTCGTGTGTCGACTTGGCCTGTTGATCGCCTCGTACAACGAGCCGG

GCCTTGCACTTGGTGAACCATCCGTGCTTGTCGAACTTGTACACATAAAC

CCACATGCAGTCCAGGAGTTCCTTGCCTGCGGCTTCTTCTCTCTGAACCT

CACGCCAGGAGCGCATTTCTTTATGACTTTCAAGATGAGTCTTTTCTGCC

TCGCGGAATAGGTCACCTAGTGGATGGTTCTTGAGGTCTTTATGCCTTTT

TGGCAATATTGGCATGTCTCGCCGGTGTATTCGGCGTTGGCCGTCACCTA

AGTCTGTCAGCTTTGCAGATTGTGTTTTGCGATTGGGAGAATCCATCAGC

TTAGTAGATTTCCCATGGCTTCCCGTAGACTGAGGTTCTCTGCTATCAGG

GTGGCAGCTGGCCGAGCTCGCCTGCTTCTCAAGCTTCCGCTTACTCACCA

CCTTGCCATTCCAGGTCCCTTTCACAGATGCAAGTCGACCGGCATTAAAT

GCGGCCTCCCAGTAACTGCTCGGAGTTGAAATGACACGAGAGGCTCCATT

TGCGCCGTCTCGTCCCGGGGAAGTGCCAGCGCTAGGTGGGACATGCACCA

TCTTGGCAGCTCGTAACTGAGGGGGCTTTTCCCCCTCCTTCCTCGCGGGA

GGTTCTCTGATAGTAGCGACCAGAAGGGCAGCAGGCCGTAGAGGGCAAGT

CTCCGGGGTCAGGTAGGGAGTGAATCGAGCGGTCGTGTAGGGGTGATCAG

TCCCGTCAGACTCTTCTCGATTGCGGTCTCCTACGACTAGTGTCCTGCCA

GTCTCACCCTGCGTAATCGTGGGATCACGTTCAAACCCTCTATGTGGATA

ACTGGCAGCCTCCCGCTCAAACGCTAGTCCTTCAGGCAAGGCGCTTAATT

CCTCGTCTTCTTCTTGGGTAGAAGCATCCAGATTGATGCTAGATCCCTGT

GGGACCTCAGCATCAGTGAGATGCTGAATAAGTTCTTCCTGGTTTAGTTC

TTTTAAATCATCCTTGAGGTGTTGCACATCCCCAGAGAACGTCTCCTTCT

CGTTGAAGATCACGTCTCTAGTGGAGATGACCTTGTTGGTCAACGGATTC

CAGACCCTGTAGATGTTTGTCGAGTTATACCCGACCAGGTATCCTATCCA

CCCTCGGGGATTGAGTCGTTGTCGCCGATTCTTCTTTTTCAAAGCATCGG

TCGTCATCGCGAAGGCTTTACAGCCATACACGCGTAGGTGTGTCTGATCG

GGCTTACGCTCCGTGACAACCACGCCGTCCCTAAAGGCAAAGTAAGTGTA

GAAACGCTCATATGGCGTCTTCCAATTGTAGATATACTTGGGTGTCCTAT

TGTATAGGTATACGGCAGCACGCCAGATCTCAACCCAAAGGAACGAAGGC

AGCTTTGCTCCAGCTCTCATCGCGCGAGCTTTCTCCTTGATCACACCCCC

CGAGCGCTCTGCGCCGCCATTTTGACTTTGGGCATATGGCGCGGAGGGCT

CTAGGCGCATCCCGTTTTTGGTACTGAGGAAGTCACTTACCTGGTGACTA

TCCGGGATCTCGTTATCACACTCGATGACTTCGGGCCTACACTGGAGTTG

TCGCTCTAGTGTTCTGAGTAGATGCTCAAGTGCACCGATAAGCGTTGGTG

CAGTTCGATCGGGAAGATAATAGTCCCAGATATAGCCAGAGTAACGGTCG

GTGACCAGCATAGCTGAAGTGTAGCCTGGATCCCCACTCATGTCGTGGAA

ATCAATAGCGAGTCGGATTCCAGCCTTCTCAGGTGGAACTCGTCTTTGTC

GACGGACTTGGCGCTTCACCTTCGCCATTCCACAAGCATCACACTCAACC

GTGGAGATCCCTCTGATCTTCACCCCCCGACATTGGCCCACGAGGTGCTT

GAGTGCTTCAGGTCCTGGGTGTCCAAGTCGAAGGTGCCAAAAGGTTTCGT

TTCCACTGCGAGGAGGACGCTTACTCCACGAGTCATACTGTTTCCGTCTG

GCTAGGAACGCTGCTGCTGTTGTCGATTTGTCGATGTACTCCAGTACGTA

CTGGTCGAAATGGTCCGTCAATGTGCAGAGGATTGAATCATCTTTCCTGC

GGATTTGGTTGTTGTTCCCTTTGGTATCCCAGTAGTAGCCCCTTTTCCGC

AACTGGCGGTATGACACAATATTGCATGCAAAGTTTATGCAATGAGCTAC

ATCTCGCAATCTGAGAAGAGAGGGTCCTTGAGGTCCTTGAACGCGAATAT

CCACGTTCCCGTACCCGTGAATGGCCACTGGTGACTCCCCTGCGTAGACG

AAGTCTCCAGACGGTGCCGGTCGGTAGTTGAGGAACCTGCATACTTCATT

GAAGATATGGATTGTCGATCCAGAGTCAAGGATGGCAGAATGTCTAAGAG

GGTAGGGGGCACAGTTGAAGGCTGCCATAAGGTTGTGCATCACTAGGTAG

GTTGCCTTGACGTGTGCCATACCCCGCCCAAGTCACTCGTCCTGAGTGGT

TCTGTCTCTACTTTTGGACAATCGTTTAACTTCCTCCACCAAACCAGTAT

CGTTCTTGAGACGGTCATTTATTAATCGTTGTATCGCTTGGTTGGGCTCC

CATCCTTCGGCTTTCTGGGCTGGGAATAAGTACCAGCAGCCTCTCCAGTC

ATGGAAACCTAAACATCCTCGACAAGTAGGTCCATCCGGGTTCGGATTGA

CGGTCGTCTCTGCTCGTTTTCGTTTTGTTGATACTCCTCTTCCGCCGCCT

CGTGCTCTACGCCCGCGGCCTTGACTTCGTGTGTCTCGATGTGCATAGGA

TGTTTCGTCCGTCGCCCAATCTGAAGCGCCTCCTTGGACCTCAATAGTGT

CGTTATCGCGGTCTGTGATAGGAGGTCTGTCATCCCTAGATGTAGTGCCG

TATGTGGCTATGAATGCACCTCTCTTGATTCCTGATGGTCTTCCGCTGGT

GCTGGCCATTTTTGCTTCGTATCGAATGTCGGCAGCGACCTCTCGATAGT

TGAGGGAATTATTGAGGATTTGAGGGCGTCGGTGTTGTCGGAAAGTAGAT

GCCCAGCTGGGGAGGACGTGATACATTGCTCGGTTCAGGTCTTCCGCCCA

GCATTGCGCGTCGAGGGCATCGGGGACTTTCTTGCTGATCGCTTCGGCCA

TGGTCGTCTCCCACTCCGTGACCCACTCGTTGAGTTTGTTGATCTTCGGG

GCTGTATGTACGGCTTTGTGGTACTTGTCGCGCGCTTCGGGTCGAAGGCG

TTCGTTGTATACCACTCCAGATCTTCGTAGCTCCTGATACCACTTGTCTA

ATCGGTCTCCCGTGACGCAGGATGTTTTCTGATACGTGGCCGAGACCGTC

TTGAGGACAAACTCGACCAGCTTCTGCTCCTGTTTCTTCGTCTCTCTATA

CGCCATTTCTTTCAGAGAGTAGATCCTGATATCATGCTCGTATTCCGCTC

TTCCTTCAGACGTCAACTCTCTGATTCGCCGGGGCGGTACGTAATCTGAG

CCGGGCGTCATTGTGCCACTATCGGGATCGTCCGGATCTGCTTGTCTGGG

GTAGTCTCGGATTTCTGGAAGTTCTGGTCGCTGAGGCCATCGGATACGAT

CGGTTGGATCAATATACTGCCACAGGTCGTAGGCGTGCGCCAGTTTCTTG

AATTCGTTGGACCAGGAGTCCCAATCCTCCGGAGACGCGTAGCTAATGGT

CCTCTCGGCGTGTTTGGCCGACATGGTGATAGAATGAACCGAAGGTCGTA

TGCATTTGGGTGCGCGGGTTGCTGAGATGCAATTGAGACTCTTAATTGTC

AGACATTACGTGTATTCGGTTCATTGATGTGAATTGGTGTATTCATGTTG

TGTTCTGTTGAAATCTGCCCCTTAGAAGTTTTACCATTTCGGCTGTAATC

TTAAAGTATCCCTGCTTGCACGTGAGGCCCCAGCACCTGTTCAACA

>Nht2-like retrotransposon type 4

TGTTGAATGTTGGAGGGTTCACCCTCAACTTCAGGATTAAGGTTCAGGAT

TAAAGTTTGACGCCCTAAACTTAGATCGTAGGACTTTCATCTAGGACTTT

CTAGGACTCAAGTCCTAGAAGGAACCAGTCAAAGATATAAGGTCTCAATG

GTCCTTCGATTGCGAAAGAATCAAGAAATATACACGATTCACCTTAATAT

CTGACAATTAAGAGTCTCATTGCCTCAACCATTTTTATTTTTATTTTTCA

ATACCTTGAAGAACCCTCTTTCTCATACATAATGTCCCTATCCAAGCCCC

AAAAGGAAGGTCTTACGGCTACCCTCTCTAAGCCTGAGGACTTCCTCAAC

TGGGAGCATGAATTCCTCCTTCAGGCCCATCGTCTTGGTCTTCAGCAGCA

CCTCAAAAGCAAGACATTCCTCGGAGACGAACCAGCTATCCCGGATATAA

GAAAACGCAGATATACGAAGACCGCTACAGCCCAACGAACAATCCGGTCT

CATACTGCCGAATCTCAAGACGGCGCCCCAGACGACATTCAGGAAACGGC

AAATGGAACATGGTCTATCTCTGACCTTACAGACCAAGGCCAGAAGATCT

TCCAGCAGGATTTAACCTTTTATCAGCTACAAGAGAAGGTTTTCGAAAAA

CAGAAAAACGCTCTTGGAACCCTTCAGGACTGGGTTATCCGTACGGTCTC

CCCAAGCTTGATTCAAACCTGCTGTCAGTCACACGAATCAATATACGAAT

GGCACTGGAACCTTCGTGCCCGGTGTGGAAGGACCGAGGAAGATGAAACG

AAGGATGCCAGAAATAGGTATCTTACATTACTAAGAACTGCCCCAAAGAC

GGTCAGCCAAGCAGCATTCAAGTGGCTAGATCAATGGGAAGAGGCAATGG

CGAAAGGGCGACAACGAGAGGTCCCTGAAACGCTCCACAACAGTGCATGG

GCCTGGGACTTCTTCAGTGTCACCACACACATCTTGCCCAGCTGGACTGC

CTCATACAAAATCTCTTCAAAGACTCTATTGCAAAATAGGGCCCTCTCAT

ACCGAGATGTAGGGAACGCCTTCCGAAATGAGCTCCAAGATGCAGAAGCC

ACGAAGGGCCGAGTGGCCCGCGGTGCGTTTTCTGCGAGCTATGCTGGGGA

GGACCCCCAGGATGATGAAGGGGACGCCCGCATCATTGAAGGCCAGTACC

CATTTTCGAAGAAGGGAGAAAGAAAACGCCAGAGGTCTCCTGAGAAGAGA

CAGGAATGCCCGGCGTGTGGGTTATCCCACGAACTTGCGAAATGCTTCTA

TGTCTTTCCAAATCAGGCATGGAAGGGATTCAAGCCCCGAGAGAAGATCC

AAAAGAAGGTGAAAGAAGCTCTCGAGGAAGACTCAGACCTGGAGGCCAAG

GTAAAAGCCCTCACTCGGAAGAAGGCCAGGACCACAAAAGACAGAACCTT

GATGCAGCCAACAAAGGAAGAAAACTCAGACCAATGACTGTACGGCCGAC

CAGTCGCCGAAATCAAGACCGCCTTTGCAGCCCAAAGGGATAACTATCCC

TTACGGAACTCCGTGATCCTCGACCCCGGAGCTACTATCTCAATCACAAA

CAGTGCCAACAGACTAGCGAGATTCCAGCAGGCTATTCCAGGCGACTTCA

TCTGGGCAGGAGACCGAAAACTACCCATCTTAGGCTACGGAACCATGACC

ATCAGGCTCACAGGAACTCGAGTCCTAATGCTTGAAGATGTAGCCTATTG

CCCTCGGCTCCTTACGACCCTAGTCTCCCTACGTCAACTACGAAAGAAAG

GCTTCTATTGGGATAATAGGCGTAACCCAACGACCCTCCGACGACTCGAC

CGAACGATTGCATGCACAATCCATGACCTATACGGCCAGTATGTCGTCGA

ATACCAGTCAGAAAGCTTCCCAAAAGCAGCCTTTGCAGCCCACAGATTCA

GCTCTCACACAAAGAGAGCCCCTTTGACGGGAGAAGCTATCCGATGGCAC

AGTCGCCTAGGGCACCCGGGACCACAAGCTCTGGAACATCTTATCTCGGC

CTCTCAAGGGGTGCGAATACGAGGACCAGCCACTGTTGAATGCGACGCCT

GTGGCATATCGAAGATCAAGAGACAAATCAGACGAGCCCCAAGACAATCC

GATCAGAAGGCCGGCGAGCGTATCGCTATTGACTTCCATGACTACCAAGA

AGGCATAGGCGGCTATACCTCACAGATGTTGTTAACCTGCCGAGCTACGG

GTTACATCTGGGATTACTACCTCACAGCCCGGACGACAGAAGCTGTTATC

ACCGCTTTCAAATCCTTCCTAAGTCATCTGGAAGTCCAACACGGCCTCAA

GGTCAAAGTCATCGAATGCGATAACGAGATCACGGAGATCAAGCCGAAAA

TCGGAGACTTCCTGAGGAATAGAGCTATCATCATCGAGCCCTCGGCCCCA

TACACACAAGCCCAGAATGGGGGTGCCGAACGCTCAGGGGGTGTAGTCAA

GGACAAGGAAAGGGCTATGCGAACAGGCGCAAAGCTTCCTCACCAGCTGT

GGCCAGAAATTGGAAGGACAGCAGTTTATCTGTACAACCGAACGCCGAAC

TACGCATCAGGCTGGAAAACGCCATATGATCAACTGCATACAGCATCAGC

CAAAGCAGCAGGGATTCCACAGCCGAAGAAACAGCCGGATCAAACACACC

TACGGGCATTTGGCTGCAAGGCCTATGCAATGACCTCAGATGCCCAGCAG

AAGGTCAATCGCAAGATGCGGCTTAAGCCCAAAGGTTGGATCGGCTTCCT

AGTGGGATATAGGTCCTCAAATATCTATAGGATCTGGATCCCCACGATCA

ACCGGATAGTCAATACTCGAGACGTCATATTTAATGAAGACGAAGGCTGC

AACGGAGACTGGGAAAGCTTCAAAGATGAACTACTCGAAGCTGACCTAGA

AGAAGTTGCCAAGTTCGTACAAGGATCCTCCCTGCCGGAACAAGACGACA

ACGAACAAGCCGAACAGGTTGAAGACTGTATTGAAGTGGTCCTTCCAACA

ACCACCCTAGCAGACGAAAGCAGCCCTATTACAGACGAAACAGAAGACTG

CATCGTGGCGACTGCCCTAGAAAACGGCCTTAAACAGTCAGAAGACTCTG

ATATCCTTCTAACACCTCCAGTCACACCACTGTCAGCGCTACTAGCGCAC

AGCTCCAGGGTCTTATCAGACGAAGGAAACAAAACAGTCTCGTGGCAAGC

AGCCTTCCTAGCCGGAACAAAGTCCCCTCTCCAGACCTCAATTGAACGGA

AGCTCCGACAAGGGGTGAAAATGCATCGAAACTCTCTACCGCCGCCCCCG

ACGAAACATCAAGACCTAAAGCATCACCCTTTTGGAATGCTCTTTCAAGA

GGCTGAAGAAAGCCACCTTCAGAGTCACAAGGAAATGAGGTCCTGGACTG

AGATTCGAAAGAAAGACGCTTGCGCCGTTGACCAACAAGTCCTTGGCTGT

ATGTGGGTATACGTTTACAAGTTCGATAAACACGGCCGATTTCAGAAATG

CAAGGCACGGCTAGTGGTCAGAGGAGACCAACAAGCTAAGGGAAGGTTGC

AAGAAACATATGCAGCTACCCTAGCCGGAAGGTCTTTCCGGACCCTGATA

GCCATTGCGGCCAGATTTGACCTAGAGATGGTCCAATACGATGTGGTCAA

CGCCTTCGTAAACGCCCCAATTGACCAGGATATTTTCATGCACATGGCCC

CGGGGTATAAGAAAACAGCAACTATCCTGAAGCTCAACAAGGCCCTGTAC

GGACTACGAGCCTCTCCCTTGCTATGGCAGAAAGAACTCTCGAAGACCCT

CCTAGAGTTAGGCTACACACAGATTCCTCACGAACCTTGTTGTTTCAAGA

AGGAAGGAGTCCTCTTCTTCTTTTACGTTGACGATGTTATCATCGCTTAC

AGGAAGGCCCGGAAAGACAAGGCCGATGAATTGACTAGACAGCTTGGATC

AAAATACAAACTAACAGGCGGAGACCCCGTGCAATGGTTCCTAGGAATGG

AAGTCATACGGGACCGACCTGCAAGGAAGATTTGGCTATCTCAAACAGCC

TACATTGACAAAATCGCAAATCTAGCTAACAAGGGAACATCTACAGTACC

AATGACTCAAACAGAGCTACGACCTCAAACAGGACTAGCAACCCCGGCAG

AACTTCAACGATATCAACGTAAAGTCGGCTCGATCCTCTATATCGCCGTT

AACACCCGGCCTGACATAGCTTTCGCCGCCTCCAGGCTGGCCCGATTTCT

TGTCAACCCTGGCCAAGAACACCAAGACGCCGCCGACCGCGTTATCCACT

ACCTCTCACACACAAGGCACCGAGCCCTACGATTCGGAGGGCCCGGAGGT

CTTACAGTTGCTAGTGACGCTTCTTTTGCCGATAACACCCTTGATCGGAA

AAGCTCCCAAGCGTACACTATCCGACTATTCGACGGGTTGATCGGCTGGA

GAGCTAGCAAGCAGGACACAGTCACCACATCCACAACAGAGGCAGAGCTC

CTAGCCCTGGCACAGGCTGCTAAGGAATCCCTCTATGTGAGTCGGCTGTT

GAAAGAGCTTACAGTTCAGATGGATGATCCGACTATCCGTATCGACTGCG

ACAATACTCAAACTATCAACCTTGTGACCGCCGAAATCGCTACTTTGAAG

ACTAAGTTGCGGCACGTGGATATTCACAACCACTGGCTGAGACAGGAGGT

CCAAGAAGGGAGAATTCAAGTCAAATACACCAAATCAACGGATATGATCG

CAGACGGATTAACAAAGGCCCTGCCGGCAGGCAGATGGAACCGCTTCCTC

TATCAACTTGGCCTGGAAGATATTCAGGAAAGACAGCAGCAGAGCCGGAA

GCAAGAAGGAATAGAAGCAAGGCTCCAGAGGATAGAAGACTGCCTAGGAA

ATGCAGGCTCAACTGGGACCTATTGAGCCTGAGGGGGTGTGTTGAATGTT

GGAGGGTTCACCCTCAACTTCAGGATTAAGGTTCAGGATTAAAGTTTGAC

GCCCTAAACTTAGATCGTAGGACTTTCATCTAGGACTTTCTAGGACTCAA

GTCCTAGAAGGAACCAGTCAAAGATATAAGGTCTCAATGGTCCTTCGATT

GCGAAAGAATCAAGAAATATACACGATTCACCTTAATATCTGACA

>Nht2-like retrotransposon type 5

TGTTGAATACTGGTGAAAATGAGACCGGCTAACCGGCTGACCGAACTACC

GGTCAGAGAGCTGTCTCAGCACTGTAACAACCTCTTAGATTGGTCATTAC

TGAACAGATAGAAATAGGGTTCCAACCCAACTATCGGACAGAGAGTGGAA

TACACACAATAGACCCCTATATCTGACAATTAAGAGTCTCACTGCCTTAA

CCAACTTTAATACCTTGAAGAACCCTCTTTCTTATAGATAATGTTCCTAT

CTAAGCCCCAAAAGGAAGGTCTTACGGCTACCCTCTCTAAATCTGAGGAC

TTCCTCAACTGGGAGCATGAATTCCTCCTTCAGGCCCATCGTCTTGGTCT

TCAACAGCATCTCAAGAGCAAGACATTCCTTGGAGACGAACCAGCTATCC

CGGATATAAGAAAACGCAGATATACGAAGACCGCTACAGCCCAACGAACA

ATCCGGTCTCATACCGCCGAATCCCAAGACGGCACCCCAGACGACATTCA

GGAAACGGTGAATGGAACATGGTCTATCTCTGACCTTACAGACCAAGGCC

AGAAGATCTTCCAGCAGGATTTAACCTTTTATCAGCTACAAGAGAAGGTT

TTCGAAAAACAGAAAAACGCTCTTGGAACCCTTCAGGACTGGGTTATCCG

TACGGTCTCCCCTAGCTTGATTCAAACCTGCTGTCAGTCACACGAATCAA

TATACGAATGGCACTGGAACCTTCGTGCCCGGTGTGGAAGGACCGAGGAA

GATGAAACGAAGGATGCCAGAAATAGGTATCTTACATTACTAAGAACTGC

CCCAAAGACGGTCAGCCAAGCTGCATTCAAGTGGCTAGATCAATGGGAAG

AGGCAATGGCGAAAGGGCGACAACGAGAGGTCCCTGAAACGCTCCACAAC

AGTGCGTGGGCCTGGGACTTCTTCAATGTAACCACACACATCCTGCCTAG

CTGGACTGCCTCATACAAAATCTCTTCAAAGACTCTATTACAAAATAGGG

CCCTCTCATACCGAGATGTAGGGAACGCCTTCCGAAATGAGCTCCAAGAT

GCAGAGGCCACGAAGGGCCGAGTGGCCCGCGGTGCGTTTTCTGCGAGCTA

TGCTGGGGAGGACCCCCAGGATGATGAAGGGGACGCCCACATCATTGAAG

GTCAGTACCCGTCTTCGAAGAAGGGAGAAAGAAAACGCCAGAGGTCTCCT

GAGAAGAGACAGGAATGCCCGGCGTGTGGACTACCCCACGAACTTACGAA

ATGCTTCTATGTCTTTCCAAATCAGGCATGGAAGGGATTCAAACCCCGAG

AGAAAATCCAACAGAAAGTGAAAGAAGCTCTCGAGGAAGACTCGGACCTG

CAGGCCCAGGTAAAGTCCCTCACAAGGAAAAAGGCCAGAACCTCAAGCAC

CAGAAACCCAAAACAAGCAACGAAGGAAGAGAACTCAGATCAATGACTAT

ACCAGAGACCTATTGCCAAAGTCAAGGCCACATTCATAACACAGAGGGAT

GATTATCCCCTACGGAACTCCGTAATCCTTGACCCTGGAGCCACTATCTC

AATCACGAATAGCCCCGATAGACTAGCCAAACTTCAACCGGCTATTCCAG

GCGACTGTATCTGGGCTGGAGACCGAAAACTGCTCATCTTAGGCTACGGA

ACTATGACTGTTAGGCTCACAAGAACTCGAGCCCTGTTGCTCGAAGATGT

GGCCTACTGCCCTCGACTCCTTACGACTCTAGTCTCCCTACGCCAACTGC

GAAGGAAAGGTCTCTATTGGGACAACCGGCACGACCCAACGATCCTTCGA

TGGCTCGATCGAACTCTTGCATGCCCGGTTCAAGATATATATGGTCAATA

CGTCATTGAATATCAACCAGTAGCTATAACCCCAGCAGCTTTGACAGCCC

ATAAATTCAACTCTTATAGCAAGAGGGCCCCTTTGACAGGAGAAGCTATC

CGCTGGCACAGCCGCCTAGGGCATCCGGGACCACAAGCCCTTGAACACTT

GGTCTCAGCCTCTCAGGGGGTGCGAATTCGAGGTCCAACCACTGCTGAAT

GCGATGCCTGTGGCCTATCGAAGGTCAAGAGGCAGGTCAGACGGGCCCCA

AGGCAGCAGCCCGGTCAGGCCGCCGGCGAACGTATTGCTGTTGACTTTCA

TGACTACGAAGAAGGCATAGACGGTTACACCTCACAGATGTTACTGACAT

GCCGAGCCACCGGATACATTTGGGATTACTATCTTACAGCCCGAACGGCA

GATGCTATTATCACGGCCTTCAAAGCCTTTCTAGGTCATATGGAACGGCA

ACACAGCCTCAAGGTCAGAGTTATCGAATGCGATAATGAGATCACGGAGG

TCAAGCCGAAGGTCGGAGACTTCCTGAGAGATAGAGCAATTATCCTCGAG

CCCTCAGCACCGCACACACAAGCCCAAAATGGCGGCGCCGAACGCTCAGG

GGGTGTGATCAAGGATAAAGAAAGGGCCATGCGGACAGGAGCCAAGCTTC

CACATCAGTTATGGCCAGAAATCGGAAGGACAGCGGTTTATCTATACAAT

CGCACGCCTAGCTACAGGTCAGGCTGGAAGACCCCATATGATCAACTACA

CACAGCATCAGCCAAACTCAGAGGGATTCCACAACCGAAGAAACAGCCGG

ATCAGACACATCTACGAGCATTCGGCTGTAAGGCCTATGCAATGACCTCA

GAAGCCCAACAGAAGGTTAATCGTAAAATGCGACTTAAGCCTAAAGGCTG

GATCGGCTTCTTAGTCGGCTATAGGTCCTCGAACATCTATAGGATCTGGA

TCCCTACAATCAACCGGATAGTTAACACTCGAGATGTCATATTTAATGAA

GATGAGATCTGCAACGGAGACTGGGAACAATTTAAGGACGACCTTCTTGA

AGCTGATCTTAGCGAAATAGCCAAGTTCGCACAAGAGTCTGCCCTTCCAG

ATCAAGACAGCCTTGAAGAACTAGAAGCTGAAGACTCCTTCGAAATAACC

CTTCCAATAGGCGCTCTGACCGACGATGAAGGAACCCAACTGGGGGAAGC

CGACGAAACAGTCGAACACATCAGCGGTCCGTATACAACGGCAAAATTTG

TCCCTTTTCCAACGCCACCAGAGTCGCCGCCATCAGCCCTTCTAGTACAT

TCTATCCAAACGCTCCATCAAGGAGTTCAACCTACAATCGAGACGAATGA

AGGTACAACAACAGTTCCTTGGCAGGCAGCCTTCATAGCCGGAGCAAAGA

CCCCTCTACAGACCTCAATCGAACGGGCGCTCCGTCAGGGGAGGAATATG

CACCGAAGCAACCTGCCGCCGCCCCCAAGGAAACACCAAGATCTTATAGA

TCATCCATTTGGAAAGCTCTTTCAAAAGGCCGAAGAAGATCACCTTCAAA

GTCATAAGGAAATGAAGTCTTGGACTGAGATACGAGCTAAAGACCCTCGA

GCCTCTGAACAGCAGGTCCTCAGCTGTATGTGGGTGTATGTCTATAAGTT

TGATAAACACGGCCGCTTCCAGAAGTGCAAGGCACGGCTAGTGGTCAGAG

GAGACCAACAAGCTAAAGGGAGGCTGCAAGAGACATATGCAGCTACCCTA

GCCGGAAGATCCTTTCGAACCCTGATAGCGATAGCAGCCAAATTCGACCT

AGAAATGGTCCAATACGATGTGGTCAACGCATTCGTACACGCCCCAATCA

ATCAGGATATCTTCATGCATATGCCTCCGGGGTATAAGAAAGCAGGGACC

ATTCTGCAGCTAAACAAAGCTCTACATGGGTTACGAGTCTCTCCCTTACT

GTGGCAGAAAGAGCTCTCGAAGACCCTCTTAGAGCTTGGCTATGAACAGG

TTCCCCACGAGCCCTGCTGCTTCAAGAAGGAAGGAATCCTCTTCTTCTTC

TATGTTGACGATGTTATCGTGGCCTACCGAAAAGCTCGGGGAGATAAAGC

CAATGAACTGATAAAACAGCTCCAATTGAGATACAAGCTAACAGGCGGTA

ACCCTATGCAATGGTTCCTAGGAATGGAAGTCATACGGGACCGACCTGCA

AGGAAGATTTGGCTATCTCAAACAGCCTATATTGACAAAATTGCAAACCT

AGCTAACAAAGGAACAGCTACAGTACCAATGACTCAAATAGAGCTACGGC

CTCGAATAGGACTAGCAACCCCAGCAGAAATTCAACGCTACCAACGCAAA

GTCGGCTCAGTCCTCTATATCGCTGTCAACACCCGACCCGATATAGCCTT

CGCCGTCTCCAGACTGGCACGATTCCTCACCAATCCAGGCAGAGCCCATC

AAGAAGCTGCTGACCGCGTTATCCACTACCTTACACACACCAAGCATCGA

GCCCTAGCCTTCGGAGGGCCCGGTGGGCTCAAAGTTGCTAGCGATGCCTC

ATTTGCAGATAACACCCTCGACCGAAAAAGCTCGCAGGCTTATACAATCA

AGCTGTTCAATGGGTTAATCGGTTGGAGAGCCAGTAAGCAAGATACAGTC

ACCACATCCACGACAGAAGCAGAACTCCTAGCCCTAGCACAGGCGGCTAA

GGAGTCCCTCTATGTGAGCCGACTGCTGAAGGAGCTTACAGTCCAACTAG

ATGACTCGATCATCCAGATTGACTGCGACAACACTCAGACCATCAACCTT

GTGACTGCCGAAATAGCTACCTTAAAGACCAAGCTAAGGCATGTGGATAT

CCACAACCACTGGCTGAGGCAGGAGGTTCAAGGAGGGAAAATTCAGGTCA

AATACACCAAGTCAACAGATATGATTGCAGACGGCTTAACAAAGGCCCTG

CCGGCAGGCAAATGGGACCGCTTCCTTTATCAACTTGGCCTGGAAGACAT

TCAGGAAAGACAGCAGAACAGGAAGCAGGAAGGAATAGAAGAAAGACTCC

AAAATACGGAAGACTGCCTATGAAATACAGGCTCAATAGGGACGTACTTG

AGCCTGAGGGGGTGTGTTGAATACTGGTGAAAATGAGACCGGCTAACCGG

CTGACCGAACTACCGGTCAGAGAGCTGTCTCAGCACTGTAACAACCTCTT

AGATTGGTCATTACTGAACAGATAGAAATAGGGTTCCAACCCAACTATCG

GACAGAGAGTGGAATACACACAATAGACCCCTATATCTGACA

>Maggy-like retrotransposon type 1

TGTCACAGCGTAAGACCAGACCAGGTTACCCTAGACCGATAGGCCAAGTG

GATCTCAGTCACGTGGCGATACGTTATCGCAGAAGATCTTGACCGACCGC

CAGATAGCTCCCGAACGTAGCTCCTCGAGCTACGTCTTGTCAATAGAGCC

TGACCTGCCGGCAGTGCCTATCCGTAGCAATAGAACCTATCCGCCTAAAG

CGTTCCCTGTTCACCTGTATTCCCGAGCCTGACCCGTGACACTACGTAGC

TCCTCCCCTTTGGACGCTGAACGCAATGCCTACCCACCCGATTCCCCCGG

CTATCCCAGGGAACCGCGCCGAATATGAGGCGCAATACGCGAAAGACCCC

GATAGATGGTATCAGTACCTAAGTGAAGCCTATGCCTGGATGGCAGCGCA

AGAAGAAGGTCAAACCGCTACTGATCGGAAGCTTATAGAGCTTCAAGTCC

AAGTCGAAGCCCAGCAGGAGGAAATTCTAAACCTCCAGAATATAATACAG

ACAATGCAAGTCGAGAAGTCCGCAGCGATGATGCAGAAGTCATGGATAGA

GGACCGACTGGATAAGAAGGAGAAGGAACTAGAGATAGCCCAGGGCAAGG

CCTATAAGGCTCAAGAGGAAGCCCGTCAAGCCGTGGCCGCCCGACTCCCT

ACTGTAGCAACCCTGACACCCGTCACATCTGACCCTGATGCTAAGGCACA

AATAGCTGAGACTATGGAAACGCCTTCACCTCCTGCTACACAATCTGCCG

CCTCCGCCCAGCTTTCAGAACGAATCCCCGACCCTGATAAGTTTAAAGCA

ACCCGCGCCGACCTACGACGCTTCTGTGACGCCGTCACTGAGAAGCTAAG

CCTGAACCGAGACCGCTATCCAACCCCTATCAGTCGTATGGGCTATGTGA

ACAGCCGGCTGAATGATGATGCCTATAAACTCATACAGCCGTATATTCGT

CATGGAATTTGCCGCCTACCTGACTATACTGATATCCTAGATATCCTGCA

GGGTGCCTATGGAGACCCTAATGCAGCCCGAACTGCACGAAGAAAGCTAG

ACACCCTTCGACAAGGCAATAGAGACTTCTTCTCCTTCCATGCAGAGTTC

CAGAGCCTAGCCTTAGAAGGCGGCATAGAAGGTGACGCCCTGGCCCCCTT

CCTAGAGAAGGCCGTCTCGAAGGAACTATCAGAAATGCTCCTCCATAACC

CGCCTGCCGATTACAGCTACCACACCCTCGTATCCCACTTCCGAGGACTA

GATATCCGCCTGCAAGAACACCGCGAGAGGACCCGACCCTATATCTCCAG

GAAACCGACTACCACAGCTTATGTTAGACCTACCTATATCCAGACCACAC

GACAGGAAAGGAAGTCTCCCTCACCTCAAAGGGGCCGTAGCCCCCCAGAG

AGAGCCCAGCCCTCAGGCGACCCTATGGACCTAAGCAACCAACGCCGCTA

CCAGCGCCCCAGCTATCGTCGGGAAAACAACCAATGCTTCCGTTGTGGCT

CATCTACTCACTATATCCGCGACTGCCCTGAACCAGATACCGGCCCAGCC

AAGTTCCGACATGCCGCTATCACCTATAGTCCGCGCCACCCCAGCCGTCA

GGAGTCTCCTAAGTCCCCGACCTCTAGCCGATCTGACTCCCGTAACTCGC

AAATCCATCAGGAAAACGGGGCAAGCCTGTCCTAAGTCGGCGACAGGCTC

TCGCCTCTCCAATACCCGCACTGAAGATCAGACTATCTACTGCCGCCGTC

CATGGGATCCCTATCGAAGACGAAGGGAACCAACGTTCTAACCTGATGAT

TCTACCAGCTAACCTAATACCGACTGGGAAAGACCCTATACCCTCCTACG

CCATGACCGACAGCGGGGCGGAAGGGAAAGGATTTATCGATGAAAGCTGG

GCAAAGTCCCAGAACTTACAGCTTTACCCCCTAAAGAGACCATTCGAGAT

AGAGGTCTTCGACGGCCGACCCGCCGAGAGTGGGCAGATTACCCACTATG

TCCGTGCCGGACTCCGGATCGCAGACCATTTCCAGAAGAGTATGCTATTC

TACGTCACCCGGCTAGCCAGCTATCCCATAGTCCTAGGGATGCCCTGGCT

AAAACAGCATGACCCCCAGGTGGGCTTCGCAACCCACACCTTCACCTTTA

ATAGCCCATACTGCCAGAAGTTCTGTAATACGCCAGCTAAACCTACAAAA

ATCAAAGCCCTACAGACCATCCCAAAGAAATTCCTCCGCCAACTAGAACA

GAGCATCCCTAAGAACCTAAGGAAGAAGGATATCCTCCCTATATCCCTAA

AGGCTGTCCGATTATACAGCCAGAGACCAAGCTGCCGCTTCTACACCATC

ACACTCGAACAGATTGACCGAGGACTACAGGATAAGACCGAAGATTTCCA

GTTGCCCGAGGAATTACAAGAATTCCAAGACGTCTTCTCGCCTAAGGAAG

CCGAGAAGCTACCCCCGCACCGATCCGGAGACCACCACATCGAACTGGTA

CCTGGAGGGAAGCTACCCTTTGGGCCACTATATGGAATGTCTCGTGAGGA

GCTGAGAGCCCTACGAGAGTGGCTTAGGGAGAACCTACGCAAGGGATTTA

TCCGACCTAGCTCTTCGCCTGTCGCGTCGCCAGTACTTTTTGTAAAGAAA

CCCGGCGGCGGACTTCGATTCTGCGTGGACTACAGAGCCCTAAACAACAT

CACAGTCAAGGACCGCTACCCGCTGCCCCTGATCAAAGAGTCCCTAAACA

ACCTATCCGGCATGAAGTACTTCTCCCGTATAGACATCGTATCTGCCTTT

AATAACCTCCGAATAAGGAAGGGCGAAGAATATCTCACAGCCTTCCGAAC

ACGCTTTGGATTATACGAATCCCTAGTCATGCCCTTCGGACTAACAGGAG

CCCCTGCAACCTTCCAACGTTATATCAACGACGCCCTAAGAGAACACCTA

GATATATTCTGTACAGCCTACCTAGATGATATCCTGATATACAGCCGAAC

CCGAGAGGAACATATAGAGCACCTTAAGCTGGTGCTCCAGAAGCTCCGAG

CCGCAGGGCTCTATGCCAATCCTGCTAAATGCGAATTCCTAGTTAAGGAG

ACGAAGTTCCTAGGCCTAATCGTGGGCCAGGAAGGGATTAGAATGGACCC

TTTGAAAGTCGAGACGGTCAAGAACTGGAAGACACCGACCTGCCTGACAG

ATGTGCAAGCCTTTATCGGCTTCGGGAACTTCTATAGGAGGTTCATCCGT

GACTTCTCAAAGCTCACGGCACCCTTGAACAGACTGACGAAGAAGGACGT

GCCCTTCGTATGGGATGATGCCTGCGAGAAAGCTTTCCTAAAGCTGAAGG

AAGCCTTTACAACAGCCCCGATCCTACGCCCGTTTGACTGGACCAGAGAT

GTAATCCTTGAGACCGATGCCTCTGACTACGTCTCGGCGGGAGTGCTATC

GCAATATGACGACGAAGGAAGGCTACACCCGGTAGCCTTCTTCTCAAAGA

AACACACAGCCACCGAGTGTAACTACGAGATATATGATAAGGAACTGATG

GCAATTATCCGCTGTTTTGAGGAATGGAGACCCGAACTAGAGGGCGCACC

CTCGCCGATCAAGGTGATCACAGACCACAAGAACCTAGAGTACTTTACGA

CAACGAAGCTACTGAACCGAAGACAAGCCCGTTGGTCCGAGTTCCTATCT

CGTTTTAATTTTCAAATTAGCTATCGACCCGGAAAGCAAGGAGTTAAGCC

CGATGCTCTGACCAGGAGGTCAGAAGACCTGCCTCAGGAGGGGGATGAGC

GCCTGGCGCATCAGAGCCAGGTAATCCTAAAGAAAGAGAATTGGCAGCTT

CCACCCTTGAAGGTTCAGAGGGCCCGTATACGCCAGCCGCACCTACAAAT

ACCTGCAGCCCCAGCAGAACCAAGTCCTTCCATAGACTTGCCGGGGGATA

TCGACCGCCTATTAGAAGAGGGTTACCAGACCGACGACGACCTACAGTCA

ATACTGCAGGCCGTAAGAGAAGGCGCCCAGCGCCACCCGAAGATCACCCT

GGCCGAGTGCACTATCGTACAAGGCCGACTACTCTATCGAGACCGCGTTT

ATATTCCAGACCACAACCCCCTGAAGGCTGCCCTGCTGAAAGCAAGTCAC

GAACACCCCATAGCAGGCCACCCAGGCCGCGCCCGCACTTATGACCTACT

CTCACGCGACTACTACTGGCCTGGAATGCTATCTTATGTAGAACGATGGG

TTAAGAATTGTCACACCTGCCGAAGATCGAATCCCGCCCGAGATGCAAGA

CAAGGAGTCCTAAAGCCCCTGCCCGTGCCAGAGCGAGCTTGGCAGCACCT

ATCAATGGACTTTATAACCCACCTGCCACCTAGCGAAGGGAACGATGCTA

TCCTGATCATAGCCTGCCGACTTACGAAGATGAGACATATTATAGCCTGC

AAGGGAACCTGTGATGCCGAAGATACCGCCCGATATTACCTAAAGGAAGT

GTGGAAGCTACATGGCCTTCCACAGACTATAGTATCTGATCGAGGACCTC

AGTTTGTGGCTGAATTCTGGAAGAAGCTTAACCAACAGCTATCCATCAAC

GCCCTTCTATCAACCGCATATCACCCCGAAACCGATGGACAGACCGAGCG

GCTAAATGCCATACTAGAGCAGTACCTGAGAACCTATGTGTCATACCTGC

AAGACGACTGGAGCCGATGGCTCCCCCTGGCCGAGTTCGCAGCGAACTCA

TTAAAGTCTGAGACCACTAGGATATCCCCGTTCTTCGCGAACTATGGATT

TAACCCCCGGATGGGCTTTGAGCCTACTATCACAGTCAGAGGAACCCCTG

CCACGAGAGATGCGGAACAGTTCGCACAGACGATGAACGAGATACTAGAG

TACTTACGCTCCGAGAGCATAGCCGCACAAGCCCGTTACGAAGACCAGGC

GAACCGTCATAGAAGACCCGCCAGATGCTATCGAGAAGGCGACTATGTCT

GGCTCGATGCCCGGAATATCAAGACCTTGAGGCCACAGAAGAAGCTAGAC

TGGAAGAACATCGGTCCCCTTAAGATCACAAAGGTCATCTCCCCATATAC

CTACCGGTTAGATCTGCCTGCGAGCATGAAGATACACCCGGTGTTTCACA

CGAACCTGCTCAGACCCGCCGCCACCAACCCCTTGCCAGGCCAGAACCCA

GACCCGCCGCCGCCTATCGAGGTCGAAGGAGTGGAAGAGTGGGAAGTGGA

AGATATCCTCGATTCCCGTTGGGATCGCCGCGGCCGTGGAGGCCGCCCCA

GACTGAAGTACATAGTGAAATGGGCAGGATACACCGACCCAACTGAAGTC

CCTGCCGACTACGTGGATAACGCTGCTGAGGTCGTAGCCAACTTCCACAG

GAGGTATCCCGATAAGCCGGGACCGCAGAAAAGGGCCTGATGGATGAGAC

CACGGAGTGCCCATCTCGACAGAGCTCGATGCTAGAGAGGGGGATACTGT

CACAGCGTAAGACCAGACCAGGTTACCCTAGACCGATAGGCCAAGTGGAT

CTCAGTCACGTGGCGATACGTTATCGCAGAAGATCTTGACCGACCGCCAG

ATAGCTCCCGAACGTAGCTCCTCGAGCTACGTCTTGTCAATAGAGCCTGA

CCTGCCGGCAGTGCCTATCCGTAGCAATAGAACCTATCCGCCTAAAGCGT

TCCCTGTTCACCTGTATTCCCGAGCCTGACCCGTGACA

>Maggy-like retrotransposon type 2

TGTCACAGCTCGACCAGACTGGCCTACCCTAGACCACTAACAAGGTAGAT

CCGGATCACGTGGCGATACGTTATCGCAGAGACTTTGTTTACCGACCAGA

TAGTTCCTGAACGTAGCTCCTCGAGCTACGTCTTGTTAATAGAGCCTGAC

CTGCCTGCAGTGCCTATCCGTAGCAATAGACCGTATTCTGCCTGAAGCGT

TCCCCGTTCACCTGCCCTACCGAGCCCAGCCCGTGACACTACGTAGCTCC

CACCAATTGGACGCCGAACGCAATGCCTGCCCACCCGACTCCCCCCGCTA

TCCCAGGAAGCCGCGCCGAATATGAGGCATGCTATGCAGAGGACCCCGAT

AAGTGGTACCAATACCTAAGCGACGCCTACGCCTGGATGAAAGAACAGGA

ATCCAACCAGGTCGCAGCCGATAGGAAGCTCGTAGAGCTCCAAGTCCAGG

TCGAGACCCAGCAAGAGGAAATCCTGAACCTCCAGAACACCCTACAGGCG

GTACAGATCGAGAAGTCCGCCGCCATGATGCAGAGGTCATGGGTAGAGGA

CCGGCTTGATAAGAAAGAAAAGGAACTAGAGGCGGCCCGGGATGAAGCCC

GCCAAGCTATAGCCTCTAGACTACCTACTGTATCAGCCCTAACGCCCTCC

GCTACGCCTGATCCCCTTGCTAAGACGCAAGCAGCTGAGATTGTGGGAGC

GGCTCCGCCTCCTACTACCCGATCTACCGCCTCCGCCTACCTTTCAGAAC

GACTACCCGACCCTGAGAAGTTTGAAGCTACCCGCGCAGACTTACGCCGC

TTCCGTGATGCCATTACAGAGAAACTAACCGTTAATGAAGACCGATACCC

GACTGCAGTGAGCCGTATGGCTTATGTGAATAGCCGACTGAACAAGGAAT

CCTACAAGCTCATACAACCTTACATCTTTAAGGGAGCTTGCCGCCTTCCA

GACTATCAGGATATCTTGGATATCCTACAGGGCGCCTATGGGGACCCGAA

CGAAGCCAGAACAGCACGACGGAAGCTAGATGTTATCAAACAGAGAGATA

GAGACTTCTCTACCTTCTATGCCGAGTTTCAGAGCCTGAGCCTTGATAGC

GGCCTTGAGGGAGATGCCCTGGCCCCTTTCCTCGAGAAGGCCGTATCGAA

AGAGCTATCCGAGATGCTCCTGAATAACCCCCCTGCCGATTACAGCTATA

ATACATTAGTATCGCACTTCAAGGAACTGGATATACGCCTGCAAGAATAC

CGTGAGAAGAGCCGACCCTATATCCCTAAGAGGTCGAACCCTATACCTCG

TGTAGGTCCTACACGCACACGGGCCGAGAGACAAGAGAGGAAGTCCCCCT

CGCCCCGTAGGGGTCGAAGCCCCCCTGAGAATCCCCAGCTTGCCGGAGAC

CCCATGGACTTAAGCAATCAACGACCGCTATATCCGCCCGAACTACCGCA

GAGAGAATAACCAGTGCTTCCGTTGCGGGTCGTCATCTCACTACCTCCGC

GACTGCCCTGAACCCGACACCCGGCCTGCTAAATTCCGACACGCAGCTAT

TCCCCAGAGCCCTTACCGCTATAGCCGTCACTCCTCTCCAAGATCCCCGG

CTCTAGACCGATCCGACTCCCGCAACTCACGAAGGCACCAGGAAAACGGG

GCGAGCCTGTCCTAAGTCGGCGGCAGGCTCCCGCCTCACTACCACCCGCA

CTGAAGATCAGACTATCTGCCGCCTCGGTCCATGGGATTCCAGTTGAAGA

GGAAGGGAACCAACGTTCTAACTTAATGATTTTACCTGCTAACCTAATAT

CAACTGGAAAGGAACCAATACCTTCTTACGCTATGACAGATAGCGGAGCA

GAAGGGAAAGGATTCATTGATGAAAGCTGGGCTAAGTCCCAGAACCTGCA

ATTCCGCCCCCTGAAGAGGCCTTTCGAAATAGAAGTATTCGATGGCCGCC

CTGCCGAGAGTGGACAGATCACCCACTACGTCCGTGCCGGACTCCGTATC

GCCGACCACTACCAGAAGAATATGTTATTCTACGTCACCCGGCTAGCCAG

CTATCCTATAGTCTTAGGAATGCCCTGGTTAAAGCAACACGATCCACAGG

TAGGCTTCGCAGCCCACACCTTTACCTTTAATAGTCTATACTGTCAGAAG

TTCTGTAATACACCAGCCAAGCCCACAAAAATCAAAGCCCTACAGACCGT

CCCAAAGAAATTCCTCCGCCAGCTAGAACAGAACATTCCCCAAAGCCTGA

GGAGGAAGGATATCCTTCCTATATCCCTACAAGCTGTCAGACTATATAGC

CGGAGAACCCGCTGTCGCTTCTTCGCTGTCACCCTGGAACAGATAGACAG

AGCATTACAGAATAAGAGTGAGGATCTGAGACTGCCCGAGGAACTACGAG

AGTTCCAGGACGTATTTTCGCCTAAAGAAGCAGAGAAGCTACCTCCGCAC

CGAGCCGGAGATCACCACATTGAACTGATACCCGGAGGGAAGCTGCCCTT

CGGCCCGCTATATGGAATGTCCCGAGAAGAGTTGACAGCTCTACGAGAGT

GGCTCGATGAGAACCTGCGCAAAGGATTCATCCGACCTAGCTCTTCACCC

GTCGCCTCCCCAGTGCTGTTTGTCAAGAAGCCAGGCGGCGGGCTACGATT

CTGCGTGGACTACAGGGCCCTGAATAATATCACCGTAAAGGACCGCTACC

CGTTACCCCTGATCAAGGAATCCCTGAACAACCTGTCCGGCATGAAGTAC

TTCTCCCGCATAGATATTGTATCCGCTTTTAACAACCTCCGGATCGAGAA

GGGCCAAGAATACCTCACGGCCTTCCGGACCCGTTTCGGATTATATGAGT

CCCTGGTCATGCCCTTCGGCCTGACAGGAGCCCCTGCAACGTTTCAACGT

TATATTAACGACGCTCTGCGAGAGCACCTGGATATATTCTGTACAGCTTA

CTTAGACGACATCCTGATTTACAGCCGGACCCGGGAGGAGCACATAGAGC

ACCTCAAGTTGGTGCTCCAGAGGCTGAGATCCGCTGGGCTCTTCGCAAAC

CCATCGAAGTGCGAATTCCTAGTGAAAGGGACGAAATTCCTGGGCCTGAT

CGTGGGCCAGGAAGGCATCAGAATGGACCCCGCGAAGGTTGAGACTGTTA

AGAACTGGAAAACACCGACCTGCCTGACAGATGTACAAGCCTTCATTGGC

TTCGGGAACTTCTATAGGAGGTTCATCCGTGACTTCTCAAAGCTCACAGC

ACCCCTGAATAGACTGACAAAAAAGGATGTACCCTTTATATGGGACGATA

CTTGCGAGAAGGCCTTTGTAAGGTTAAAGGAAGCCTTTATAACGGCACCA

ATTCTACGCCCGTTTGACTGGACCAAGGACGTGATCCTTGAGACCGATGC

CTCTGATTATGTCTCAGCAGGCGTACTCTCACAGTATGATAACGAAGGAA

GGTTACACCCGGTGGCGTTCTTCTCAAAGAAGCACACTGCTACAGAGTGT

AACTACGAGATCTACGACAAGGAACTGATGGCTATTATCCGCTGTTTTGA

AGAATGGAGACCTGAACTGGAGGGAGCACCCTCACCTATCAGAGTTATTA

CAGACCACAAGAACCTCGAGTACTTCACGACCACGAAGTTGCTGAATAGG

AGGCAGGCTAGGTGGTCTGAATTCTTGTCCCGCTTTAATTTCCAGATCAC

CTATAGACCAGGGAAGCAAGGAGTTAAGCCCGATGCCCTGACCAGGAGGT

CAGAGGACCTCCCTAAAGAGGGGGATGAGCGCCTGGCGCACCAGAGCCAA

GTAGTCCTGAAGAAGGAGAATTGGCAACTTCCACCGTTAAAGGTCCAGAG

AGCCCGCATCCGACAACCGCACCGACAAGGTTCAATACCAATAGAGGAAC

CCATCCTTTCCTTAGAGCTACCAGAAGACATTAGCCGCCTGTTAGACCAA

GCCTATAAGACCGATGAAGATCTACAGTCGATACTGCAAGCCCTAGAAGA

AGGAGCCCCACGCCACCCGAAGATCACACTTGCCGAATGCAAAGTAATAC

AAGACCGACTGCTATACCGAGACCGCGTTTATATTCCGAATTACGACCCC

CTGAAGACCGCACTCCTGAAGGCTAGCCACGAACACCCTATAGCTGGTCA

CCCAGGCCGCGCCCGTACTTATGATCTACTCTCCCGCGATTACTACTGGC

CTGGAATGCTGTCATATGTTGAACGATGGGTTAAGAACTGTCATACCTGC

CGAAGAGCCAATCCTGCCCGAGAGGCAAGGCAAGGAGTCCTAAAGCCCCT

ACCGGTACCTGAACGAGCCTGGCAACACATATCAATGGATTTTATCACCT

ACCTGCCGCCCAGCGAAGGACACGACGCCATATTGATTATAGCCTGCCGC

CTCACGAAGATGAGACATATTATCGCCTGTAAAGGGACCTGTGATGCAGA

AGACACCGCCCGTTATTACCTGAAAGAGGTGTGGAAGCTACATGGCCTCC

CACATACAATTATATCAGACCGGGGACCCCAATTTGTGGCTGAGTTCTGG

AAGAAGCTTACCCAGCAGCTATCAATCAACTCGCTGCTATCAACCGCCTA

TCACCCCGAGACCGATGGACAGACAGAACGCTTAAATGCGATATTGGAAC

AGTACCTGAGAGCCTATGTATCTTACTTACAAGATGACTGGAGCCGATGG

CTCCCTCTGGCCGAGTTCGCTGCGAACTCCCTGAAGTCTGAGACGACCGG

TATGTCCCCGTTCTTCGCGAATTACGGATTCAACCCGCGGATGGGCTTCG

AGCCTACAATTACAGTCAAAGGAACACCTGCCACAAGAGACGCTGAGAAG

TTCGCGCAAAAAATGAATGAGATACTGGAATACCTACGCTCCGAGAGTAT

AGCCGCGCAAGCCCGCTACGAAACCCAAGCGAACCGTCACCGACGACCTG

CCAGACAATATCGTGAAGGAGATCACGTTTGGTTAGACGCCCGAAACATC

AAGACATTGAGACCGCAGAAGAAGCTAGACTGGAAGAACATCGGCCCGCT

TAAGATCACGAAGGTTATCTCCCCGTACGCCTACCGACTGGAACTGCCTG

CCAGTATGAAGATACACCCGGTATTTCACACAAACCTGCTTAAGCCCGCC

GCTACCGACCCGCTGCCGGGCCAGAATCCAGACCCGCCGCCGCCTGTCGA

AGCTGAAGGAGTAGAGGAGTGGGAAGTCGAAGATATCGTAGATTCCCGCT

GGGACAGACGCGGCCGCGGAGGCCGTCCTAGACTGAAGTATACCGTGAAA

TGGGCTGGATACGCAGACCCAACAGAAGTCCCCGCAGACTATGTGGAGAA

CGCCGCCGAGGTTGTGGCTAACTTCCACAGGAGATATCCCGATAAACCTG

GACCACAGTGACCTATCTCGACAGAGCTCGATGCTAAGAGAGGGGGGTAC

TGTCACAGCTCGACCAGACTGGCCTACCCTAGACCACTAACAAGGTAGAT

CCGGATCACGTGGCGATACGTTATCGCAGAGACTTTGTTTACCGACCAGA

TAGTTCCTGAACGTAGCTCCTCGAGCTACGTCTTGTTAATAGAGCCTGAC

CTGCCTGCAGTGCCTATCCGTAGCAATAGACCGTATTCTGCCTGAAGCGT

TCCCCGTTCACCTGCCCTACCGAGCCCAGCCCGTGACA

>Pcretro3-like retrotransposon

TGTTGAGACAGTGTGGCTAAGGTGGTCCGACCTACTGGTGGCTGCGATGT

GGATACTATGTAAGGAGCTGTGCTCCTCTCAGACAGATGTAACTTAGAAC

TAATAACAACACAATCAACCCGTTTTTGCTTCAACAGGTTATGAGCCCGG

AGGGCACGACCTTTTCTCCAACCAAAAAGCTGAGTGTTTTCTGTCTCCTT

TCTACTTTCCGAGATAACACAAGAAGTCGAAAGACTTCCTTCCACTGCGC

ATACGCATTCAGCTTAATTGGAGCTATTTCCCTCTTGGATCTCTGATAGA

GCTTGCAGATCCGCTAACAGGATATGTCTAGAAACGTTTACAGAAAGATC

CTTTGACCCTCCGTTTATTGCAGCGAATTTAAATCGTCGAATCTTGAATA

ACCAAAATTTTCCCAGATTGCAATCTGGTAGACGGATTCGCCAGACCAGA

CCGGCCTTTCAGTCTGAGATTGCTGAAGTTCTCCGGTCGGGCGACCTACG

CCCACATAGCCCAAGAATGCCCGAAACGTGGTTTGCAACATGATCGACAT

CAGATCATTGCCCTTCCTCCAGACGGAGGGAATGATATGGATATCTGAGA

CCGCTAGGCGTTTCACCCTTCGGACAATCTCTACCCGACTCCGATAACCC

CTATTTGCACGGCATTATCATGTTGCCGAACGCAGCCAAGATTCATTCCC

AAGACTCGATCCAAGACTGGTCGATTGCCGTTCGCCCTCATGTCTCTTAA

TCATTAGCTTCTACGAGAATCTATCATGTCCCTTTCCAAAGAATCGCTCA

ATCTCATAGGCAGTTCCGGCACCATTCCAAAGCTGGAGCAAACCAGCCAG

TGGCTGACCTGGAAGAGAGAAATGCTCGACCTTCTGGACATGTGTGGATA

TGGAGACCTTCTGACCAGGAATGCTCAACCACCAACAGAAGGTAACGACC

TTGCAACCCAGATTGAGGCCTGGAGGAGCCGCCAAGATCGAGCATGCGGT

GCCATTCGAAGTCGTCTCGGTTATAACGCCCGAGTGTTTACCACGGGGAT

CTCGACGGCTCAGGGCATGATCAACCACCTTGAGACCCGATATCGGCCCG

TCGGAAGTGCCATCTTCCAAGAGCTCGACCGCAGATATCAAGAACTCACG

CTTGAATCTTGCGATTCGGTCATGGAATACGCAAATAAGCTACGTCAAGT

CAGGGCTGAACTCTTGGAGATGGATGACACCTGCCAGATCGGGGAACCGC

ATTTTGTCAATAAGTTCCTTTGTGGACTTGGTCCGGACTATGAAGTGTTT

CTCACTGCCTTCAATCAGAATCACAACATCCTTCCTATTCGAAATCCGAG

CAATCGCAACGTTGTTCTCAAAGAGGCCGTTACGTTTGAAACCGCCATCT

TTGCCGCATCTCAGGAGGAGGACAGACAAAGAAGCGCCACGGCCAAGATG

GCACATAGAGCCATGATGGCCCAGGATACATCATGTTGTGGATACTGTGG

AAGAAAAGGGCATGACAGGTCCACTTGTTGGAGGCTCCACCCAGAACTTA

GAAGAGATCGTATCAAAACTGGTTACGAGAGACGACAACGTAAAATGCAG

GTTCGGGAGAAGGAAAGGGAGAAGACGAATGATGAACTGGAATCTGCCAC

GTATACAACTCCTGCACACCTCCAACCTGACCAAGCTCGACTGGCACTCT

GGCAAAATGACTTCCAAGGCCACCTTGGATTGATTGGCTCATCACTCGGA

CCTTCCGGTCTTCAAGCATCTCTTCAAACTCGGGCCCTCGAACGTATCCA

CGTCTTGGATAGTGGCGCCTCCTCGCATATCTTTTGTCGACAGGAGAACT

TTACATCACTTCACAGGTACCACGGTCTCCCCATCAATGGTATTGCGGAC

ATAAAGGTCATGCCAGGAGGGCGCGGAACGTACTGCCTCCGCGTTCAAGG

GCCTAAGGGATCACAGAATGTTACTCTCGATAACGCTCTTTATGTTCCAG

AAGGACATTCGAACCTCTTGTCTGTGTCAGCACTAGAGAAGAAGGGAGCA

GAAGTTGTCTTTCGGAATGGGAAGGCAGTGGTTACAAACAAGGGAAAAGT

CGTCCTCACTGCGACAAGAATATCAGGCGTATATGTAGTCGATGAAGTTG

AGGACCGTCCTTTTCAAACCGCTTTGGCATCTTTTTCGGTGGGAGATCCT

CGACTGCACCTTTGGCACGAGCGACTCGCACATTTGGGAGAGAGGAATAT

CAAGAGACTGATGAATATGTCTACTGGAATTCGACCGGATGATTCTACAT

CAAACCCATGTGGAGCCTGCGTACAGGGCAAACTCAGGGAATGTCCCCAT

GTCCGACCGATCAAGAAAGGCACTTACCCTCTGGAGTGTATTCATGCAGA

TATTGCCGGACCATTCCCAGAAGTGGGAGTAGACGGCAGCCGGTGCTGGG

TTTGCTTTATTGATGACTTTACTCAAATGTCCTGGGTGTACCCAGTCAAA

GAACGGTCAGAGTTTGAAGAGTGCTTCCATCATCTGTTGCAACAGTTCGA

GAGACCGGAAAGACGATGTCACTTCCTGCACCTCGACCGAGGGGGAGAGA

ATCGAAGCCACAGTTTGACCCGACTATGCCGGGACAGAGGAATCAAACTC

TCTTACACAGGAACTGAGCAACACGAACAAAATGGATTGGCTGAAGTTCT

CAATCGAATCATCCTGGAAAAACTCACACCGACACTGCTCAAATCCGGAC

TCGCTTTGAAATGGTGGCCGTACTTCCTCAAGGCAGTGAACCGCATCCGA

AATCTCTGTCCTACGGCGAGCATTTCTACTACTCCTTACCAAGCCTGGTA

TGGAGATGTTCCTGACCTCAGCCATCTGCGAGTTCTTGGCTCACGAGGAT

GGGCTCTGCTTCCATCCTCTAAGCGAAAGAAGATGCAGTCGAAGAGTATT

CCCTGTCAGCTTCTCGGCTATCAGGGCAGTACAAACTATATACTCCTTGA

CGAACGGGGACGAGTCTTTGTGTCTAACAATGTTGTCTTCTCTGAAAACA

TCAAAAATGTCGAACCAACACTCGACAATCTCACGCATCAAATGAGTGAC

CAGAGACCATCACAACGACGACGAGTAGTCGGAGAAGAGGATAGCGATGT

GGGAAATGCCATCGTTCTAGAGCCGTCGCCCCTTCCGATCGCGTTGAGAG

AGGCGAACAGAAGCGGGGAAACAGGCCGTGGAGAGACGACGCCGGATGAG

CCCCCAGTGGGAGATACAGGATGGGATCCAGACCAAAAGTCAACCAGATC

GAGTATTCATGTTGCCACAGACGAGCGCGAAGTGTCATCGGAAGAAACAG

AGGAGCATGCAGAGTCTCGGCTGCCCGAAAGATCGACCAGGGGACAGCTG

CCATCGAGATACATACTCGCCAGCACAAACTTCGCCTTCCTAGCAACGGT

TGCAGAACAACTGTATCTAGTGGCTCGCGATATCGATCAGTCCGAGCCAA

GAACCTTCAAGGAAGCCACGCAGGGTGTACATGGAAAACTCTGGTGGGGA

GGATTGAAGGATGAACACGGATCTCTGGTCGAGAATGGTGTCTGGACCTT

AGTCACGCTTCCCCCAGGACGAAAGGCTCTTCGAGGCAAATGGGTGTTTA

AGCTCAAAAGAGGAGAAAATGGGCAAGTTCTACGGTACAAAGCGCGATTT

GTCGTGAGAGGATTCGAGCAAAGGGAAGGAATTGACTTCAACGAAACCTT

CGCCTCTGTTGTCAAGCCGATGAGCTACAAGATGATATTTGCCATCGCGG

CAGCACTCGATTTGGAACTGGAACAAATGGACGTCAAGACAGCATTCCTC

TATGGCCTCGTAAATGAGGAGATATACGTCCAGCAACCCGAAGGATTCGA

CGATGGAAGTGGGAGAGTCTGCAAGCTCTTGAGAGCCTTGTATGGACTGA

AGCAAGCACCAAGAGTGTGGTATGAGACTCTCTCGACATTCCTCGCGACC

CTCGGGTTCAAGCCTTTACTTTCAGATATGGGAGTCTTTGTGAAAGGTCA

CACGTTCATTGCGGTCTATGTGGACGATCTACTTATTGCCGGGCCTCCAA

GGCGAGATCGTGGCGGTCAAGCAAGATCTGTGCAATAAGTTCAAGATGAC

GGACCTGGGCCCATGCAAATACTACCTGGGCATGTCCGTAAGAAGAGACA

GAGCCACCCGATCGATCTTCTTGTCGCAGATGACCTACCTCGAGAAAGTA

CTTAGAGACTTTGGAATGGACCAGTGCGCTCCCAATGCCACACCAGTTTC

GACTAGCAAGTTCGGAGAACCAGGCCCTGACTACAAGGCAGCGGACAAAC

TCAAAGAGTGGTATGCCAAGGCGATTGGGTCCCTGATGTATCTCATGCTG

GGGACGAGACCCGACATTGCTTTTGCTGTCTCCTTATGCAGTCGACACCT

AGCGAACCCAACCAATGAGCATCAGACAGCTGTCAAGCGTATCTTTCGAT

ACCTTAAGGGATCGCAAAACCTGGAACTAGTGTACCAAGGAGGACTGCAA

CCTCTCCTCGGCTACACCGACTCGGATTGGGCGGGAGACCTGGAGACTCG

AAGATCAACGTCTGGATATGTGTTCAACCTTGGTACGGGCGCTATCAGCT

GGAGCTCGAAGAGACAAAGAACAGTGGCCCTGTCTTCATGCGAAGCAGAA

TACATGGGGCAAACCGCAGCCGCAAAAGAAGCAGTCTGGCTGCGAGCTCT

GCTCCAAGAACTGTTGAAAGAATACAAGGATGTTCCAGAACTAAAGACAA

CTGTAATTTTCGGGGACAACCAAGGAGCGATCGCAATGTCGAAGAATCCG

CAGTTCCATACAAGAACCAAGCACATTGATATTCAGCATCATTACTGCAG

AGAGAAAGTCAATGATGGAACCATTGAGTTCCAGTACATTCCGACAGGCA

AACAAGTGGCCGACGGTCTAACAAAGGCGTTGCCAAAGGATCGTTTCCTA

TTGTTTAGACAAGCACTAGGTCTCAAAGAACGACGACCATGAGAAGGTGC

AGAGATGAACCAAAACAGGAGACCTGCCATGAACATACAGGAGACCCACG

GATCTAAGATTGCACGGAAACGTGAGGAAGTCGCAAAGATGAATTAGAAT

GGGAGAGCTTCTAAACAAGTAGAAGGAAATCCATGGTCTCGAGGTTGTGC

CGAGCGTGAGGAACGTGCAAAGATGGACTGAAGTGGGAGACCCATCATGG

ACAAGAGACCTACAATTTCAAGAAAGACCTCAAGACCTGCAGAGACGGGT

GTAGGCAGGGGATCTTCTGCGGATAGAAGACCTGATAGAAGACCTGCAAC

CCCAAGATCGCAAGACCTGCAGAGATGGGCATAGGCAGGAGATTTTCTGT

GGACAGAAGAAGACCTGCAGTCCCAAGAAAGCTTGCGAAACCTGCAGAGA

TGGGTTTCAGTGGGAGATCTTCTGTGCATAGAAGAAGACCTACGATGCCA

AAGAAAGATTGCAAGACCTGCAGAGACGGGCAGAGGCAGGAGATCTTCTG

TGGATAGAAGAAGACCTGCAGTCCCAAGAAAGCTTGCAAGACCGTGCAAA

GATGGGTGTCAGTAGGAGATCTTCTGTGGACAAGAAGAAGACCCGCAAGT

TCAGGATCGCACTGGACTGAAAGGAAAGGTGCAAAGATGAACTTTGAGGG

AGATCTTCTGCGGACAGAAGAAGACTCACTGCTCCAAGAGTGCACGAGAC

CATTAAGAGATCCTGAGCGCCCATGTCGAGATAGCTCTTTCCCTGCAGTT

TTCACCCACGAAGGTGTTTTTGGGGTTTTAACGAGGCTCGACAGGCGTGC

ACATCAAGCCCAACAGCAGCTCGAGTGGGAGTGTTGAGACAGTGTGGCTA

AGGTGGTCCGACCTACTGGTGGCTGCGATGTGGATACTATGTAAGGAGCT

GTGCTCCTCTCAGACAGATGTAACTTAGAACTAATAACAACACAATCAAC

CCGTTTTTGCTTCAACA

>Yaret1

catgggaaggacaggagcttatcaggccccccaagaaggctcgattgttt

gagaaacgcatcaagtataagcaggttatcgtgggtatcctgaccaaaga

agtgtgtgtggaggttggttagcctaccatcATGGCGGACCCAATGGGAG

CCGAGATCGTAGTGGAGCAGGACGCCCCTTTTTCGACGCCAAAGCGCCCG

AGTCGAATGGTGAGGCCAACGAGCAAAGTCCGAGACACCGCGCGACAATT

GGAAGACACTGCCAAAGAAACGCGAAAAACTACCCGGCAGACGACGCGAA

ATGTACCGGCCGAAGAACAGATCGACCGACCGACCGAAACCCGAAAGAGT

AGCGGCAGCGGCAGCGGAAGTAGCGATGGAAGGGCAATGCTGCAGAAGGC

GCTGGACCTCCTGGCGGAGTCGCGCAGGGAGACCAAGAGGCTGCAGGAAG

CGCTGAAGGAACAGATAGAGATGTCCAGGGAACTTAGGGAAGCTGTTGCA

AAGCAGGAAGAGACAGTGCACGAGATGAGCAAGCAGATGGTGGAGATCAA

GGAACAGATGACCGAGGAACTGCAGCGAGCCCGCGAGCAGCTCGAAACTA

TCACCACGAATGCATTGGACGGACCTCAACGATCATATGCTGACGTCACA

CGACTGACACCGTTCTTACCCCACAACGATTCGAGGACCTTAGCCGCTCC

GCCGAACCCCACAGACATGCTTTACTGCACGATTGACGTTTCAAGACTAG

GAAAAGATGAGACTAGGCTCTCAGCCGGGACGATCCGAGCGACAGTGGAG

AACGAAGTCCGCTCCGAACTGGATAATCCCACGTGGCGATGCCGAGCGGT

GACTAGGGACCCAAAGAATCCCCACCGCATCAGGATCACCTGCAGGGACG

AGAGCGAGCATGAGATTGTGAAACGCGCAGCGGAAACAAAACTGGCCCCA

GGAGCCCGGATCCTACGAGACGACCTGTACCCTATCAGAGTGGATAATAC

CAGCCGTATTGCGGTGCTAAACGAGAGGAACGAAGTTCGAACTGAGATCA

CCGAGATGCTCGGCCGAGAAAATGACACCGAGGTTGCAAAGATAGCATGG

CTCAGCAAGAGAGATATCCCCAAGGCATATGGCTCGATGGTCGTATATCT

CAAAAAACGGTCAGAAGCTAGGCGGTTCATCAATGAAGGATTCTTTGTGG

CTGGAGGAGAATCCGGTACTACAAAGGCGTTTGAACGTCGTGAGCGCCCC

AAGCAATGTTACAACTGCCAAGAGATCACGAGCCATAAGGCCTACCAATG

CGACAGACCACAAGTCTGTGGCAGATGCGGGCAAGAAGGCCATCACCACA

GTGCATGCACAGGAATGATTCCGAAATGTATCCCATGCGGCGGCCCCCAT

GAGTCATACAGCAGAAGCTGCCGGAAGCTCTATCCATTACAACATGAATA

GCATATTCCGACTGTTTCAACTGAATGTCAGAAAGCAAGGACCGGTACAT

GATAGTTTAATGAACGACAAGAACATTCAGGATGCAACCGTATTAGCCAT

CCAAGAACCCCAAGCACGGAGGATTCAGGGTAGGCTATTAACAATACCGA

TGGGGCACCACAAATGGGTCAAGATGGTGCCTACGACAGAGAGGGAAGGT

AGATGGGTGATTCGGAGTATGCTTTGGGTGAACAAGGAGGTGGAAGCCGA

GCAGGTGCCGATAGACTCCCCAGACGTGACGGCAGCGGTTGTTCGGCTGC

CAGATCGTCTGGTCTTCACGGCGTCTGTCTACGTGCCGGGGGGAGACGGC

CAAGCTCTACAAGACATATGCGCTAAGCTTCGTAAAGCTATTAAAGAGGT

GAGACAGAGATCGGGGAGAGCGGTAGATCTGGTCATTGCTGGCGACTTTA

ACCGCCACGACCAGATGTGGGGCGGAGACGATATATCAGTGGAGAGACAG

GGCGAGGCGGATCCGATCATTGACTTGATGAACGATTTCATGCTTAGGAG

CCTCCTTCGCCGGGGCACAAAGACATGGCAAAGCGGAGACTACGAAACAA

CCATTGACTTGGTTCTGGCGTCCGAGGAGCTTGCAGACACAAACATCAAA

TGTGCAATACATGGTACGGAGCACGGGTCGGACCATCGCACGATAGAGAC

GGTGTTCGACATTTCAGTCCCGGCACCGAAACAGGAAGAAAGACTTCTGT

TCAAGAATGCACCCTGGAAGGAGATCAACAGTAGGATTGTGGAGACGCTG

AGAGTTAGGCCAGTGGGAAACACGGTGCAGCAGAAAACGGACAGGTTAAT

GTCGGCCGTGCTGGAAGCAGTCCAAGCACTGACACCCAGAGCCAAACCGT

CGCCATATGCAAAGCGGTGGTGGACCCATGACCTCACACAGCTGCGGCAT

ATATACACGTACTGGAGGAATCGAGCCCGAGCCATAAGGCGTGCGGGACA

GAACACTGCAGATCTCGAAGACACGGCAAAGGCTGCGGCGAAGCAATACC

ACGATGCCATCCGACAACGCAAGAACAATTATTGGAAGGAGTTTCTGGCG

GATAACGATAATATCTGGAAAGCAGCCAAGTACATGAAGTCTGGGGATGA

CGCAGCTTTTGGGAAAGTACCGCAGCTCGTCAAAGCAGACGGAACAGCAA

CAACGAGCCATAAAGAGCAAGCCGAAGAACTGCTAGCCAAGTTCTTCCCG

CCATTGCCAGACACCATCGAAGACGAAGGACCACGACAACAGAGAGCCCC

AGTAACGATGCCCGACCTGACTTTGGAAGAAGTGGAACGTCAACTGTGGG

CGACAAAATCATGGAAGGCACCAGGAGAAGATGGACTACCAGCGATCGTC

TGGAAGCAAGTCTGGCCGTCAGTGAAACACGACGTCCTCGCCATCTTCCA

AGCATCACTGGACGAAGGCGTGATACCAAACCAGTGGAGACATGCTCGAA

TCATCCCACTCAAGAAGCCAGGTAAAGATGATTACACGATTGCGAAAGCG

TGGAGACCAATCTCGCTTCTCGCTACGCTGGGTAAGGTGTTAGAGTCGGT

AGTTGCTGAGAGAATCTCCCACGCGGTGGAGACTCACGGACTTCTTCCGA

CCAACCATTTCGGCGCGCGGAAGCAACGATCAGCGGAGCAAGCCCTCGTA

CTCCTGCAAGAACACATCTTCTCAGCATGGCGCTCACGTCACGTTGTGAG

CGTTGTTAGTTTCGATGTTAAAGGCGCATACAACGGCGTGTGCAAAGAGA

GGTTGCTGCAGCGGATGAAGGCGAGAGGGATCCCCGAGGGTCTCTTGCGC

TGGATCGATGCCTTTTGTTCAGAGCGAACTGCAACCATTGTAATTAATGG

CCAAAGTTCCGAAAACCGACCTCTACCACAAGCAGGACTTCCGCAGGGGT

CGCCCTTGTCACCGATACTGTTTCTATTCTTCAATGCAGATCTAGTGCAA

ACTCAGATCGATAAGAATGGTGGGGCTATTGCATTTGTCGATGACTACAC

GGCGTGGGTATCGGGACCGACAGCCCAGAGCAACCAGAGAGGGATTCAAT

CGGTCATTGACAAAGCTCTTGACTGGGAGAGACGCAGCGGTGCGACGTTT

GAGGCGGAAAAAACGGCGATCATACATTTCACCAGGTACACAGGCAGAGT

AGACTCAGAACCATTTACTATTAAGGGTGAGAGAGTCTTTCCGAAGGATC

AGGTCAAGATCCTGGGTGTCATCATGGACTCACGGCTTCACTACAAGCAA

CACATCGCAAGGGCAGCAACCAAGGGTTTAGGAGCTGCGTTGGAACTGAA

ACGACTGAAAGGAATGGCTCCCTCAACAACGAGGCAGCTTTTTACAGCCA

TGGTAGCCCCTGTGGTGGACTACGCCTCCAACGTCTGGATGCATGCGTGC

AAAACAGTATCGGCATATGCGATCCATCGAGTACAAAGAATAGGAGCACA

GACGATTATAGGGTCCTTCACAAGCGTGGCGACAGGAGTGGCCGAAGCTG

AAGCCCACATAGCGACCATTCAAGAAAGGTTCTGGAGACGAGCAAGCAAG

TTATGGGTAGACATACACACGCTGCCGCGGACCAACCCGGTCCGGGAACT

CCTACGAGGAATCAAAGCCTTCAGGCGCTTCATATCTCCACTCAGGCGCA

TCGCGGATGTATGCAGAGAGGTACCGAAGGATACCATGGAAGTCATTCAA

CCATTCACCCTCGCTCCGTGGGAGGCACGCCTACAAGTCATACTGAACAG

TCAAGGAGAGGAAGAAGAAAACAAGATCAAAGAGCTAGCCAAAGCAGGAT

GGGCGGTCAGGATAGCGACAAGCAGCTCCGCACGGAATGATCTAGTCGGC

ATGGGAGTAGCTATCAGAATCCCCATATCCGTGGCAAGAGCCGGCAAGAT

CAGCGAAACCTTTTCGGTCACCCTGGGTACGAGGGAAGAACACAACCCGT

ACACAGCCGAACTGGCAGCGATCGCTCATGGTCTCAACTACCTGCCGGAG

ATGAAGTATCGAGTCATTGTGATCGCGACAAGTAACAAATCGGCTGCACA

AGCTATAGGTAACCCACGCCAGCAGTCAGGCCAAGGGCACATCCGAGAGA

TATATGATGCTATAGAGAAGCTCCGAGGAAACGGCAACAGAGTCAATATC

ATCTGGCTACCACGCGACAGCGAGCTCAAAATCCAGAAGACAGCCAAGAT

GTCAGCTCGTTACGCAACGGAGCCGTACATGACACCACGGAGAGGAATGA

TCAAAGCGAAGACCACAATTCTTAATCGAACGAGGGCAGGTTTGCGAAGC

GAGAGGAAGCTACCAGATGGAGTGGGCAGGCATTCTAGAAGGGTCGACTC

GGCTCTGCCTGGTAAACATACCCGCCTGCTGTACGATCAATTATCATGGA

AAGAGGCCAGCGTGCTGGCACAACTGAGAACCGGAATGGCGCGACTGAAC

GGTTACCTATATCAGATCAGGGCAGCACCGACGGATGAATGTCCGTGCGG

GCGGGCAAAGGAAACGGTAGAGCATTTCCTCTTTCGATGCGTGAAATGGA

CGACACAACGCAAAGAGATGTTTCAGAGTATAAATGAGAAACGCGGCAAC

CTCTCCTTTCATCTGGGAGGTAAGGCAGCATCAGACGGTCAGGAATGGAA

GCCAGACATGGACGCGGTGCTGGCCACGATTAGGTTCGCGATCGCTACAG

GTCGCTTGGAACGGAGATGAcgggaacactactaacctagtaacatttta

tcaacactaaccgataacctatgacgccctcaaataatcaacagcaggca

ttgcttcagccgccgaagataggaaactgaacacttggaggaattgggct

tcaagttgacctgctactactaacaatcacgaggactaagaagaaaagca

atgactagactggaaggaacaaggtgagaattgtatgggcttggcgagag

caaggcaataactagcgaagtgtatgtacctttagcttcagagcccattg

ggcgttggcctaccgggcgtaatagacttgtatt

>Yaret2

tgttggaaaatccgggaatccccttcccttcaggattcagactgttagtc

tgaaagtcctagatttaaagttcaaagtcctagatacaatcatctagaga

tataaacccgagaataactctcagctcaataatgaacaaccaagtttcat

aaaaatacattttcacccagcactactgcgcaatataaacaacaggttat

gagcccgggttagcaaacaccgggtttctcttctgaatcaaagaaccaaa

ctttaacttcaactcaagATGACGTCACGACGAGCCCCACCTTCCACCAT

GCGAACTCGCAACACAATCAGAGTATCGCCTGCATCCGAATCGTCGCAAC

CTGGTGCTTCAGGTCAAGACCACACACCATCCTCAATGTATGACAGTACC

ACGGTCATCTCTGAAAAGAATACTCTTTTCCTTCCGCAAGAGCTCAAGCT

AGGCGGCCCCTCCAATTGGGAGCAATATTGCCCAGCTCAGAAGGCCATTC

TTCGGATCAATGGCCTCGAAGATGCTGTCTTTGGTGATTACCCTCCCGAG

GAACAGCAGACTATGGATCAAAAGATCAGGGCGGCAAAAGCTGCCGTCTC

CATCCGAGGTAACTGCACTGGAGAAGCACTCAGTCAACTGGTCGGGATTG

AGAATCCCCAGGAGATGTTCAACCTTCTCCAGGCCTATTGTATTGGCACG

GGCCCAGTTCTTCTGCAGACAACACTTTACCAGTTCATCCGGATCAAAAC

AAGCTCTTACGAGACAATTCCACAATTTAATGTCGACTTTGAGCGACTCG

TCAAACTTCTCCTCGAGCAGAAAGAACCAATCTCAGATACCCTCAAAAAG

GTTGTTTATCTGACTGCCTGCGAGAACGAGTACCCTGACTGGACTGCCAG

ACAGCGGGCTCTCCTACGCTCTGAGCATCCGCCAACACTACGATCAATGC

AGCAAGATCTCATTGACGAAAACAGGTCATCAGATGATGATGACAGTCAT

GGTACTGCCTCCACGTACTGGGCTCACAGTAAAAAGACTGGAAGGAAGGG

CCCGGCCGCAAGAAAGCAAGAGGCAAGTGGCCGGAGGAGAGGATCTTCAA

AGAACCAGCGAGCCGCGAACAAGTCTAGCCAGGAAACCAATCATCGAGTC

GGCAAGAACAAGGAACATACCTGCACGAACTGCAAGAAGAGAGGACATCA

TGAAAATGATTGTTGGTTCGCCCATAAGGATAAGCGACCCGACTGGGCAG

TCCGTCTCGCCAAGGAACTCATGGAGCATGATCTTCAACGCGACAACTCC

AAAAGTCTCCATATCAAGGAATCAAATCACATGACAGTCGAGCGATCTTT

TCTGATTCTTGAAGACAGCGTCTCAGACGAGCTACCTATGAACAATACGG

AGACGTGTAGGGTCTCCCTTGACAACAATTCTTCACCCACGGCATGGCTC

TTCGACACAGGTTCGTCAGTGCACATCTGCAACGACCAAAGTCTGTTCAC

AGAGCTTCAGCCTGCAACTCGAACAATCCTCGTTACAGGCGGTGGGAAAG

TGTACCCATCCGGTCGAGGGACAGTCAAAATTTGCTTCATAAACAAATGG

GACGATCAGGTCACAATTAACCTCAAAGACACGCTGTTCATCCCTGAGTT

TCCGGTAAATGTCATGTCAGGACTCAGGCTATACAAGAACGGCGGCTGGA

TAGACGGGAACGACATGTACGACCCAACAGGAGATGTCTTTGGTCTCCTC

CGCATAGGAAGGGACGGTCTGTACGTGAACACTGAGGTCGGAAAGACTAC

TGTTTCAAACCAGGCCGTCTCGGAACTCCAGCCAGAACCATGCAATCATC

ACACTGCTTCATCAAACCAATGCCTGAATGTCGACCTTTGGCACCGAAGA

CTCGGCCATGTTAACGCATATCAGGTGTCACAGACTGCCAAAATGACCAG

AGGAATGTACTGTGACGGCCACCACGACCATCAGCCTTTCAATTACTGCC

TCGCCTGCGACATCGCAAAGGCCCTACGATGGACTCCGCGAAATAAACGG

AAACGAGCCTCCAGGGCCGGGTTCATTCATGTTGACACCTTCAAAGTCAA

TCCACCGGGCATTAGAGGCGAACGATGGGGAATGATTGCCACAGATGACA

GGCATAGAATGAGATGGGCCTACACGTTCACGAGGAAGGGGATGGCTTCG

GAACTTCTAGGCCAGCTCATCTCCAAGATACGAACTCAGTATGGCATCCG

AGTGTACGCAATCCAAATGGATGGCGGCTCTGAGCTCTATGGTGCGTCTC

TCAAAAACCTCGAGTACACTCACGGCGTCAAGATCATCAAGACGACACCA

TATACTCCTGAATTTAACGGAGTTGCTGAGCGCTCTAACCGCATCATCTT

TGACAAAGTCAGAGCAACCATGGAATCGGAAAGAATCCCGATAGAGCTCT

GGCCATTGGTCCTGGAAGATATGGTGCGCAAGACAAACGTGACAGCCACC

AGGGCAATTGATGGCCTCACTCCCATGGAAAGCTTTCTCAACGAGGCCTT

CCCCGGACAAGACAACAAGCCGGATCTGTCCGGAGAACGAATCTGCGGCT

CACAAGTCACGATACACATACCGCAGGAACGAAGATTGAGATCACACAAG

TTTGGCCCAAGAGGAGAGGCTGGAATCTACCTATGCATGGAAGGTTCACA

GATCTACACCTGCTGGGTGCCCTCTCGCAGGAAGGGACATCAGATCGTCC

GTTCTGCAAATGTACAATTCTACGAAAAGGTTGGGGAGACAGAACTCCTG

ACCGAAACTGAGCATGATGTTGAGCCGGAAATCCGCAAGAACGGAGCTCC

CATCTCAAGCCGGCCACAGTCCGTCACGGAGCAGACAAAGACTCCAAGGC

CACAAAGGTCTGAAGTGAACAATCTTCAGCATGCTGAGGTGCATAATCCT

CAGCATGGACAGACGACAACATCCATGACATCAGCTAAGCTGCCCGATCT

CCGAAAAGAGGTGATCACTGAGCCCAAAACAATGACTGAAGCGCTTCAAG

GTCCGCAAAGGGAGCATTGGCTCAGAGCAATACACAGTGAGCTGCGCAGT

CTCCTCACGAAAGGAACTTGGCGCATGCTGGACCGCAACCAAGCTCACAA

TAGGCCACTTACCGTCAAATGGGTATTCAAGGTCAAGAAAAACGAGGACG

GCAACCTCGACAAGTTCAAGGCGCGGCTGGTGGTCAGAGGCTTTGAACAA

CAGTTCGGCTTTGACTACAACCAGACCTTTGCCTCTGTTGCCAAAGCGGC

GACTTGGAGAATCCTCCTCACCGTTGCTGCCTGTCTTGACTGGGAGATTG

AGCAGATGGACGTGTCGACGGCGTTCCTGGAAGGGGACCTCGAGGAAGAA

GTCTTCATCGAAATGCCGGAAGGGCTTGTAGAGTATTTCGACCAACACCC

AGAAGACCGTCCGCCAGGATTCTCAACCGAGAAGATCTGCAAACTGATCA

AATCCCTCTATGGACTCAAACAGGCGCCTCGGCAATGGCAAAAGAAGCTG

AAGGAGGCCTTGGAATCCCTTGACTTTCGACAAGTAACGTCTGACACGGC

CGTATATCACAATCCCACAACGGGAGTGATCATCATCACATATGTGGATG

ACTTCCTCATCATGGGCAGCAACAAGGAAGCAATCCAAGGGTACAAAGCC

CGCCTGGGAGAGATCTTCACAATGACTGATCTCGGTCCCGCCAGCCATTT

TCTTGGAGTAAGAATAACCCGAGACAGGAACTCAAGGCTGATCTACCTTT

CCCAGGATGCATATTTTACCAGAATACTGAAGAAATTCGGCTTAGAGGAT

TGTCGACCGGTCAAGACCCCGATGGAGCGAAATTCACTCTCGACACTGCA

ACCAAGAGACAATGGCGACTCGGCTTCTCCAGAAGAACGAGAAGACTATA

GCTCGAAAACCGGCTCGCTGATGTATGGAATGACCCAAACCAGACCCGAT

TTAGCTTTCCTACTCTCGGTACTCTCAAGATACATGTCGAATCCATCACC

AACACATTCACGAATGATCAAAAGAGGTCTCCGCTATCTACAGCAGACAA

GAGACCACGGCCTGGTGCTTGGGGGCGTCAAGAAAGATCCTGAATCCGCT

TGGAGCATCACAGCTTGGGCCGACTCGGATTGGAAAGGCGACACTGTTAC

GGGAAGATCGACGTTTGGATGGCTTGTTCAACTTGAAGGATCTACAGTCT

CATGGAGGGCCAAACGGCACGAAACAGTGGCACTATCGACTACCGAGGCT

GAATATACAGCACTCTCCCAGTGTGCCAGAGAATTGGCCTGGACTAGGAA

CCTCTTCTCTGAACTGCTCCTTCCGCTACATATGCCTATCCCACTGAACG

GCGACAACCAGGGTTCCCTAAAGCTATGCAGAAACCCTGAGCTTCACCAA

CGGACAAAGCACATTCCGTTAACCGAGCACCATATCCGAGAAGAAGTTGA

AGCCGGGAATATTGATGTGCAATACGTCAGCACACATGAGCAGGTAGCAG

ACGGACTCACTAAACCATTGAACGCCGTCAGCCACGGTCACTTTCTAGAA

GCAATTCGAGTCAGTGCATGTCCGATCGAGGAAGCAGGTCGTATTATTTG

GAGTACTGAAGTCCACGATGACTCTGTGCCCTGAagacaaggtggatggg

ggcgtgttggaaaatccgggaatccccttcccttcaggattcagactgtt

agtctgaaagtcctagatttaaagttcaaagtcctagatacaatcatcta

gagatataaacccgagaataactctcagctcaataatgaacaaccaagtt

tcataaaaatacattttcacccagcactactgcgcaatataaacaaca

>MGR583-like LINE element

caccttcctttgtcatcacactttcttggcatacaagtgttaagaaagtg

gccctgtctacacgtaaatccttaacaagtgcacaacaaacattccaatc

tgcgctttgcgcagtcgcaagccgcggcgatggcttcgtcttcgacgaga

tcgcccgctgccttggatcccttcgatctggggatccacactcccatcaa

cctgaaccggggagccgtgcagccttcttacagaacagtccggtagtcgt

caacggcactatcggtgtttcacctgctcccggccggatggctcagacac

catcctcaccactcaacgccgccgccaccaccaccacagctacggaagga

gctgaacgtgccagtcccgtatccacttttgaacaacgaccaatctctat

cgttgaatcagccaacgatctcgcacgagaacacgcggaagagtacaaca

ccaagctgatggtgttccgggccttctgcgccaagtttgaagaagcggcc

cagcaattcgtcaccggaccgcaacgacgtttcgctcagcaatttgccga

tgatttcctcggtttttggaaacgagagctctcctgctccggacctgtga

cccccaagcctacatacagcagcgtggtcactgccgcgcctcccactgac

catgaccgtctgacctagagacaacaacaacaacatagaggccgacaaac

agatccacctcatcgccaagggcaacagacgaccatcgccccaccccgac

aagacctccgcgtctttatccgtctagaagccggagctccggccagggcc

cacagtagctatgccatccggactctgattcgggagaagctcggcgctgt

ctcagacaagatccggcaggtgttccaggtcagatcgggatgggccgttc

tggctgccgactcggcaacacgcgaccttctggtagaaaagcaagctgag

tgggccgctgaactgggagctacagcagtagaaacgaacaaggaatggtt

cacctatgtggtctcagacgttcctacaagactgtctgacttttacggaa

atgaggtggatagtgacagcgtcgtcagcgacgagatagaaatctagacg

gggctcaagtccattgacgtacgtaccggaagacaattctcggataaccc

cttgaccaaggccctacttgtgtcctttctgaagcccacaaaaagattct

ggtctcttttcggcagtagcgcagctagacttgttgacaaaaccgaccga

cctagacaatgtgagaagtgctggggccaccattttgcccgcaactgtca

cagacagccagtttgccgacgctgtggtgaggctggccatcttgtagacg

attgcatcgcaccagaacagtgcgtcaactgcttgggccctcaccaggcc

aactttcgtaggtgcccagctcggccaaagacagtgcacggcgtgctccg

ccgacttactaaagagcaacgaaaacatgtcagcgccgttggtgcggaga

cataccgacaacgacaccaagaaccacagtcgggatcgcatcaggaagcc

cagcagggcatggccgagcgacaaaacgaggatgtcacatcacaagaacg

accgaacgctcgtgctccaagcccagctgtatcaggagcaccttcgtgca

tcatggtggccacgacctctcatgctggctatgaagcagaggaagaaccc

gaacagcccagaccaggctcaccgcgaaagcgccgaatagttctcattaa

tcgctcgcATGGCCAGGAATAGATATTCTCAAGCACGAAAGAACGACAGG

AAGCCGCTCAGGATCTTCCAGGCCAACGTCGGCAAGATCCCTCCGGCCCA

CGACTGCGCCCTGGCGCTGGCCGACTCGGAACAATATGACGTTGTGCTCT

TGCAAGAGCCGTGGACGGCTCACACAGAGACCCGCACTTTAACTAAGACT

CACCCTGCATACGACACATTTACGCCGGTCGACATGTGGAACAGCAACGA

CACTCGGCCTAGAGTGATGACATATGTCCGACGAGACCCAAGACTTCTTG

CTGACCAGATTCGGCCTTTTCAGACTCGTGATATCCTTTGGCTCACAATC

AACGACCTGACGATCGTCAACTTCTATCGTCAGAACGACGAGAGGGACGC

CCTAGACACGCTATTTCAATGGTCTGTCCCGGAGCGTTGCCTCGTAGCTG

GCGATTTCAATGCCAGACATCGTAGCTGGCAAACAGGCCAAACTACGAAC

CGGGGCCAAGAGATAGCAGGATGGGTGTCTGAGAATGACCTCAGCCTTCT

CAACACTTTAGACATCCCAACAAACCCGTACGGCAACACAATCGATCTTG

CCTTTACTAACCTACCACTTGCTGAAGCTGTCGTCGAGGACCATCTCGCC

ACCAGCTCTGACCACTTCACGCTTAGCCTGACCTTCTCAGACGTCAGATC

GACTCCGGTGCAGCCAGGCAAGATTCGAGTGACGACGGAAGACGAGCTTA

AACGATTCGTCGAGATCGTAGAGCTTGGCGCTACAGGAATCCCCCTCACA

GATTCGACCCCCGAGGAGCTGGACGAGCTTGCGTCTTCACTTGTGAGTCT

ACTAACATCAGCAGCAAAAGCGTCTGGGAGGCCCGCACGGAAAGGCGGAC

GCCCGGCTCCCTGGTGGACGGAGGAGTGCGCCGACGCCGCGGCTGCTTTT

CGAGCCATCAGAAGAAGCTACCCACTCGGCTTCAACCAGGACGTTCAGAT

CGCCAAGAGAGGCTTTCATCGTGTGGTCCGTCGGGCCAAGAGGCGCTATT

GGCGTAACCTCATCGACGGCTTCTCCAGCAGTAGCGATGTTTTCAAAGCT

GTCCGGTGGCTAAAGTCCCCAGGAGCCTTCCAGCCGCCACCTCTACAGAT

CGATAACGTCGTATACGAAAGCCAGATGGATAAAGCCAACGCACTCCGAC

AGGCCACGCTTGAACGAAGAACTGCGGAGGACGACATCGCAAACGCATGG

ACGCCAGTATTCCCACCTAGATCGATCCCATTTTCTCCCGGAATCTCTCT

GGAGGAGGCGCAATATGCGACTTGTCACACAGGCAATACATCCCCAGGGT

CAGACAACATCACAGTCAAACTTCTTGAAGCAGTATGGCATGCCATCGGT

ACACACGTCCGTCGTCTCTTCGAAAGATGCCTTACCATAGGCCATCAGCC

AAAACCATTCAAGGAGGCGGAGGTGGTCATGATCACGAAACCGGGACGGA

GAGACCTTACCGAACCACGAGCATGGCGACCCATCTCACTGCTCTTCTGT

CTTGGTAAGGGACTAGAACGACTGATCGCGCGCCGCCTAGCCTGGGCAGC

CGTTCACTACAGCGTCCTTCATCCGCAGCAAGCTGGGGCGCTCCCCAAGA

GGTCGGCAACAGACTTGGTAACTGCTCTGGTCCATGATATTGAAGAAGCG

TTTGCACGCAAGAAGGTGGCGACTATAGTCACAATGGACGTCCAAGGTGC

TTTCGATACCGTCATGTGCAATAGGCTCGTCCTGCGTCTTCGCGAACAGG

GCTGGCCTAATCATCTGGCTCGCTGGGCTGGCTCCTTCATGAGCGGCCGG

TCGGCGCGTGTCAGGTACCAAGACACTGTCACGCCCTCTTCTCCCCTTCA

GTGCGGCCTCCCTCAGGGGTCGCCGGCATCGCCGATCCTCTTTCTGCTCT

ACACTGAGCCAATCTATCGACTGGGCAATCCCCGGGGTCGCTTCGGCTAT

GCAGACGACACGGCCATCCTGTCCATAGGCGACACAGTAGACGAGACTTC

TGCCATGGCATCTAGTTCCATCGCCGAGATGGTGCGATGGGGCGCGGAGA

ATGGCGTGTCCTTCGACACGAAGAAGACCGAAGTCATGCATTTCTCCCGC

AGCAAACGCAGGACTGCGCCGGCAGTACGCCACGGCGATGTCGAGAAGCA

CCCTGAACCAGCTCTGCGCTGGCTAGGCATCTGGCTGGACAGCAGGCTAT

CTTTCCGACTTCATGTCGAAAAGTGGGCGGCCAAAGCAAAGGCAGTGGCT

TATCATTTGCGAGGGCTCAACAACACGGTACACGGCCCTCTACCGAGCGC

CGTTCGAAGTGCTGTCAGAGCATGTGTCGAGCCAGTGCTACTTCACGGCT

TGGAGGCGTGGTATCCAGGCAGCACTAGGCCGCGATGGAATCAGCCCACA

AAGGAACTACCGTCGAGCAATCAGCATCTCATACAAATAATGAACAAAGC

CATGAATCAGGCCATGAGAGCGATACTACCTGTCTGGAAGACGACTCCCA

CCGCCATCCTACATAGGGAAAGCGGTATACCACCAATTGACCAATTGCTC

GAAGCAAAGCGACTAAGATTCGCCGCACGACTCAAATCGCTAGACGAGGC

TCACCCTCTGGCGGGCCGAACGCGCCCGCGTCGACCGCCTGACCGACCTA

CCTACCACGACCTCATCAAACGAAGATACCAAATACAGACAAAAAGTGTC

TTCAGGACACGTCTCCGACGCACCGACGAGCTGCTTGCGCCCTGCGAGCG

GCCAAAACTCGTCCAGCGATGCTTCCATCAAGAACAGATGCCTCCACTCC

AGATGGCGTCAAAAGAGAAATCGACCGGCGCCTTCCTCCACTGGGTTGAG

AGACTCGACCCGCTCACCTTGGTCGTATACTCAGATGGCTCTCTATCCTC

GGAAGGAGCTGCAAGCTACGGCTTCACCATTCATCAGAACAATGTCCCCA

TCTTCGATGGATCAGGTCGCCTTGGGCCTGCCGAGGTCTTCGACGCTGAA

GCTACTGGGGCATTAGAGGGCCTCAAAGCTGCCCTGAACCTGCAAGGGTC

AGCAGCGCAAACCATTTTCATCTGTCTAGATAACCTCGCGGCCGCCACGT

GCCTGCGAGGCACGCCCTCCGACTCCTCTCAAGACGTCTTTCTCGAGTTT

CAGGCTCTAGCGGCATCGCACGGAGCCACACAAGTACTCTGGGTCCCAGG

ACACTCCTCTATCCCTGGCAATGAACAGGCCGATAAACTGGCAAAAGCAG

CGTCATCACTCCTCGAACCTGAAGGTGCTCAACCAACGCTGGCTTACCTA

CGACGGATCGCGAGACAAAAACCAAAAGAAGCATTCGAGACGTGGTGGTC

TACCTCCGCTCCTGAGCAATACAAGAGACTCAACCTCAAGGCGACTACAG

GCTGCCCACCAGAGCTGACGCTCCCGCGCGCAGCCTTACACCACTTGCTG

GCAGCGAGGTCCCTCCACGGGGACTTCGCCGCGCATCACGAGAGGTTCGA

CCACGGCGATGCGCGCCTGGTCTGCTCATGCGGCCGACGCAAAGCACCGG

ATCATATCTTCTACTGCAGAAAGGTACCGCAGCGTCATCGGATGAGGCTG

GCACCCTCACCGAATGCAGCAGTTAATCTAGCAATAGGAAGAGACTTTAC

CAAGTTTATCGATCTGTCTAAAGACAGCGCGTTCTTTGGGAAAATTTGCC

CTCGCTACTAGgcgcccacgacttgagaagcactcgcgactctctttctt

tctctaccatcgacgtatttccttgttacctttcctttctctctcatata

ctcacggccagatgaagggcgggccaacgcccctcatttggccgcgacta

ccttgaggcagtttcaagctgccgcatgcacaggctgatctcagcctccc

tgatatcaggctccaaaagcggctgtaatgtcccggctgcgccttgtcat

cccgctgacgggtaacaagctcgataggatgcgccatttgtgccgatgag

atgctggatagagtgtgtttggtctactaacgcggttgcaaaaattggaa

tctgagggaagactaatttcgcgtcgcgaatgactaatcgcccttgtagc

caatggtatttctgtaattagcttatagatagatccccaattctctagct

cagcacgtcctgtgccccagcaggggactctcggggcctacggccccccg

gacgaaaaatatacataacataacataacata

>Gollum

TGTCGCACTGAGCGTGCTTAAAGGGTTAGAGTGAATATCATGAGCAGCAG

CAAAATGTGTTGATTGATTGTTCTTCGTTATCTCTTGTGCGGGGAATTCC

ATCCCGCAGAGTTACTTAAAGCATATAGCTAGATTACTAATGCCTACCCT

GCGACA

>Folyt2

gatggatttaataccgtatcattaaataaaatgcgctttttgtattaaat

atttaaaacaggcgcgttttatatgtattatattccattccctattagga

ttagctccttccagaactggcttagtgtataggatcattataaccttaat

ccctgttccgacctccttttctccacagccttcaacagcctatcgctgta

tcccctgaaacttcccaatctgacttcccactaccttcagacccttcgtg

cttcttctacaATGCCTCGAACCTCTGGAAGGCCAGCCGACCACCAGGTC

CACCGAGAATTTATCACGATTTCCTCGGACGCTAGCTCAGGGTTCAAGAA

CTCACGAGTCAAGTGCAAGCACTGTGGCCACGAGACAGCCAAAGGCACTA

CTCGTCAGAAGAAGCACTTACTTCGCCATTGCCCCAATTACAGAGGGCAG

CAACAGTCTGAGCAGTCTCAACTTACTCAGCACTTTCCTGTGGTAGACAA

GACTTTTAAGCAGATGCTTGATGAGCTTGCTGCTAGAGCAATTTTCGCTG

ATGGCCGTCCATTTAACCTATTCGAGTCCAAACGAATGCGAATTCTGTTG

AACAAGCTGAATACTGCTTGGCAACCACCGTCTCGTCGCCGAGTGCAGCG

GCTGCTAGCCCCGACCTATTCTGAGTACCGCAACCAAGTCCAAGATATCC

TCGACCAGACAGAACGTATCAACGTGATCTTTGACGGCTCAGACAATATC

ACGAGTCATCGAATTATCAATATCTCGATACAGGTGGCAAATGGTCTGGC

GTTCTATTGGAAGACGTTTGATACGGGACAGATTCAACACACGGCTGAGC

ACTACATCGATCTTTTGTATCCTGAGCTGGAGATAATTTGCAAGGGTAAC

TTCTTGCGCATTAACTCGTTTTTGCACAGATACGGATAGTGTGATGAGGA

AAGCTCATGTGCATTTGGCTGCAAGAAAGGAATTCCAACATTGCTTCTTT

TCGCTCTGCGATTCACATGGGCTACAGCTACTCATCAAGGACATCCTAGA

GCAGCCGTTCTTTGAGGAGGCATTCAAAAGCGCTACATTAATTGTCACCT

TCTTCAAGAAGTCGAAACTGCAGTTGGCTCGATTGAGAGAAGCACAAAAG

GCAGCTTGGGGCCACCATAAGGCGTTTTTATCTGCgtgcgtctttcagtt

tttctgactcttagctctaactaaccgctcgctttagcgcaatcacacgc

tggggcagTCAATTCAATGCTTTATGGTCAGTTTTACACTGTAAAGAACC

TCTCCAAACATACGCTCGCCGCCCTGACGTGAGGGCAGAGCTAGCCTCCG

GGTCTCTCGAGCTTCTTCCGAGGGTGCTGGAGTCTGTCAATAACCCTCAC

TTCTGGATACGCCTAGAGACTGTTTTGGCTATAATTAAGCCTGTTAGCAG

TCGTCAGCATGCCTCAGAAGCCGATCGGGCTCACATCGGCCATGTGATTC

CTCGTTGGCTGGAGATTAAAGCAGAATGGAAAGCACTTGACGATTCTCAG

CAACATAAAGACGTGAATTTCAGCGAGCTGTATTCAGTATGGTTAAACCG

TATGGATAAACAGACATATGATATTCATTATGCAGGATTTGCATTAAGGC

CTGACACAGTCGGGACTAAGCTTGAAGAACAGCTGATGATGAAAGTGCTT

CAGTTCTTTAAGTCAGCAGTCAATCCTGCTGACCACCTTCATATTGTTCG

AGAATTTAACCACTTCCGAGCACAATCGGGGGGCCAGTTTGCTGCCGGGG

GCTTGGTCTATTCAAAAGAATGGACGCCATTAGATGCTTGGATGCTTCTT

GATAACCAGGGCAGCAAGCTGGCTGCACTTGCAGTCCGAATCTTTGGAAC

CATAGCCAATTCAGTTCCCTCAGAGAGATCATTCTCGGCGGTTAACTTCC

TCCACAGCAAGGCACGCAACAGGCTCACACCAGCCAACGCTGACAAGTTG

GCCTTCATCTACATGAACGAACGGGTGCTAGAGAGGATAACGCAGTCTCA

GAATCAACCTCTTGGTCATCGCGATGAGGTTGATTCTACAGTGGTTAGCT

GGGAAGATTTGACAGAAGACGGTTGGCTTACTCTCGAAGATACGTATATG

GAAATCCATTGCGCATCAAGTCTGGAAGTTGACGCTGTTATCGGCGAGTT

CACTCACCAACCCGCCTCCGACGGAGAGGAGACGATTGTGGAAAAATTTG

AGGTCCAAGATGGGTCAGAGAGTGAAGGAACGGGGGGGAATTGAcctaat

ttggcttggttatctgtagtatatgactgttgggcggcttcatacatggt

agcaatattgcaataaaaaacgcgttttttattcggaataaaatacagtt

tcattataataaaaaacagtttaatacacacttgtattaaacacgtgccc

atcccta

>FoHelitron

tttattcagccgaaggctgactctacattaattcataagttttcccgagt

aatgagggtttggtgcagacagggataaataagtttctcggaatctgatt

ggtccagattttgtatggccagccgcgaaagaacgcgcaactggctgatg

gcaccaaaagagtggggccaatcggtgctggcccctgaaaggctcacgcg

gagtgccggcagcttgcgtccatttggaattgtttgtttactcagtactg

tgccagtaccacgcgttcaattcatctccccaattcgacaggtctcgata

tggcctcgttgattgagtaATGGATATCGGTCTCAACCCCGAAGAGCTCT

CTACTGATTTACTCTTCGTTGTTTGTGCTCGTTGTGGGGAGGAGAGACCG

GAGAGTGCCTTCAGAGCAAAGAGGCAGTCTGCTGGGCAAACTAAGCAATG

CATAGACTGCCGTAACCAGCGCGTTTCTCATGTAAGTTCTGCCTTCCTCG

TCTATAAGTCTCTTAACCCTCATCAAATGAAGCATTCTAGATCCAAAGTC

GTGCTCCAGACGCTGCGGAACATTGCACTTCGTCCATCCTCACCTGGCAG

ACCTGCCACGAAGAGAACCGAAGGAGATGCTGGCCTATCGCCTCCCAACG

AGAGATCCGGAACCCAGCCTACATCGCCAGAGAAGCTACGGCAACTTCAT

ACAGCTAGGAGTTTATTCGGAGAGTCAATTAGCCAGCCGCACGTAGTGCT

AGGGACGCCAATTCCACCAACCCAACAAAGCTCGCGGCTCTTCCGGGCTC

TAGCTCCGTCACCGCCTGTGCAGCCAACGACCCATCCACTCGTCTCACAT

TCTGATCCATCTACTCTTCGAAGCAGCACACCTGGTGTGGATTACCCTTA

CTTGGCCACCAGGTTCCACAAGGGGGGGAAGGACTCGCAAGATGATTCCT

TGAAGGCTGGGGCGAGGGCCAAGCTGGCTGTTATCCAGCGCGACCATCGT

TCACGACGCCGTGCAGGGGAGACTGTGTCACTGACTCCCACAATATCTCA

ACTTGGCTTCTTGGAAGATTTTGAGGGTCCTGGAGAAGgtatccttgttc

tgtggccccagtagtttgttcaggctaaccacggaacgtagATGGGAGTC

AAGATCAACTACGACCGAGTGGGTTTCTAGATGGGGATGGGATAATCAAG

GAAGGGGATGACAGGGGACAGTTCTCTGAATCTGATATTGACGCCGATGA

CTATTTCAACTTGCTGCTTTCACCTGATCGACCACGGAGGTACCTCAAAC

AATTAGGGGTAGAGTCTGATTCCGATCTAGGGGACGAAGACGAGAACGAC

GATAATGATTGCGTGGATGGAAGCCCTCTTCGCCATCGCTTGCGGCAGCC

TTCCGCACAGCTAAGGCGTGGTCGCCGTGGTCCTGCCCCTGGTACAGGAG

GGCGGCCAAGAAAATCTCGAAGGCAAACTCGATCGTCGATGCCGCCACGG

CGTATTCGGAGTCCAGTGATAATTCCACCTGAAGAATCGGCGGTCTTTCA

TGCGCAAGATCCGGTGTGGAATGGCGACCTTGAGGCTTGCGCCTTGACTG

GTCGCGATAAGGCAATCCTCCGCGAGTTCTGGACAAAGCTGGACAATGAC

CAAATGCAGTTTTGCGGTCGATGCCGGGAGTGTTGGTTCCAGATGAAGAT

CGATTGCGATGGCATTTGTGCGCGTTGTTATCGAAAAGATGAGAAACGCC

ACCCCGACGAGCCGTACTTCTTCTCTGCGGATAATCAGCTCGACTTTGGC

CCTGTACCGGCCCGGTTGCCCCAGCTTACGCCTACCGAAGAGTCTTTGAT

TGCTCGTGTTCACGTCCACGTGAACATTATGCTTGTGCGAGGGCAGCAGT

ACAAGTATCGGGGGCACGTAGTTCACTTTCTCCGTGAGGTTGGCTTAGTG

TACAACCAGCTCCCGCTTCTGCCGCAGGAGTTGAACATTGTATTACTACG

TCCTGCCAATACGTCGTCCCACGCAATTCTTAGTCGGCAATTCACCCGCC

AGTTCCGTGTCCGCCGCCAGCCGGTTGTCATATGGCTAGACTACCTCCGG

CGCCATCATCCTGGGTATCGATGCGTCGTCATCGACGAAGAGAGGCTAAA

TCAATTGCCCCAAGATGGCAATGTCCTGGATGCCATCCCCCAGAGTCAGG

TGGAGGCTGCGGATGTTGGACCCGAGGAAGATCAGGAGGCAGAGCCTGAC

CTGGAGGACGAGGCTGCAGTGCCAGACATGTTGGCAAAGGACACGGAGCT

CGATGCTCTGCGGTCTATTCTCGCCGGAGAGTCGGAAGCTGATTCAGAGC

TTTCCACAAGCTTCCAGGCGCAGGCGCAACACGAGCTGCAGCTCCCGAAT

ATACGACACACACCCATTAATGAGTTCAATCGCTCTCATGCCCTACTCTC

CTTGGCGTTTCCCTGCCTCTTTCCTGACGGTAGAGCCGACTTTGTTGAAC

CTCGATTGCGCTCCATTGATTACAAGGATTACGTCGAGCACGCGATGCGC

TGGCACGACGGGCGTTTTGCACGCCACCCGACCTTCCGCTTCGTCGCCTT

CAACACGCTAATGCGGTCACAAGCACGTTCGCGGTCCAGATTCTTCGTGA

AGCAACATGATGGGAGACAGCAGCCGCTGACGCGAGAGCAACTTATTCAG

GCGCTGGAACACAGCGAGGACCCCGAGGCGCAGGCGCTGATCAACTCGAT

CACAAGGCATGCGGTGTCTATTCGCGGTACGCGTCCATTCTGGAACAAAA

AGAGGCAGGACCTCGAGGCCTATGCCTATAACCTTGGTTGTCCTGGTGCA

TTCATCACGTTTAGCCCGGCAGATTTACACTGGCGGAGTCTCTACCAGCA

CATGCCCCAGTATGACGACTGGCTAGCCGCCACCGAGCCGGAGAGGATGG

CTCTATCGCGCCGCCTATTGCGGCAGAACCCTCACATTGCTGCTTTCCAC

TTCTACCGCCGATACACCCTCTTTCGGGATATCGTGCTAAGTAAGAAGTT

CAGCATCACAGATTACTGGGATCGGTACGAATGGCAAGGCCGTGGTAGCC

CACACAACCACGGCCTGTACTGGATGGATAATTGTCCAGGGGCCGACATG

GAGGACGAAGCGGCTCGTGATGTATTTGCACGCACATGGGGATTCCACGT

CACTGCCATTAACCCTGAGCCGAGTAGGACTATGCCTCAGGGTGAGGGTA

ATCCCCTGAGCGTGGATCCCCTGAGCGTGGATCCCCTGAGCATAGAGATG

ACATTCCTGCGGCTCTCACAAATCGTCAACCGCTGCCAGCGCCACAAGTG

CAATACCACGTACTGCTTGCGCGTGAGGAAGAGAACCGGTGATCTGGCGA

GGGACATGGAAGGTGCCGCTGCGGATATCGAGGCGGCAAACGTTGCCAAT

CCAGAGAGGGAGTGTCGTTTTGACTTCCCTCGTGCCTTGCGGGAGCTGGC

CGCAGTGATCAGGAAGGAAGGCAGGTCGTACTATGTCTTTGAGGCGGCCC

GGAATGACAGCCTCATGAATAACTTCAATCCTGCGATTATCCTAGGCTGG

CTAGCCAATATCGACATATCTCCTTGCACCAGCTTACAGGCGGTTATTAC

GTACGCTGCGAAGTATTGCAGTAAATCTGAGAAGAAGACCGAGTCTTTCG

CCAGGCTTGCAGACCAGGTCTTGCCTCACACATCGCACGTTCAGCCCCTG

TTGTCCTTCTCCTCTCGCCTGATGAACAAGCTGATTGCCGAGAGAGATTA

CTCGGCGCAGGAGATTTCCCATCTGCTGCTTAACATTCCGCTGCAAGAAG

GCACCCGTATGGTTGTCACCGTGGACTGCCGTCCGTTGGAGCGACAGGCA

CGTTCGTATCGTGTGGACGGAGATGTCAACGAGGCCATCGGCAGCTACAG

GAAATATCTAGAGAGAAAGGACCAGCATGAGGATATAACCTATCTCGAAT

ACCTGCAATCGTACAATCTCAACACGTGGAGGAGACTCGCTGCTCACGCG

AAGAAGAGAGTCCTGTCTTACTTCCCTCGATACAAGTCTATGGAGGCCTC

TTCCCAGTTTAACGACTTCTGCCGTGTTAAATTGATGATGGTTCATCCAC

ATCGCTCTCCAGAAGAGTTGCTCGTTGTGGGCGGGCAGCGGTTCGATTCC

TTCACGGCTGCGTACATGCACTGCAGACAGCACCATGATACCCATGCGGA

CGATCATTATGGGGAACCAGATACAGATGAATTGACGGCAGAGGAGGATG

AATTTGAGCTTGAGATCCATGAGGAGCCCATCGCGGAGGAGGACTGGCAT

GAACTCGCCCGCATGCTACCTGACCGCCCACTGGAGGAAGAGGACATCGA

CATCCTCGGCCGCCGAGACATTGACATCAATTACGATTGGACCCCTCGTG

TTGGGCGGTATGCCGATGATGGCATTCTCAACAGCGACTACTGGAAGCAA

CGCAAAGCGGAAAACACCCTTGACCTTGATGTGGATGATCAGCCCTTGGA

GGCTCGCGATTCCCTAAATCCAGAGCAGCGCATAGTATACGATACGGTGA

TGGGCCACTTCCTGACCCAGGATCCCTCTCAGTTGCTACTCCATGTGGAT

GGCGGAGGCGGCACTGGCAAGTCATACCTCATTAACCTGCTCTCCGCGCA

CCTCCAATCCGCGACAGGGGGGAGGGGGACACCTGTTTGGCGTGCTGCGC

CAACGGGCGTCGCAGGAAACCAGATATCGGGCACTACCTTGCACTCCCTG

TTACACCTCCCAATCAATAAGGACTTCAAGCCTCTATCTCCCGTTGATAA

GACCCAGCTCCAGAAGAAGCTGAAGGATATCAAGTACCTCATCATCGATG

AGAAGAGCATGCTAAGACTGCGTCAGCTATCGTGGATCGATGACCGTCTC

CGCGAGGCGTTCCCGAACAGGAATGAGGAGTTCTTTGGCGGCCTGAACAT

CCTCTTGGTTGGTGACTTCTTCCAGCTTCCCCCTGTGCTACAGAAGCCGC

TTTGTTACGACAAAGAGGTGCAGGGAGTAGAGATCAAGGGCAGGAACGCA

TATAGGCGCTTCGATAAATCAGTATTCTTGAAGGTTGTTCAGAGGCAGCG

CGGCGACGACCAGGAGGCGTTTCGCACAGCTCTCGGGGAACTGCGGCTGC

TCCAACTATCTATGGAGTCTTGGAAGCTCCTGTCCACCCGCGTGCAGGCA

AAACTAGACGATCGAGAGGTCGCGAGGTTTTCCAGCGCCCTGCGAGTATA

CGCTACCAAAGATAGGGTGAACGAGTATAACCACTATCACCTCGACCGCC

TCGGCCGGCCAGTCGTCCAGGTCAAAGCTAAGAACGTCGGCCCTGGTGCG

GCTGCTGCCCCTGATGACAAGGCGGGCAACCTTGCGAAGCAGATCCCTAT

ATGCATTGGCGCTCGTCTGATGCTGACGTCTAACCTTTGGCAGCCCGTTG

GCCTCTGCAACGGCGCTCGCGGTACGGTTTACGACATCGGCTGGGCACCC

GGGGCTGACCCCATCCAAGATCCTCCTTGTGTTATCATGATGGAGTTTGA

CAAGTACAACGGACCGGTGTTCCTGACCACCCCCGATGGCAAAAAGATTG

TCCCAATTCTCCCAGTTGAGAGAGACTTTCTCATCGGAGCCACTCTCTGC

GCTCGCACGCAGTTTCCCCTGATCGTATGCTACGCTATTACCGTGCACAA

GTCACAAAGCATCACAGAAGATATGATCGTGACGGATCTCTCCTGCCGGG

ACTTTCAGACCGGTTTAAGCTACGTGGCTGTCTCTCGTGTAAAAACGCTT

GAGGGTTTGATGTTAGATGCGCCATTTGATCGTAATCACCTGGTCTACGG

GTCTCCCCCGGATGGCATGAAGATGAAGATGAGGGATCAGGAGCTTAGGA

AACGACAGGTTCTTACACGGAGTCCTTACATGATATATAATACCAAGAAT

GGCCATGGCACTGGATCAGTACGTAGTAGTAGTAATAGCTTCCCGGCCCG

CGGACACACGGCCTTCGGCTGAggctaccccatgcgccccatcgccgctc

Caatgtgcatggggtggcgtagtatttta

>Hop2

atcccgttatgtaagaggcatatgtggcctaattgcaaagcggccataca

ccgcagattaaccccatatttagcaatcaaccgcttctacggtattaatt

cttattgccgcgccttgcttctgctataATGGATCCAATTGGTGACTCTC

ATGGCTTCCCCAGGGATGTTCTTCCTCCTGAAGGCGAATTTAGCTCACGA

GAGGAACTAGTAAGAGCAATTAACGCTTGGGCAGCGCCACGAGGCTACGC

CTTTATCTCCCAAAGGTCTTGTAAGACTACCAATGGGAGAGTTAGGGTTA

CCTTTATATGTGACCGCGGAGGAGGGCGTGCCCCTTCTACATCCCCTAAG

AAACGGATACGCAAGACTGCATCACGTCGTACGGGATGCCTCTTCTCTGT

GATAGCAAAGGAGAGCCTATGCGGGACTCAATGGAGCCTGAGGCATCGTC

CCGGCCCTGGCTTCGATCAACACAATCATGAACCAAGTTTCCACCAAGTG

GCACATCCAGTACATCGTCAATTGTCAAGCTCAGATGAATCAACAGTCCA

CCAGCTCGCCAACGCCGGTATTGCGCCTAAGGAGATCAGATCCTATTTGC

GCACGACTTCAGATACACTTGCCACACAGCAAGATATCTATAACTGCATT

GCACGAGGCAAACGGGATCTTGCCAAGGGTCAAAGCAACATACATGCCCT

TGCTGATCAGCTCAATAGCAGCGCTCGCAATAGTCCAGACCATTTGGACT

GTTAGTCCAGACTGTAGATCAGGGCAGGGAATACGGACGCCCGGTGTGGA

CGCTAAGAACGCTAGGTTGTTGGTATGGTGTTACAAAACCAGGGCACCCA

TGATTGTTGCTGTACCAACCATTTTATTTTGGATATTTCCGATCAATTTA

TTGGTGCGATCCGATCAACACGGCTCGTTTCAAGGGACGCATCATCCTAG

CCGTCAATCCACGAACGCCCGTCGTGGATTGATTGTTGATTTTGGATTGC

AGTCTGGACTAATAGTCCAAATGGTCTGGACTATTGCGAGCGCTGCTCAA

TAGCGAGGGCTTCTGGAGCGAGATTCGCCTCGACGAGGGTGGCAGAGTTA

CAGCAGTATTATTTGCACATCCGCAGTCACTGGAATACCTCAAATTATAT

CCTGAAGTGCTTCTATTGGATTGTACGTATAAGACCAATAAGTACAAGAT

GCCCCTTCTCGATATAGTTGGTATTGATGCTTGTCAACGATCCTTCTGTA

TTGCATTTGCCTTCCTTAGCGGCGAGGAAGAGAATGACTTCATCTGGGCT

CTTGAACGGTTGAGGCATATGTATGAGCTTCACGGGGTAGCTATTCCTTC

TGTAATACTCACAGATCGCTGCCTTGCTTGTATAAATGCCATTTCCTCTT

CTTCTTGCTTCCCTGAGCCAGCCTTACTCTTATGCCTATGGGATATTAAT

AAGGCCGTCCTGACACATTGTATGCCTATATTTACTCGACATAAGGGCAG

CCCTAAGGGGCAAGAAGAGTGGAAGCAATTCTATGACCTATGGCATGGGA

TTGTTGCCTCACCAACTGAAGATATATACAACGAGAGGCTTAAGAAGTGG

AAGAAGCATTACCTTCCTATGCACGTTGAAGAAGTAGGCTATATCTTGGA

GACCTGGCTAGATCCCCATAAGCAGAGATTCGTCAAGGCTTGGGTTCACC

AACATCTTCACTTCGAGCAATTTGTTACCTCTCGGGCCGAGGGCATTCAT

CAGCTCATTAAGAGTCATTTGAAACACTCTCAAGTTGACCTCTTTGAGGC

CTGGAGGATCATCAAGCTTGTTCTATCGAATCAGCTTTCCGAGCTCAAGG

CCAACCAGGCCAAGCAGCAGATCACCACACCTCTCGATATATCTGGGGTG

TTATATGGCAATATCCGCGGCTGGATCTCACACGAAGCCCTGCGGAAGGT

TGATGGTCAACGGGCACGGCTATTAAAGGAGATTCCCGCGTGTACAGGGG

TCTTCGCAAAGACTCTCAGTCTACCTTGCGCTCATAACCTTCAGCCTTTA

CTGGCGCGAGGTCTGCCCCTTCAACTATACCATTTCCATTCACATTGGCA

CCTCCAACGCTCAGGAACCCCCCCAGCTTTTATTGTCGAGCCTCGCAAGC

TATTTGATCGCCTAGCAGCTAATTCGACACTGCCATCGTCAAGCACGCAG

CGTGAGCCTTCTGCCTTCGAAGCCATTGAAAAGGCATCACAGCCAAGGGC

ACTGCCAAAATGCTCGAGATGCCATAACTATGGCCATAAGATGACATCGA

AAGCGTGCCCATTGCGATACGAAGATCTATTGGAGCCCCCCCCCTCCGAT

CACCACGACAACGCACACATTAACACATACAGCTACTCGCTCTGTAACCC

GCTCGCTATCCCCATCATCCCCATCAGGAGTCTCCGTTATATCCGAGATT

GTAGCTTATACAACAACGCATATGACGACACACACAACAACACATATGGT

ATCTCCTCCAACAGCTAAATCGGCAGCTATTACTACACCACGTGTGCCTG

TATTACAACCTGATGACCCCCGTGCTATTTTCCAACGATATAAGGAAGCA

CGAGAAGCTTGGTATGCTACACTGCCTCAAGGGGGCCTCAAGACTAACCA

GCAGTATCGGAGGGCTATGGGGTTGCCTCAACGCTATAGCAAAGCGGAAT

ATAACTGGTGCCTTGACTATAAGCAAATGGGGAGGCGTTGTAGGGCCGGA

ACTAACATGAGAGAGTGGACTAAGGAGGAGATGATGAGTTATCTTGACTG

GGATAAGGCTGAGGACGAGAGCGTTGAGCAGAATGTAAGGATAGAGATGG

CAAAGCAGCCCTTCTCTAGGCGGCGAGGCATGCAAGATATCTGGGATGCC

GCCGAGAGGGATCTTGAAGTACATTATATATCTTCACGATAGtatttgat

gtggaaatagagaactcttaccaaaggaaaaggctctgctttgtctaccg

cacgc

>Hop3

gggcgtcaataggtgcgcacctggatgcgcacgtaagtgtcacgtgttgc

gcacccggtgcgtctgtaagggagttatgtaagcccaacacccttgtagc

tacccctttcccgttgataaagagcctcacttcgccgcgcaacgatctac

tcaaatcatctctcctagaATGAATCCAGTTACAGCTACTCGTCAATTTT

CTGATGACTGCCTGCCTCCTGAACGCGAGTATGGCTCTCGGGAAGCCCTA

CACGCCGCGATCAATGCATGGGCAGCTCCCAGGGGCTATGCGTTTGTCAC

TGGGAAGTCGAAGAAAACAGAGAGCGGCAGACGAATTGTTTTCTTCAGCT

GTGACCGTGGAGGAGCACCTCCAAAGGCCTCGGGCGTACGGCAACGGTCA

ACTACAACACGGCGTACAGGGTGTCAATTCTCAGTCCTTGCGAAGGAGGC

CTTGGATAAGACAACGTGGCGCCTTACACACCGTCCTGGCAGCGAGTTTG

CCCATCACAACCACGAGCCAAGCACAAGCATGTCTGCACATCCAGTCCAT

CGTCAACTATCCAATGCAGATAGGTCAACTATTAACAACCTTGCAAACGC

TGGTGTAGCACCGAAAGAGATCAGGTCCTACCTACGTCAGAACACAGAGT

CTCATGCCACCCAGCAAGACATCTACAACTGCATTGCACAAGGCAAACGA

GACCAGAAGAAGGGCCAGAGCACAATCCAAGCTCTTGCTAATGAGCTTGA

GGCTGAGGGTTTCTGGAGTCGAATACGCTTCGACGAGGATGGTCGGGTTA

CAGCTGTGTTGTTTGCCCACCCAGAGTCGCTAACATACCTTAAGTCATAC

CCGGATATACTTATATTGGACTGCACATATAAGACAAACAAGTATAGGAT

GCCTCTTCTCGATATCGTCGGTGTTGATGCCTGTCAACGATCATTCTGCA

TCGCCTTCGCCTTCCTCAGCGGCGAGGAGGAGAAGGACTACATCTGGGCG

TTAGATCGGCTACGTTCAATGTACGAAGCCTGTAGCGCAAGGCTGCCATC

TGTGATCCTTACAGACCGCTGTCTGGCCTGCATGAATGCGGTATCTCATT

GTTTCCCGGCTGCAGTATCGCTTCTATGCCTTTGGCATGCCAACAAGGCA

GTCCTGCGTTACTGCCAGCCAAGCTTTATGCGTCATAACGATGCGGCTCA

AAAGCCTCAAGGCCACCAAGAGTGGAAGGACTTCTACGGAAGATGGCATG

AGCTCGTGGCATCGGCAAATGAGGAGACATTTGAAGACAGGCTTCAGCAG

CTCAAAGAGCGCTATGCTTCAGCTCACGCCCGGGAGGTCGCCTACATCAT

CGAAACATGGCTTGACCTCTACAAAACAAAGCTCGTGAAAGCTTGGGTTG

ATCGGTATCTTCACTTTGAGAATGTGGTTACATCTCGAGGCGAGGGTATT

CATCAGCTCATCAAGGTGTACCTTGATACTTCCCAGCTAGATCTCTTTGA

GGCCTGGAGGGCTATCAAGCTGGCGATACTTAACCAAGTAGCTGAGCTCC

GAGCAAACCAGGCGAAGCAGCAAATCCGAACACCCATAGAACTCTCAGGG

AGTCTATACAGCATCATACGAGGCTGGGTATCTCACGAGGCATTGCGGAA

GGTTGAGGCGCAGAGAAAGCGGCTCCAACAGGACAGACTCCCTGCCTGCA

CAGGAGTCTTCTCAGCAACGCTTGGGCTCCCTTGCGCTCACACAATTGAG

CCTCTTCTACAACAAGCCCAGCCGCTTCAACTACACCACTTCCATACGCA

TTGGCATCTTCAACGAGAAGGAAACCCTCAATTGCTCATAGAGCCCCGCC

GTCAGTTCGATCAGGTGCCAGCTACGTCAACATTGCCGAAGACTAGCACT

CAGCGCGAGCCATGTGCATTTGAGATCGTCGAACAAGCATCACAGACGAG

AGCCAACCCCAAATGTTCAAGATGTCATACACAGGGCCACAGGATGAGCT

CAAAGGCATGCCCATTGCGATATGCGCATCTTGTATCTCCCTCAGCTTCT

ACAGCGCCCCCAAATACGCTGCCGCCATCGACTACACCAAGTTCGGCTCA

ACAGCCAGTTCCCGATCCATCCTTATGCTCAATTTTGCCACAGCAGCAGG

TAGCGCCGCCAGCGGCACTATGTGATCAAGAGATGGTGCCGTCCCCGCCA

GATATAATAGCACTGCCATCCTCATCAGCGGATGTACCTCCACCACCAGA

TATGCCGCCATCGGTACCAGCGTCTGGGCCAGACATACGCTATGATGACC

CTCGTGCTATATATGATCGATATATTGCAGCACGGATGGCATGGTATAAC

AAGCTGCCTCGAGGTAGCTTAAAGACTAACCAGGAATATCGAAAGGCAAT

GGGCCTCCCTCAACGCTACGATAAGACAAGTTACAGCTGGTGTTTGGATT

ATAAGCAGATGGGTAAGCGATGCACATCAACGATACCTGGACGGGAGTGG

ACTAAGGAGGAGATGATGGCGTACTTGGATTGGACCAGGGTAGAAGATGA

ACGTGTGGAGAGGCAAGTCGCCCAGGAGATGGGCGACAACCCTCTGGCGA

ACAGGCGAACAGGCATGAAGGAGATTTGGAAGAGGGTTGAGCAGGACATG

ATCGACCAACAAGCTCTGTACTCGAACGATAAACTGGCTGAAGACTGTAT

CATAGTTACTGCTTAGattattattaacgcctacattacttatatcccaa

ttatggcaccgtgagaaaaaatctgtatagcagagcagttcgtcatctag

agattagaagcaatacaataaaatacaataggctggctggcatacaaggg

tgttgggcttacataactcccttacagacgcaccgggtgcgcaacacgtg

acacttacgtgcgcatccaggtgcgcacctattggcgccc

>Hop4

gcgatcaaccagcggcaggcagattatcacatacgcatgtgatcgatggt

gtcggccaccaagcgcctcaagggagcgccagcgcaagactactacgcga

ggaaccaactgccggttctctatcatcgcgaaggaatccctagataagac

gacttggtctttaaggcatcgaccggatccacaattttcttcacacaacc

atgaaccaagttggcacaaatcggcgcacccagtccatcggcagctctcg

gatgtggacaggtcaacaatcagccgccttacgaacgctggtgtagcgcc

aaaagatatcaggacttatattcgccagaactcgaataccattgcaaccc

agcaagatatctacaaccgtatcgcagatagcaaacgagagctctgtgag

ggtcagagcactatacatgcttttgctaaccagctagacaaggaagggtt

ctggaaccggATGCAGCTTGACTCAGATAACCGAATCACGGCGGTGTTGT

TCGCCCATCCGGAGTCATTGGCATACTTGAAGGCTTACCCAGATCTACTT

TTCTTGGAGTGCACATACAAAACGAACAAATATGGAATGCCGCTGCTTGA

TATGATTGGTGTTGATGCCTGTCAGCGATCCTTCTGTATCGCCTTCGCAT

TCCTTAATGGCGAGGCTGAGCAGGACTTCATATGGGCCTTGGACCGGCTC

AGGTCCCTGTATGAACTCTGCAACACAAGGTTTCCATCTGTTATTCTGAC

TGACCGTGATAAGGCCTGTATGAACGCTGTAGAGAGCTGCTTTCCGTCTT

CGATCTCTCTGCTATGCCTGTGGCATGCGAATAAGGCGGTCCTCCGCTAT

TGCCAGCCAACATTCGTACACCATAAGCAAGGGTCTGAGGCCTATCAGCA

AGGTCTGAGCGATTGGAATGACTTTTTCAACCACTGGCATTCGATTATGA

GGTCATCAGATGAACAGGCATTTGACCAGCGCGTACAAGAGCTTGAAAAG

CGCTATCTGCCTCAATATCTCGAAGAGGTTGGCTACATCAAGTCCAACTG

GCTCGATCCGTACAAGCAAAAGCTCGTTAAGGCCTGGGTCGACCAGCACC

CTCACTTTGGTAATGTGGTCACCTCACGGGTTGAGGGTATCCATGGGCTC

CTTAAGAGCCACCTCAAGAAATCTACGCTAGATCTCTTCGAGGCGTGGAG

AGCTATGAAACATGCGCTTCTTAATCAATTATCTGAGCTGCGATCCAACC

AAGTCAGGCAGCAAATGCGAATACCAATTGAGCTCTCTGGATCACTATAT

AGTGCCATACGTGGCTGGATATCTCACGAGGCTTTACGCAAAGTTGAAGA

ACAGCGGAAGCGGCTTATGAAAGAAGACCTGCCTAGTTGTACCGGTACAT

TCTCTCGATCCCACGGCCTCCCTTGCGCCCATATTCTCAAGGCTCTACAA

GAGCAAGGCCAAACCCTTCGCTTAGAGCATTTTCACCCGCATTGGCATTT

AAGTCGTCATGGTGTTCATCAGGTGCTATTGGAGCCTCGACACCGATCTG

ATCGAATAGCTGCCGATTCTACCACAAGTAAATCGCAATCGAGCACTCAG

AGAGAGCCAAGTGCATTCGAGGCAGTCCAGGCAGCAGCGCAGCCAAAAGC

ACGACCTACATGCAGTAGATGCCACAAATTAGGACACAAGATGACGTCAA

AGGCATGCCCTCAACGGTACGAGGGGATTTTGTATTTGTCATCATCGTCA

GCACAGGTAGAAGCAATGCCAGTGGCACTCTCGCCAGCATTATCCGAGGA

AGCACGACCAGCAGCAGCAGCATCTGCATCTGTATCATCTTTGCAGGAAG

GGGAACCACTGGAAGACCCGTCATCAGGATTATCCGGACACGCTGGGTCT

GGCAGGGATGATACTAGCTGCATTGTCGTTAGTATAGAGACAGCAGCATC

ATCGGCAGTGTCATCAGCAAAACCATCGACACAGGGAGGGACGTCGCCAG

TAGCATCATCTTCAACAGCATCAGTGGCGCCGACACTAAGATATGATGAC

CCACAAGCTATCTATCGGAGATATGTTGCTGCTCGAGATGCTTGGTATAA

GGCGCAGCCTCGTGGTAGCGTCAAAACTAACCAGCAGTATCGGAGGGCGT

TAGGTCTACCTTTGCGGTATGACAAAGCAAGCTACCAGTGGTGTCTTGAC

TGGAAGCAGATGGGCAAACTCTGTACGACACCGACCGGATCTAGAGATTG

GAGTAAGGAAGAGATGATGGCGTACCTGGATTGGAGTAAAGCAGAAGATG

ATCGGGTGGAGGCTCAGGTAGCGGCAGAGATGGAAAGCAATCCTTTTTCT

AGCAGAAGAGGCATGCGCGACATCTGGGAGGCGGCTGAGAATGATGGAAA

AGAGCAGCAGGAATTGTATTCAGCTTGAtaattaggaattcattgcagtt

ctgaatctcggaattctgaaacccgccagcctcaaaactcgaaatgcagc

tgtggagttgtgccaaattgggggctgacataatccgcacgcctacgcgt

gcagaagtggagaggcgcctaggcgtgtccctatggggccccc

>Hop5

ggcaattactacaccggcgtgacagaatggctacactggcgtcactctta

cacaggcgtcgtttttatggcctctgattggcccatccctttttgttagt

gcatttagattctgccaacaccgctcgttgtacctctagcttagctcccc

accgcgacgatcgacgcgccccatgaagctcttcaccagctagctactac

attaattacctctttgccgcgtgtagtgttagagaagtggcatgcaagat

ggtgttacacaggcagtcacctcaagggacgatgtctcacgatgatacca

gaattgtctgaacagcattacagatctgttgcaggattggcaggtctcgt

atcttggcctttcctcttcagatattgagacgaggagggagaacgaagaa

caagaaggacactacacatctacagatcagtcagagacatgatcatgagc

ctaaatcaatcattcacggttctccgaactcgatatacacttctccatct

tctgattatccactccatcattatatgagcacgcgtgtctgacatgtaga

gcctacttagaatggtttttctcgaaagatacggtatttgttaaggtcaa

agtgctgggggtgtcctgtgttggacccctgctgagaccaaccctcgaac

acaggctgtatcagatatcgccaacggcctcatctttaaaccatctgcat

cacaaagcaggactttcgtttctgctgaaatgagacccttctttccttct

ttcaccgtcaaatcaagtacccttttaatcatcttctcaccgtgcatgcg

acacaccctatcgcgatccagaccgtcATGTCCCAGCCCGAGTATTCATC

TAGCTCCCTAAGCAACCAACGACAACGAAGCTATCAATTCCGTCCTCGAA

AACGACAGCGGCAGGACCAATCTGATAGTTCAAGACCACCTTCGTCTAAT

AGCACAGGGACGAGCATTGGCCGACACTATGGCGTCGGAGACGATGAGGC

ACTCTATAATCTCTTCGCCTCGTATCCAGAGACGTTCACGACCGCTCCAC

GGCCCCATGGGTCGCGCGCGCTGAACGAGCCACCATGGGGGCTACGATTC

GATCAAGAGATTCACCTTTCGAACGATTTCCCAACCCAAGGAGGACGACC

GCAGCGAGTAAGAATACCTATACCAGGCCCGGAAATCGACAAACTGAGTT

CCTGCTCTCCACTGCAACCAGCACAAGAGCAATCGTCTCCAACAAGGCCC

CAGTTTCAGTCCGCCCTCAGGCTAGTGCGCGAGATTATTGGGACAGACAG

GCGACTGACCTTCGAGCTCGTCCGGTCATACGAACAAGTCCGCCAATGGC

GTGAGAGATGGGGGTACTCGCCGCTCAGTTCAGATGCTCTCGAATCACCC

CCGCCCTACTCGCCTCCCCAAGACAGGCACTCTCCAGCTATAACCTCGCC

ACCCTTGCCCGACCACTCGTCATCGCCTGTCCCTGACGCGATTCCGAACC

CATCTATCCCAGGCGACCCAGCGCCGTCAGCCGAAGTCCTGTTCGAGTGG

GTGAATACTTTCGCAAAGGCTAATGGCTTCGGTATTGTTCGACGAAATGC

CCATTCATATAAGGGCCGGAGGATCCGGTACACTTTCCAATGCGATCGAT

TCGGAGAACCAGCGCCTAGTCAAGGCGCAGGTCTTCGACGACGAAAGTCT

CGAAAGTGTGGATGTAAATGGATGGTTGTTGCCGAAGCGTTAGAGGAGGG

GAAGTGACTCTTACGACAACACTCGAACCTCGAACACAGCCAGCATAACC

ACAGCCGCAGCATCGGGCCATCTGCTCATCCCTCCCATCGGAGACTCACC

GAGCTCTATCAGGACAACGGTCGAATCGACAAGCCGCCGGGTCGGAATTC

GGGCCCGTGACGTACGTGCTATCGTCCAGGAGCAGCATCCCGAGTCATCC

TTTACACGAAAAGACATCTATAACGCCAGGTCTCGTATCAACCGAGACAA

GCTGGACGGCCACACTCCTACTGCAGCCCTGATCAAGCTGCTTGACGAGA

TGAAGATTCCTTATCTCGTTAAGTGGGGGGGCGATGAGCCAAACCGTCTT

GTTGGCCTTGTCTGGGCCTCTCCTTACTGCCTCCAGATGTGGAAGCGGTT

CCCCGAGGTCATCAGCTTCGACAACACATATAACACCAACCGCTTCAAGC

TTCCCTTATTCCAAGCAACGGGGCAGACATGCCTTGGCTCTGTCTATAAC

GCTGCATTCGGTCTGATCGATAACGAGAGGAGAGAAGGGTTCCAATTTGT

CTCCGGGAGCATTCGGCAGCTAGCAGAGCAGCACTCCATCCGGCAACCGG

ATGTGATCGTCACCGATTTCGACGACCAGATGAAAGCCGCATTGAACGAC

CAGTTCCCTGATGTACAGCAGCAGCTGTGTATCCACCATATCAATTCGAA

CGTCCTGCTCAAGTCAAAGCAGAAGTGGGTTAGGGCCCGCAGCACCAGCA

AAAGTAGCAGTCCTGATACCAGCGACAGAGAGGCAACTACCCCGCAGCCA

CAGGCTCAACTAAGCCCCCAAGGCCGAGGGTTGGTTCATACGCCAATAAC

CGAGGCTATCCCTCATAACTATCGAGGAGTTCTCATCATGTGGAAGCTCG

TCTTATTTGCTGAGACCGAGGAGGCCCACGAGGAGGCATGGGCAAACCTC

TGTAAGGAGTTTGACGACCAGCGAGCTATCTTGCGGTATCTCCATGGAAC

CTACATGCCCGTCAGGTCCCAATGGGCCCGCTGTTTCATCCGGAAGTATC

GCAATTTCGGCGTCCGCGTCACATCTGGGACAGAAGCAAGCAATAACAAC

ATCAAGAGCTATCTTCTGAATGGTATGAGCAATATCTATCGCCTGTTTGA

GGCTATGCAGGACATGATGAAGGACCAAGAGCGAGATTTCAACGATGCCT

GCGCAGCGGATGAGGTACTTACCGCACGAGAGTATATGGGGTCCAGCTCG

GAGTATCTTGGCGAACTCTGGATAGCGCTCTCTTCGAAGGGTCTCGGACT

GATCACGAAACAATACCAGCTGGCAAAGAAAGCAATGCCGACAGGAAAGA

ATCCGTTTCCTGACCCTCTCGGGGATTGCGGCGAGGAATGCTCGGTTTCG

ACCGAGCTTGGTATCCCCTGTTGCCACAATATATACTCAAAGCTGGTGAG

TGGAATGCCTTTCACTAGGTGGGAGGTCCATCCGCGGTGGCGACTACGAG

AGCCATCCTCTCAAGATCCATACCGACGAATCCTCGACCCTAGGATCGCA

ACTGCTCTCCGTGGCAGACCTAAGAACACCACACAGGCCGTTCCAGCGCG

GCTAGCAATCGAGGCAAGCTGTCAAACTGATAGCCGGCCGGCCAGAAAAC

CAGCAAGCCAGGCAACAGGGCGCAGACGAGGTCGACCACCAGGACGTCTT

AATAGGCCAACGCTAGCCAGGCTTCCACTGGAATCGAGCCAACAAGTTAG

CAGCCAGGCTAGTAGCCAGGCCAGCACCTCATCCCAGCGCCAAACGCGCA

GCCGGTCAGCTGTTCTTGGAGGTGGACGAACTATAGGAGTGCGTGCCAGC

GGACGGAAGACGCAACCTAGCATTCGTAGGCGAAGGAGTCAGTGGGAGCT

GCTTGACAGCGACGAGGAGGTACTTCCTTCGATATCAGTTAGAGGATAGc

gagatagatagctgccctccagggcatacttgaattggtctggttctgca

gtgaaaattagaccttgttgtgatgttcgctttagaactggttgacccga

ttggtttatcaaaagcgaggacttgttaagggagctgccttcaccgcctt

tgcgcggatgtacctgtgggtcgatggaaaaaaggagcgaatcagaggcc

ataaaaacgacgcctgtgcaagagtgacgccggtgtagccattctgtcac

gccggtgtagtaattgcc

>Hop6

ccgtccttctaaaattggacgggtcccgtcccagttgccatatctggtat

agctcactccgctatcggtctcttttcgcgccgtttcgaatgtcgcgttt

tattgcactcgttgctaatcatgtcgagactatgggcttacgacgactgc

ttggctatgaacgtcaaccaatcttaaagtccaACACCGTTTCCTGACGA

TTGTCTCCCTCCCGAAGGCGAGTATGAGTCACGGGAGGCGTTATTCGAGG

CAATCAACGCCTGGGCGGCGACCAGGGGCTACGCGTTCATAACTGGAAGG

TCAACTAAGGAGAAAACCGGCAGGAGGACAATCACGTACATGTGTGATCG

TCGCCGTAACCATCCGATCGTTTCGAGAGAACGTCAACGCAAGACGACAA

CTCGAACAACAGGCTGCGAGTTCTCTGTACTTGCGAAGGAAAATAATGAT

AGAAGTACCTGGACTCTGCGGCACCGTTCTGACAGCCGATTCTCACTACA

TAACCATGAACCAAGCCAAGACACCACCGCCCACCCAGTACTTCGAACAC

TGTCTAAGGAGCACCTGTCTCAACTGACTGGCCTTGCCAACGCTGGGATA

GCCCCTAAAGAAATTAGAACGTACATGCGGCAGAACACCGACACGATAGC

AACACAACAGGACATCTACAATCGTATTGCAGATGCTAGACAAGAGGTGT

GTCAAGGGCAAAGCAGTATTAACGCACTTGCTGATCAGCTGTTTAGAGAA

GGATTTTGGAGTCAATTTCAAACCAGTCCAGACGGTCGAGTCAAAGCTGT

TCTATTTGCGCATCTCGAGTCAGTGGCATATCTACAGGCATATCCGGATG

TCTTAATCTTAGACTGTACCTATAAAACCAACAAATACGGCATGCCACTT

TTGGACTTGATCGGTGTCGACTCCTGTCAACGTTCGTTCTGTGTCGCGTT

TGCTTTCCTGAGTGGTGAGTGTGAAGAAGACTATTTTTGGGCGTTAGATC

AATTCCAGTCGCTGTGCGAAAACCGCAGGATAAGGCATCCCTCAGTCATC

TTGACTGATCGCTGCCTAGCATGTATGAATGCTGTCGATACTTGCTTCCC

ATCATCAAGGCCGCTGCTATGTCTCTGGCATGCTAACAAGGCAGTTCTTC

GCTACTGCCAGCCGAGATTTACGCGCCAGACTCAAGGGGAAAACCCGGGA

ATCGAGGCTTGGAATGAATTTTACAGCCACTGGCATTCGATCATCAAGTC

ACCAAATGAACAAACTTTCCACGAACGTGTGTCCGAGTTTGAGAAGAAAT

ACATCGCGGATTATATCGAAGTAGTCAAATATATCAAGACAAACTGGCTT

GATCAATACAAAGAGAAGCTGGTGAAGGCGTGGGTCAATCAACATCCCCA

CTTTGACAACGTCGTTACTTCAAGGGTGGAAGACATACACTGGCTGCTTA

AGAGCCACCTCAAAGTATCTACATTGGACCTGTTCGAAGCTTGGCGATCG

ATCAAGCATGCACTACTCAATCAACTGGCGGAGTTGAAGTCGAACCAAGC

CAAGCAGAAGATCAGGACACCTATCGAACTCTCCAGTATTCTCGTATTCT

CTATGGTGCAGTACGTGGTTGGGTGTCTCATGAAGCTTTGCGAAAGGTTC

AGCAGCAACGAAAGCTTCTATTTCATCAAGGCTCTACTCCTTTGTCGGCC

TGTACTGGTTCTTTCTCACGATCACAAGGGCTGCCCTGCGCTCACAAGCT

GGAGGGCTTATTAGCTCAAGGGCAGACTATACAACTCGAAGATTTTCATC

CGCATTGGCACTTGACTAGGGAAGATAATCAAAGGCCCCTCTTAGAACCC

CGCCAGCGAGTCGATCCAGTGACTACGAATTCGTCTTTGCCGTTATCTAG

TGTGAGAAGAGAACTTAGCGGTTTTGAAGTCATTGAGAAGGCGAGGCCGA

TACGAAACCCTCCTTTATGCAGCAAATATCATATACTAGGTCACGCAAGG

AACTCAAAGGAGTGTCCACTACGATACTCGGAACTAAGAGCATTGCCCGT

TATATCTTCAGACCAGCCGCAGGTGTGTTCGAAAACCTTTGGGTGGATAC

CCTTTACGACCCCTGAGGAACCAAGGCCACCAGTGATGAAAACGCTCGGC

GAAATACAGTGGCAACCAATCACAACTTTGGAAACTTTCGAATTTGAGAA

TGAATCAACTTGTCAAGATCTGCCATCCCAGTTGACCATCGAAACAGAGC

GGGTGGATCGACACATAACGACTCCAGAACCTCGACACCCATCACCTACG

CCATCGACGTGTAGACCGCAGGTGTCACCACAGTTAAATTCGAGAGATCT

AGCTCCAAACCCAACTACACATCTGAGGCATGACGATCCACGAGCCATCC

ATCAGAGATACGTCAAGTCTAGAGAGGATTGGTATAAGGCTCAACCCCCA

GGAGCCTGGCTTGGTAATCGCCAGTATCGAAAAGCAATGGGCTTACCTTT

GGGATACCCAAAAGCAAGCTACAGCTGGTGTCTTGATTATAAGCAGATGG

GGAGGTGCTGCAAAACCCCGACGGGTCCCAGAGAATGGACTAAAGAAGAG

ATGATGGCTTACTTAGACTGGGATAAAGCAGAAGCTGATCGAATTGAAGC

ACAAGTAGCTGAGGAGACCGAGAACGGACGACTGTTTACAAGCAGAAGAG

GTATGGGCGAGCTGTGGAAGATAGCTCAGCGGGATATCGATGAACAAGAA

GCCTTGTATGCTGCCACAGAACAGGAGGAGAGTTGTATTGTAGTTCAGTC

CTAActttagtagagtttataatgagcttatagctcggataaatcaagac

tatacaagttaaagtcagcgatttgctttgagtaaaattagcggttgatg

gacttcaagca

>NhORF4-like

caactcaaccaactcaatggaggactttattagtaagctgccaactcaat

attaccttgaaattgatggatctcgttgatttcgttgatataaagtgggt

ctactagccaccgttagtgactgtaactgcggattggtataaatcctaac

agcgacccgtaactaaggctatgtgtggcttgtacgtatggcagatcacc

taatttccctggccgccacctccttttagcctctgttctgccctcaattc

tgcttccaacttccacatcgatttcaacagctacaccgattgcattgtgt

aactagttcaggATGAGTATTTTGTCAGATTCTGACGCCATCACTGCTGT

TGACGATGATGGAAATTTTAGGTCGTCTCCAGCGCCTTCTACAGCTTTCA

CGCCATTCTCGCCAGCGCGACGTTCTAAGTCCACCTCGCCAGATTCCACT

ACCAAAGAGCGACAATATGAAGAATCTCTTTGGCGCCATTTCCCTGGGTT

CACGTGGTCTCAAAGGGTTAGAGATACTAATTCCTGGGCCTGGGAGTATG

GGTACGATATCCAGAAAGGCAACGACAGGAAGTGGGTTTGCAAAATTTGC

ATCCGCAAGAACACTCTTAAGCCAAAGACCTTCACATCAACGGGCATCCA

AAATACCCTAAACCACCTCTATGACGATCACAGGATTTGTGCGCCTGAAG

GGAAGACGAAATCAGCCTCGCAGCTAAGAGCAGAGGGCCGgaagtgtgag

ggaacgatcatgtgactttcatattttatgaatagtctcactttatgaat

ttgtgtaaatagcttaagtcagagctttccttaggaatctctttctctcc

ttgtaaacaatcattttacccaagtcgcacccgacctcgttcgtgccctt

gtaccgtcacagGAAGGCCAAGGGACAGTCATCCATCGCCGAGTTGATGA

AGCTTGATACCAATAAGCCTAGAGAACAAGCAATTGCGAATGGCTTCATC

AAGAACTTCGACAAGAAGCATTTCCAACGACTTCTAATGGAATGGATCGT

GGAGGCCAACCTCTCGTTCGAAACGGCAGAACACGACAAGCTCAGGAAGA

TTTTCGCATATCTGAACCCTTGCGTGAAGCTCTGTGATGCCAATCTCTCC

GCAACCTCTATTCGCAGGAAAATCGTGGTATCATACGAGCAGCACAATAC

GAAGGTTATGGAGGTCCTCCAGAGCTATCCCGGCCTAATTCACGTCTCTT

TTGATGGCTGGAGGTCCGGAAATCGGCACGCGTTATATGGGATTATGTGC

TTCTTCCAAGACGAGAAGAACAATCCCTGTAAAATCGTCCTTGGAGTACC

CGAGGTCTCTACTCGCCATTCTGGCACCAATATTGCAGCAGAAGTCCTCG

AGATTATCGATTCATATGGTATAAAGAATAAGATCGGGTATTTCACGCTT

GACAACGCCGAAAACAACGATTCAGCAATGGCTGTAATCGGAGGAGAGTT

GGGCTTCGATGGACGCAAGCGCAGAGGTCGATGCTTTGGCCACATACTCA

ACCTCTCTGCCAAAGCATTGCTTTTTGGCTCCAATCCAGAGGCATTTGAG

AATCAGCTTTCCGGCGCAGCAGCGTTATCTGAAACTGAACACGATCTTTG

GAGGAGGAGAGGGCCAGTCGGGAAGCTCCACAACCTCGTTGTTGACATCG

ACAGATCTGATGTGCTGAGTTACCTTCTTCGAGGTGTTCAGCAGGCTGAT

ATGGACCAATCTATCGATCCGAGAGTTCGAGCGCGGAAGCCTCTGAACAT

CGTCGTCGATAACGATACCAGATGGCTGTCCCAGCTCTACATGATCCGCC

GCGCGATCAAACTCCGTCCTTACCTCGATGTGATGATTCTTAAACACAAA

CAGGCTTGGGAGCAGGACAATAGGTCGAAAAGGACGGGCCTGATGAGGAG

GTCGGCTGTTCAACCGCGAATTTGCCTATCCGAAAATCAGCTTTCAAACA

AGGATTGGGACGTCCTCGAACACCTCGCCACGATTCTTGGATTCTACGAG

GTCACCGTCAAAACCCTTGAGGGAGACGGTATTCAACGCAAACGGAAGAG

AGGGTGGGTCGGCTCGTACGGAAATATCTGGGATGTCATCCAAGGCTTCG

AATTCCTAATGGCAAAACTGGAGGAGTACAAAGCGTTCGCGGCGGATTAT

CCTGATCCCGAACACTTCAGGATCAACATCAACCTAGGGTGGCAGAAGCT

CAACAAATACTACACAATTCTCGACGAGACACCAATATATTACGTGGCGT

TGGCTCTCCACCCTGCGTATCGATGGGGTTGGTTCGAAGAACACTGGGGA

AAACATCCGGATTGGATCGCAACTGCGAAAGAGATGGTTCAGGAGGTATG

GGAGACCGAATATCGCAACCTCGAGGTGGTATTGAGCCCAGGAAATGAGC

CCGTTGCTAAACGGCGAAGGAAGTACCAAAATGCCTTCGAGGAATACTGC

GAACAGTCACGATACGAGCCATCAAGCCGGTTATCGACGCCTACGACCAC

GCCAGCGAGGATGAGGTCTTCTGCGACTCCTGAGTCCCCGAGGGACGAGT

ACGAGGCGTGGCAACAAAGCTGGGTTCCAACAGACACCGATATTCGGGAC

CCCATCGCATATTGGCAGGATAACCGAGCTCAATATCCCCGCCTCTCTCG

AATGGCTCTTGATTTCCTCACGATCCAGCCGATGTCAGCCGAGTGCGAGA

GGCTCTTTTCTGCCGCAGGTCGATTGGTGACTCCCCTGCGCAGCCGCCTT

GAAGTCGACATAATCGGGATGTGTCTAGTGCTGCGTTCGTGGATACAAGC

CAAGATAATCGACGACCTGGATCCTCTGTTCGTTCCCACGCAAATCAAGG

TCGAGAGCAGCGCCAGAAGCGAGGCGGGCGCAAGAGAGTCAATGGCGTCA

TTTCTCGGCCGAGCGAGAGAGCTGGCAGAAGAGGAGAAAGGGTGGGAATA

Gttggtggggcatgttgcgaatatctcgtcaattaggccaactgggacgg

gacaagagcacgaacgaggtcgagtgcgacttgggtaaaatgactgttta

cgaggagagaaagagattcctaatgagaactctgacttaagctatttaca

caatattcataaagtgagaatattcataaagtatgaaagtcacatgatcg

ctcccttacaccaactcaacgtcaatccattcaacgaaggcgttcagttg

atcaactcaacgagaggtcggcgattactaagccaattcactcaatatag

gtcgtccgttgagttggttgagttg

>Frodo

cagtgttgcgaataatatggcaataaagtagtatattgcaatacatacca

tataatatgccttttctggtattgtgataatatgatatgaagtctacgat

attgtgacaatataatatgaggatgtccatattggcaatatattatctat

attgggctgtcaattcttgggtgaattagccccgtgaaagctgcagaact

cggtcttaccctatataagaagcagggatagctggcattttattggccat

cgttcacttccacgcacttctttgttgacaattagacctaacacgcgatc

acacatgaaagaaacgcgtgagggagtgcagaccatgttgggttgggcca

tcttcgcctctgctccccccaattttttttcttttttttttcttttgttt

ttttgaaaatcactcgtcgaactcaactttacagatctaactgccATGCC

TCAACAACGCCTCTCTTTTGCGCAATGGTCACAACGAAGAGGGTCGTCAA

TTTCTGGCGACCTTCTGGAGTTTTCCAGCCAGCAATCGGATGATCTGGAC

GTAAGCCAAAAGTCCAAGTGCCTGTCATCTCGACCTCGCACCGTCTCCAC

AACCTCCTCTACCAAGCCCAGAACATCATGGGTCTTCTCTCATATGCCAG

ACGAGGAGGTAGAGACGAGATACTATAATCAGCGGACCGGAAAGGAAGAG

TGGCGCTGTAAACATTGCGACAAAACCTATGCCTCTTCAGGGGGTACTGC

AGCTCCTGCAAAACATCTCATGGACCCTCCTCCTGACGGTCACGGCCTGC

CAAAAGGAGCCCCGCGAACAGCGAAAGTTACAACTATACGAACTATTATC

GAACAAGCTCGTGTTGCGGCAGAAGAAAATCCCCGAAAACGCCGTCGACT

CAACGATCAATCCGGAGACTCGATCGAACCCGACCAGCTCGAGGCGTTAT

ACGTGAGGTTTATCACGGCCTGCTCTCTCCCATTTCGGCTCGTGGAATGC

CCAGAGTTCCGAGCACTCTTGGCCTACATCAATAACGATATCGAGACTTG

GCTTCCGGATACGCATGATACCGTCAAGACATGGATTATGCGCCAATATG

AATGCCAGAAAGAGAGGGTCAAGCAGCGCATACAGTCGGCAAAGTCAAGA

ATTCACATCAGCTGCGACCTTTGGACTTCTCCCAATTCTTTGGCCATTTT

AGGCGTAGTCGCACACTATGTCACGGAAGACGGTCAGCTGGAACACCATG

TTTTGGCGTTGAAAGACATCGACAGTGAGCACGACGGTTCTCATCTCGCT

GTGGCCATTTTGAAGGTGGTTGACGAATGGGGCTTTGCTTCCAAGCTTGG

ATATTTCGTGATGGACAACGCTGGCAATAACGACACCATGATGAGATCCC

TTTCGCTTGgtcagtacaactcactatttcccctgactggcggccctaac

accccacagGCCTCCTACGCCGATATGACATCCAATACGATCCCAAAGTG

CACCGACTCCGCTGCCAAGGCCATATCATCAACCTCGCCGCCAAAGCCTT

CCTCTTCGTCACCGATAATGAGAAGCTCGAACTCGACGATCCTGGTGTGC

ATAATGTGACACTAAAACATATTGAAGCGTGGCGGGAAAAGGGGCCTCTT

GGCAAGATTCACAACTTCGTCGTTTTCATCCAACGAAGTGTCCAGCGCAG

CCAAAAGTTTCTTACTATTAGCCATAACCGTAAGCTCGCACGGGACAACG

ACACAAGATGGAGTTCGTGGTATAACATGCTGCGAGTGGCCCTGAACCTC

AGAGATGCGATTGACGGCTATTTCAACAAATGGATGGAATTAGATTGCGC

TGGAGACGAGCTCTCTTCAGAGGACTGGATCATCTTGGAGAAAATCAAGT

CCTTCTTAGAGAAGCTGAAGATGACGACCAAGGCTCTCGAGTCATCGTTT

GCAACACTCGATAACGTCCTCTTGGCTATGGATTTCGTTTTGGCACAATT

CGAAGCAGGGAAGGAGGCATATATTGACGATCCGATAATGGCGCCCATGT

ATAACTCTGGCTGGGCCAAGTTGGACAAGTACTACCGGCTCACTGATGAA

TCGCCTGCCTACGTTGCCGCTATCGTGCTCCACCCTTCGCATAAATGGCA

CTACATACAGGAGAACTGGAAAAAGGAGTGGATTGAGTCGTCAAAGAAGT

TGATGGAGACGCTGTGGAATGACTATAAACCCGTGGAATCACCTCTTCCT

CTCTGCGAGGTCCCATCGACGACCACGAACGAGTTCTTGAACTGGAGGAA

CAAGCACCTGCAGCCGTCGCTCGTCACGGATGAATATGAACGATATTGCA

ACTCTGAACGGGTTTACGGGTTCACAAGCGCCCTTGCATGGTGGCTAGAG

GAGACACAGCAGAAAAACTACCCAAACTTGAGTAAAATGGCGGTGGACAT

ACTGTCAATTCCCGCAATGTCTGCTGAGCCCGAACGACTCTTCTCCGGGG

CCAAGATAACCATTACAGATCGTCGAAATCGGCTTGGTAGTGATGTAGTT

GAGGCTCTGGAGTACCTGAAGTCATGGTTTGGGATACAAGAGCTTCAAGG

TGATGTACTTTAGttgccgcagtagaagaacccagatcacatgcaatata

tgggatgtattgtcatactggcaatatattgggttcttggcaatacaata

caatataggccaatgtcatatggcctaatacaatatgcaccacttggcaa

tacaataatattgcgaggcttccatattgccaatatacgtattgtttata

ttattcgcaacactg

>Gimli

CAGCGCTCGCAATAGTCCAGACCATTTGGACTGTTAGTCCAGACTGtaga

tcagggcagggaatacggacgcccggtgtggacgctaagaacgctaggtt

gttggtatggtgttacaaaaccagggcacccatgattgttgctgtaccaa

ccattttattttggatatttccgatcaatttattggtgcgatccgatcaa

cacggctcgtttcaagggacgcatcatcctagccgtcaatccacgaacgc

ccgtcgtggattgattgttgattttggattgCAGTCTGGACTAATAGTCC

AAATGGTCTGGACTATTGCGAGCGCTG

>Sam

CAGACCTGCGTATTTGGAtatgatatatcgatatcaactgtgctatctgg

atatatcgatattgatatatatcgatatcgatataactctctgcctctta

aattgatatcgagattgatatccacctaaataccgctctctgattagctg

aaaagatcttatcaatcgattagatgatgaaaaggcttctgtcgcatatc

accactaggttcgtgcgactggatatgaaacacggggcgtaccttggggt

cgcgtatccggtttcacatctggcctgtcgcataatcctaacatgggctc

aaatagctgttcccgaacacatggctgttcgatttcttttgttctttaga

taactctcatcaattgacgggtgccatcaatgatccctgtggagccaatt

gtctgacaacttctctcaatttcaacatcagcaccaccatctcagtactc

gttcaaacggccctaggtattctagcataattagcttatccaggtgattt

atcgaccATGGCATCATCTCAGACCACCGCCGCCTCTACGCCCTCGATTT

CGGACCAGCCCATTACTGGGACGTCTCTGGACTTTTCTCTCTTCTTCAAA

GCAACGTATCCTGATGAGGACACCCAGAACACCACCGGCGGGTCACGTAA

GCGCAAATCCAGGGGTATGGTGATGTATCGATGCCTCCATTGCCCTGCCG

ATAAGCCCTGGGCGAATCGGAAGCGGGACAACGCATGGCATCACGCCCGG

CGGTGCCATGCCGATATTATCTCTTCCCTCGATCGCACGCTCATTGGGGG

CAGTTCTGATGTCCTAGATGATGAAAGGGAAGCCACACGCCCTCGGATTG

ATGCTTTCTTTCCCTCTCGTTACTCGGATGTCTCTCTCCGCCGTATGTTC

AACCGCGATCGGTATTTAGATGCAATAATCAGCCTAATCACACGCCGCCG

GCTTGCCTTCTCAGCAATTAACTGGGATGAGATGCAAGAGATTATGCTAG

CAGCTAATCCTGCTATAGAGGACCTTTTAGTGACCAACCGAAGTGCTCTA

ATGCGACTAATTGATGCCACATATGAGTTATATTCTTCTCAGCTCATGGC

AACTCTTGAAGCTTCCATCTCAAAGATCCATATCCTCTCTGATCTCTGGA

CATCGCCCCATCGCCATGGGATACTCGCCATCTCTGCGCGATGGGTTGAT

CAGGATTATCGGCCACAAAGGGCACTGCTAGCGATGCCGGAATGTCGCTA

TAGCCATAGTGGTGAAACCCAGGCATCTTTAATTATCGATACACTGGCAA

AGTACGGCATTGCTTCTAAAGTGGGCTACCATGTAGGGGATAATGCCACT

TCAAATGATACCTGCTTGTCTTATCTCTCACGGCGGTTACGAGACGACTA

TGGGgtacgatagcccttcccttatctctggattaggattaatcggttgc

tagttgacgtttgatccctctaagCGCCGTATCCGCTGCGTCGCCCACAT

TATTAACCTATCCCTCCAAGCATTTCTTCTCGCGTCTTCGAAAGAGGCAC

TAATCGCCGCTCTTGATGCCATCGATGATACCTCAAACGATCAGTTGTTT

GCACAGTTCTACGACACCTTGCACGACGCCGGACAGACAACAAGAACAGA

TGAAGCCCATCAGAGAAGAAGAGCTTCGCGAAGAGGCAATCGCATACTTG

AGAACTTCACTGGATGGCAGCATATCGTGCCCCTACGGAAAGTACACAAC

ATCGCGGTCTGGATACGTAAATCTACGCTTCATACGGCTATATGGGACGA

TGAAATAAAGCTCCGACTAGGTATAGATAATGCCACACGCTGGAATTCAT

GGTATCGATTGTTGGATAATTTTCTGCGTTATCAATCCCGTGTAAAACAG

TTCCTAATTGACCATGATAGAGAGCTTCAAGACGATATCCTACTAGCTTC

AGAGTGGGAGTTTATTGAACGGACGCATCGTTTCCTACAGCCGTTTGCCT

CAGCTACGTTGTTGGCCGAAGGGGCGCGATCTACACTCTCCCAATCACTC

TCGATAATGGATGTTTTATTGCGACAGTACGAGAAGTATAAGgtcggtgg

cgatgttttttcttttatctaattgatcggctaactaattgtcttattta

gGAGCTTTATAGTTCAAAGGAAAACCATGATCCCCACATGGTACACTGCA

TCGATATGGGCTGGTTTGTACTTAATAAGTACTACACCTTATCAGACCAG

ACACCTGTATATGCCGCCGCACTCCTTCTCGATCCCTCGAAACGAAAAAA

ATACATTGAGCGGAATTGGGAAGAATCCTGGCACGCGCCTGCTATTGCTG

CTGCCCAGCAAATTTGGCTAGATGAATATAATGCCGCGCCAATCCCAGAA

TCCTTGCGGGTGCCTCTAGATGTGTCCTCTTCCTCTGGGAGGCAGCACAA

CGAGCTGGATGAGTTGTTGAGCGATATTGCTGTTACTGGTCCTATCTTAG

ACGACGCCGATGACTTCGAAACCTTTATTGATGCACCACCGACCCGAATA

ACTGGCTCTCCTCTCCAGTGGTGGCTACATCGAGATCAAAGGAAAGCATA

TCCGCGGTTATCCCGCATGGCAATTGATATCCTCTCAATCCCTCCTGAAT

CATCTGATGTTGAGTCTCACTTCTCATCGGCGCGGCGTACGCTATCATGG

GATAGAGAGAGCATGACCTGCGAGAACCTGGCGAAGGTTGAGTGTGTGGG

CAACTGGATGCGCGAGGGTATCATTGTACCCAAGTCTCACGGTGGCAGGG

GCGTTATTTCCAGCGTTGCTGTTGAATTTAGCGTTGAAACAGAGGCAGAG

GACTTTCTAGATTAGggtactttcttgttctttcttctagcctacaagaa

ttatatgtggtttatctggatatcaaccttgctaaagcggctatgtggct

gtatggatatctacctggatatatcgatatccagataatttctcccactt

atctggatatcgatataagggccagaatttcGGTGGATATCGATATACGC

AGGTCTG

>YahAT1

ctagggttccgtttttgaccgtcaaaaacggtatttgacggtcaaattcc

taaagggggtttttggcggtaatttacgtcaaaaaccctacaattttagg

cttagtgcctacccgtattaggactagtatatgcacggaataggtttagt

acatcgtaaaattaccgatgggacgcacaaatcatcaccaattctcatct

tcggtgttgaaaagcaatggacatgagactgtcaccATGAGTAACAAAGG

CGGTCGCCCTAAAACCCATGGTGTTCATGAACACTTTGCAGCAGCCCCAA

TTCCTCCAGGAGGGGGTAAATTCAGTCAACTTCGCGTTCGGTGCAAATAC

TGTTCTAAAACCATGGCCAAGCACGTTCAACGTCAACAGAATCACCTTGC

CAAAGAGTGTCTTGGTTTCCAGCAAGCTGCAGGCCATGTTGATCGCCAGC

CTCGAATCACTACGGCTTTGTCCATAATGGATCGAAACTCTAAAGATCAA

CTCGATCGAGCCGCAGCCCTGGCCATCTTTAAGTCTGGCAAGCCATACAC

CACCTTCGAAGACTCTGGCATGGTTGAGTTCATCCAACAGCTCAATCCAG

CGTACAAGCCCCCTAGTGGTGATCGAATTGCCACCATACTTCCTGAAGTT

TACCAAGATTACCGAGTCCAAGTCAAGGAGATCCTCGACGATGCCGATCA

TCTCAATGTCATCTTTGACGCTTCTGACGACATAAGCAGCAACCGTATTG

TCAACATTTCGATTTCAATTCCCAACTCGGTTACATTCTATTGGAAGACT

GTTAATACACGGAACGAGGAACATACGGCCTTGAATACCATTCGCCTAAT

CCAGCCACTATTGGAAGAGGTATTCACGAAGGAAGGAGAGGACTTCCAGG

ATTTCTCTCGTATGAACGCTATCTGCACAGATACATGCTCGACAATGCGG

AAACTTCACGCTGAGATGAAGAATATGCCTGAGTTCAGGCATTGTTTCTT

CCTTTTGTGTGACTCCCATGGTCTTCAACTTCTAGTGAAAGACATTGTGG

AATCGGAGAGATGGGCTTCAGTCCTTAAGAAAGTTACGAGAATCATCACG

TTCTTTAAAAAGGCCAAGCTCCAACTCGCACGACTTCGTGCTTATCAGCT

TGAGTGTTACGGTAAACAGAAGGCTTTCATTACACCAGCCATCACTCGTT

GGGGAACACAACTCGGAGCTGTTATGTCTTCACTGGCGAACAAAGATGCT

CTTCGAGCATATGCACGTGATGCTATGGTCCTCAATTCCCTAAAAGCTAA

GGCTATCAAAGGGGAAGAGGAGGAAGAGGAAGATCAACAAGTCCTAAGGG

CAATACTCTCGTCCATCAACAACTTTGAGTTTTGGCATGAGGTAGAGATG

TTGGCTAAGATCCTTCGGCCTATCGAAAACGCACAACGTTGCTCGGAGCG

AGATCGCTCATGCGTTGGAGAGGTTATTCCCCGTTGGTTGACTATCCAGA

GCAGATGGGATGCCCTTGAAGAGGCTAACCAAGATCCTCTTATCAACTAC

TCGGAGCTGAGGCAATGGCGAGTCCAAAGGATGAAAGGTCAAACGACAGA

CATCCACCACTTCGCATTTGCCTTGGACCCACGGACAACAATAGGGGAGG

GCCTTGGGCTTGACACTATGGAGAGAGCACATCGCTTCCTAGAAGAAAAC

ACTAGTAACGAAGAGTACCAACAGCTCTTTGGGGAGTTTTGTCGATTCCG

TGCTCGCGAAGGTGCTCTCTTTGGCCCAACCTCGCGGATCTATCGAGAAC

AGCCGAAGACTCCTCATACGCCTTCTCACTATTACATCCTAAGCGCGTGG

GGATACTTTGGGTCTATGGGAGTCAAGTTGGCTACATTGGCAAGGAGAGT

TCTAGGATCCTTGGCAAACTCTGTTCCTTCAGAGCGTTCCTTCAGTGCTA

CGAACTATCTTCATTGTAAGCTTCGGAATCGGCTTAGTCCGCTTAGCACT

GACAAGGTCACTTTCATCTATATGAACAGTCGGGTGCTCAAGAGGCTCAA

GACGGTCAAAGATAATCCGGCAGAACCGATGGTCGTCAAAAACTGGGAGA

CAGTTGATGTTGCTCGGTTAGTAAAGATAGAAAACGCATTCCAAGACTCT

ATTGTTGTCGATGGCTGGCGTTGGGAAGAGACCGATTTGGAAAATAGTGG

AGATGATATGGACATCGGCCAATGAgaggtttttgacatgtaaaatacca

agaagtttcagacgtcaaaaactttggtcagataggtaaattaccattcg

gtaaattacctcgacggaaccctag

>YahAT2

cagccaatcaaagtcatccatcaagccctccattagtgagctaccccaat

accaccgtcaggctggtgggggagaaagtagcttcttaacttttacgtcc

cgtcctagcaacatcaagtcgcgtcagtgctggtttgtttacctttcaac

gcgtaccattcctcccatgagtcgacgcctaacccgATGGAATCCGACGA

CATCATGCTTCCTTCGCCTCCACCCTCTGCCTTTTCTTTTCTTCGACCAC

CGAGGAATTTGGCGAGTCAATTCGAGCTGACGACGCAGTCGGCATTGCCA

GACCACCCTACTGTTGCAGATCTTCCGCGAGCAGTGTGGAGGAACAAACG

ATATGTTTTGGAAGAGTCCTTGTCCCGGAAGGGCTCGAAGGGCCGGAAGA

GCTGGATCAAGCGCCATGGATTTTTTCTTGTTGAGATCGACACTAACGAT

AGTCCTTTGAGTCCTTACTGGGCCTGTCGTTTGTGTGACGCGAAGGGTCA

GCCTGAGTTCTTCGCCGCTGCTGCTACGAGTTCGGCAGCGGACCATCTCC

GCAAgtatgtgaatgtgaatgtgacgtcgcgagcttgagcattattgacc

aacgccagGTCTCACAGGATTTTCGAGAGCAGTCAAGCAGCTGATCCAGA

TCTGTCAACCGATGAATCTGAACGGCCTAAGCGACGACGCTTGCAGTACA

GCGCCGTGCCGCGTGCTAGAGTCAAGATGATCCGAGAACTGAGCCTGGGA

CTTCTGATCAACACCAATGTTCCTTTTTCCTTCTTCAGCGATACATTCTT

TCAGCAGCTCGCTTGGCAGCTTGACCCTCACCTAGCTGACCAGATACCAT

GGAGCCGGCAATCGATGGGCCGTTTGCTGGATGACACGTACAAGTCTAAG

AAAGATGAGATCAAGCAGGAGCTCTCAGATGCCCTTACTAAAATCCATCT

GGGGTTCGACCTGTGGACATCGCCTAATCGGCATGCTGTTATGGCAGTCA

CAGCTCATTTTCTTGACCGCCAGGGCAAGCACCAGTCGCGCCTATTGGCA

CTCCGTCGCCAGCTCGGGTGTCATAGCGGAGAGAATCTCGCGGTGACACT

TGGGCAAGTGGTGCGTGAGTGGAAGATAGAGGATCGAGTTGGAACAGTGA

TCTCGGACAACGCATCTTCGAACGATAGCTGTCTCGTGAACTTCTACGGA

GATCTCGACGCAAAGATGAGTCTGGCGGACGTTCGGGCCAGACGTATGCG

CTGCTATGGGCATATCCTGAACCTGGTTGCTCGCGCCTTTCTCTACGGTG

AAGATTTCGAGTCTTTCGAGGCCGAATCGCAGGTATTTGACCTTCTCGGC

CGGCGCGAGGATGACCTACGACACTGGAGAAAGAAAGGGCCGGTGGGAAA

GCTCCACAATGTTGTCAAATTCATCAGGTCTTCCCCTCAGAGGTGTGAGC

TCTTCAAAAGGATCTCACGCGAGAACGACGAAGCACAAGAATACCTCTTG

GCGAGTGAGTCAACGGCGGAGCTAGAGGTCGTCATGAATAACGATACGAG

ATGGAACTCCACTTACCTCATGATCTCTCGAGCGCTCGTCAAGCAGGGAG

ACATCAGAGCGTTCTTGGTCCACCCAGAAGTGGAGAAATGGCTACCGGAG

GCCGACATGCTGAAGGGAGATGACTGGAGACTGTTGGCGGAAATCAAACT

TATTCTTGAGCCATTCTATCTACAGACTATGAGAACGCAGGGTTGGGGCA

GCGAGGGAGGTAACGGACGGCTCTGGGAAGTAATGGCGGGTATGGAGTAC

TTGCTGGAACACCTAGAGGATCGGAAGCTGTTCCATCATGCCGTTCCAGG

CGAGGCAGGGGGACAGGACACCAACTCGCAGGCCGAGCTGGCTCGAGAGC

GACCAGACCGTAACCGGCAGCTTCCTGCTCGATTTAGGGATTGCGAGACG

GATATCCATCCGCGAAAGTCCAGGCAAGGCCCCTTGTCAGATCGTGGCTC

GAGACAATGCGATGATGGATCAGGCAAGCCATCAGGATCATTAGGAATCG

ATGACATGGGAAAGGATCACCGACACTATTTAAGACTGTCCATCATGACT

GCCTGGCAGAAACTAAACGAGTACTACACCAAGCTTGGAGACTCACCGTT

GTTCGCTGCATCCATCATCCTCCACCCATCACTCGGTATGAACTATCTGG

AGGTGAACTGGGCGTCGGAAGAGCAGCTTGTGTGGGTGAGGGATGCCAAA

ATTGGACTATCTGACTACTTGGACCGTTGGTACCACTGCAACCGGCCGGT

GGATGAGCAGCAGAAGATGATCATGGACACATCGACATCCTTAAGCGTAC

CAAGGACGACAACTGAAGTTAGCGTGTTCAAGCAATGGATTAAAAGCAGG

ACGGTGAAGACCACGGTGATGGGGAGTGAGCTTGAGCGGTATCTGCGGCT

AGAGCCGCAAGAGACTGAGGATCCAATCGAGTGGTGGATGGCTCACCAGG

GACAATTCCCGATGATCAGCCAATTGGCTCTCGATATACTCGCGATACCG

GCGATGGCGACTGATTGCGAGAGGTCGTTCAGCCTAGCCAAGTTAACGCT

GACGACGCAGAGGCTTTCGATGACGACGGAAACGCTGGAGAAGTTACAAT

GCCTTAAGAACTGGGTTAGACATGGTGCGGTGAAGCTAGGGGCGACAATA

GGTGGGGGGGACGAGGGCCAATGGGAGATTGGAGATGTTAGCATGGAAGC

ATAGagagtcagatttgaaatatttgaaatatttgaataaatatttcaaa

caacaaatccagtcttgtgagttatcaaatatctcaaatatgacctcgga

tttgatttgtttgatttgagggcagcgctg

>YahAT3

caggccttctatccatacgcgtatggattaattaatccatatggaataat

ccatacgcgttaatccatattccatacggggatgtcgtattccataagta

tggcaatacggcggaaaaattttgatccctgtagtaatcccgttcagggg

cgattttccaactctccactctttctttaattccaacaccactgtgtagg

gacgctgcgtATGTCCTCGCTGATGACGTCCACTTCCACTGACCCTGCGG

GGATTCCAGTGTCCACAAGCGAATTGTGCTCTCCCTCAATCTTCAGCACG

AACCCTCGATTCTCAAGCGGCGATGTCCAAGAGCGCATGGCTCGAAAGCG

TAAAACGACCGCAAATACTTGGGCTCACGCCCGTGAGCCCCTCAATTCGG

AGCCATCTCGTTGCGGTCGCAAAAATGAGAAGATTTACTACTGCATGCAT

TGTGTAAGTCCGACTTACTCCACGACCGTTTCAACGACCTTTCGGAATCA

TCTGCTCAAGATACACGGCATTGAGCTGGAAGCCCATGAACATCCGATTA

AAAAACGGCGTGACCGTCTGATTCAGGATGCTTTCGCCAAGGCCGGCGAT

ATGCATGCTGCAAAGCAACTGGCAAAACGAGAAGAAACTCTGAGACATGC

CATCAATCACAAAGCAGCGTTGGAGGCACTTATTCAGCTCGTGACTGTCC

GTAATCTCTCTTACAACTGCAGTTCTTGGCCAGAGCTTCATGCGCTCATT

TCTGCAGTCAATCCTGCGGCAGATGACCTCATCAGCCTTTCTCACGGCTC

CATACAGAAGCTTGTCTCCAATTCCTTTCGTGTACACAAAGACATGTTGC

GAAGAAAATTGCAGTCTACACTGgtgataaccatggatcaaccgacaaat

tatgccgcttactcaccaattaactccagGAGAAGGGCGTCGACTGGGAA

GCCAAGACCCGTAGAATCCGGTGCCACGGCCATATAGTGAACCTGGCAGT

TCAGGCTTTCCTTTTCATAGACTCAAAAGAGGCTGCTCGAGCGGCTTTGG

GGCATATCGAAGACACCGATGAATCGGCTTTTGGCACTGATTTTTCTGAA

AGGATCAAACCACAAAGGGCGCAAGGATGGCGGCGGCTTGGGCCTCTTGG

AAAGGTGCACAACATATCGATTCACATGCGCGAAAACGACTATCGATGGA

ATGAATTCAAGAAGCGCGCTGGAAGATCGCTAGGGCTCGACAACGATACA

CGGTGGAACTCATGGTTTCTCCTGCTGGACACCACTCTCAATCTTCAGAG

CTATGTTGAGTGGTACCAGAAGAAGTATTGCCAGGATTTGCGAGATGACT

ACCTCACGCCTGATGAGTGGAGTGCTCTAGGGGAGACACGCGCATTCCTT

CAACCGTTCTGGAAGATCACTCAGCTTACAGATGAGAGCCCTGCGTACGG

TGCGGCAATAATACTTCATCCGTCACGACGTGTAGCCCATATAAAGAAGA

ACTGGCCAAAGTCCTGGCATAAACCAGTTCTTGACGGCGTGCGAAAGCAT

TGGAAAGACTACTACCACGAGCTCCCGCTCCCCACCACGACACCACAGCT

GAGAGACGAAATACGGCGCCTTGATGAGTATGACCTACTCGCTCGGGAGC

TTGACGTTGTTAGTCCATCTATGAGTGAGCTGGACGAGTACGATGCTTTC

ACGACGCAACCGCCCATTGTCATTGACTGCTCTCCACTTTCTTGGTGGCT

CAGAGAAGAACAACAACAGACATACCCTCGTCTCTCTCGAATGGCTGTTG

ATATCCTCTCGATACCAGCCATGTCTGCCGAGCCGGAGCGCGTCTTCTCC

GGGGCGAGACGCACAATTTCATGGGACCGGTGCCAATTAGGTAGTCGTAC

AATTGAAAGAGGCGAATGCATGAAGAGTTGGATCAAAAGCGGGATAACAC

AAGGTTATGCGGTAGATTTACTTGAAGCGGAAGGTATTGAGGACAACGTA

GAGGGGTTAGATGATTCGGCTGGCTGGCAGAGCTCCAACCCAGATTCGGT

TTAGttgataaggttattgtaaatgacgtcatttcttagtttccatatgt

atggattaagttccatatgggttacgttccatattccatattccataagg

cgccgtatggagtatggagtatgaatagaaagcctg

>YahAT4

ccaagggtgtgctcaactcaacgtatttcaatatgttgaatggattgacg

atcaacgtcaactcaacgtcaatcaatatcgaatacggattacataaaca

tcaatccattttgagttatcattgattgaatggattgaaggtggtggatt

tagggtagctggtaataggactcgtgattcctgaagagattttggtatct

tgaataggtttagtctgtgcgatccacccttcccgttatactaactttaa

tatggtttagctcatcacttggggttttttccctcttggatcagtgccca

cgcgctccATGTTCGCGTTCTTCCGCGACCGTGATCCAGCTGGACGCTCT

GAACCAGAAGGAATGGAGTCTACAGCGCCCTCCACGAACGACGTTGACCT

GTTATCTTCCGCAACACTTGCAGTGGCTTCTTTAGCGACGCCAGCTCCCA

GCCTGCAAGAGGAAAGACCACATGAAGAAGTAGAACATCAGTACCTCTGG

CGTTGCTTCCCGGATTACGTTTGGTCCCAACGCGTCCGAGATACGCCATC

GTGGGTATGGGGCTTCGGGTATGATGTTGAGGATTCTTCTGGTGCCCGGC

GGTGGGTCTGCAGGCGCTGCATCCAGAATAAGAATCCTAAACCCAGAAGC

TTCGCGGAGAAGGGAATCCAGAATGCGAACGCCCATTTATTCAAGGGCCA

TGGAATACGCGCTCCCCCTGATAAGACGAAATCAGCCGCTGAGAAAAAAG

CCGAGAAATTAAAGGCCAAGGATCAGAGGTCCATCGCCGAAGTGATGAAG

CTCGATACGCGACTCCCGCGCGAACAAGATATTGCGAACAGTCTGGCCAA

GGGTTTCGACCGCAAGCATTTCCAGCGCCTCCTACTCGAATGGATTATCG

AGGAAAACCACGCATTTAGCGTCTGTGAGCAAGGAAGACTACGCCAGATA

TTCGAATACCTCAACCCACTTGTCAAGATCACGGATGCCAATATTACCAG

GACAACCATCCGCCGCAAAGTTCTCTCGGCCTACGAAATGCACAAGGATA

AGGTCGTCGCAGCGCTGAAGCAGTCATGCGGCCTGATTCACGTCTCCTTT

GACGGATGGAAGTCAGGCAACAGGCACAGCTTGTACGGGATTGCTTGTTT

CTTTCGGGATGAGAATAGCCAGCCACGCAAGCTGGCGCTTGGTGTACCGG

AATTGAGAACTCGCCATTTCGGACACAACATTGCGGCCGAAATACTGGAC

GTCCTTGACGCCTATAGTATCCAGGATAAGATTGGGTACTTCACACTGGA

CAACGCGGAAAGCAACGACAAGGCCATGGAGGTAATTGGTGGTGAGCTCG

GGTTCGTCGGGTCGAGGAGGCGTGGGCGCTGCTTCGGCCATACGCTAAAT

CTCTCGGCCAAAGCACTGCTGTTTGGCCACAACGTCGAGGCTTTCGAAGA

GCAGTTGTCAGGGGAAGCTGCTCTGTCTGAGGCGGAGCACACACTTTGGC

GGCGCAAAGGACCTGTCGGAAAGCTGCACAACCTGGTGGTTGACGTCCGA

AGGTCGGACCAGCTTACTTATTTGCTGCGGAGTATACAACGCTCCGAATT

CGATCTATCTTCGGACCCCAGGATTAGAGCGAGGCAGCCGGTCGATTTGA

TCATTGACAACGATACCCGGTGGCTTTCTCAACTATACATGATTAGACGC

GCCATCATTCTTCGACCATTCATCGAACAACTCGTCTTGAAGCATCGTCA

ACAGTGGGAGCAGGACAACAGATCAAAGAGGACAGGGAATCTCAGAAAGT

CTGCAAGGGAGCCCCGGATTTGCTTGGAGGAAAACCAGCTTACGGTCAAT

GACTGGGTAGTCCTGGAACACCTGGCTAAGCTTCTTGGATTCTACGAGGA

CGCCGTGAAGACTCTGGAAGGCGATGGCCAACAACGCAAGCGGAAAGGAG

GGTGGGTTGGCTCGTACGGGAATGTATGGGAGGTTATCCAGGGATTCGAG

TTCTTGTTGGAAGTACTCGAGGACTATAAGCAGCTTGCTTCTGAGATACC

CGACGCGGAGCACTTCCGAATCAATATTAATCTGGGTTGGGAAAAACTCA

ACAAGTACTACAGCAGGCTGGATGAGACACCAATCTATTACACGGCCTTA

GCGCTGCACCCGGCTTTCCGGTGGGGGTATTTCGAGAATGAGTGGAAGGA

TAACACGAAATGGGTGATGAAGGCGAAGCAGATGGTCCGAGAAGTGTGGG

AATCGGACTATCGTCACCTGCAGGTGGTCCGGAGTCCCGTGGACGACGAA

CCAGTTGCGAAGCGGCAGCGAAAGTACTACAACCCATTTCAAGCGTACTG

TGAGCGCACGCGGCCGGTTCTCGGATACGGTTTGGTGAAAGAGGAAGCGA

CTTTGTCTGACGACATCAATGAAGATATTAACGAGCTCGAATTATGGCAG

TCGTCATGGGAGGATGGAGATAACGATGTTAGAGATCCAATATCATACTG

GCATGAGCGCAAACGTCGGTATCCTCGTCTTTCACGAATGGCGCTGGACT

TCCTTACGATCCAGCCGATGTCAGCAGAGTGTGAGAGGATGTTTGCGGCG

GCAGGGCGGATGGTGACGCCACTACGAAATCGGCTGGACGCGGACATTAT

AGGAATGTGTCAAGTGCTGCGATCGTGGTTACGGGCTGGGGTGATTGACG

ACCTGGACTTGTCACTACTTCCTACTGAGAATGGTAGTGGTGATGGGACG

GAAGAAGGATCCTGGGTAGAGGTGGGATGTGAAGAAAAGGGGATGGGAGA

GTGGGAGTGAactagctcggcttggagatcaacgacttcaaccagtgtcc

attcaactcaatttgacggataattgatcaacgccactagagagagtggt

tgaccaggtcaactcaccgccaccgagccattcagttgattgaattgagt

ggtgcacacccttg

>YahAT5

tacaaatacggggggctgtacaaaaactccgttggctgagcagctcaaca

cactctgccattctctcctgattgaaggtatcgccaaaaagATGGCGCCC

ACCCAAGCTAACGCTGCCTTCGTTTGGCGACAGTTTATCGACCTCGGCCG

CTCTAACTCCAAGAAAAGCAAGCGATACAGATGCCGTCACTGCCAAAAAG

ATTTTGCAGCGACCTCTGTTGGGAGACCGAAAGAGCATCTCGCTGCGTGT

GAGAAATGGCAAGCCAAGCAGCGACAAGAGCGTCAAACTCAAGATAACGC

TGGCTATCTTCCCTCCTACTCAGAAGCCATACAAAAGAAGATAACCGAGG

TCGGAATTCAGCATATCTCGCAGGACGAGAGTCACAGTTATGCCTATGAT

GCCGCCGCTGCTGTAATCGCCGGTGGTCGGCCCTTCAGCCTCTTCGAGAG

CCGCCGTTGGCGTTATTTCTTCACTCGCATTAAGCCTGGCTGGAAGCCTC

CTAGCCGCGCCGCCATCACGAGAATTCTTCCCGACTTCTATCAGGAACTC

TATGACGAGGTTTTCAAACGCATTACCAGCTCAGAATGGCTCAACATCAT

ATTTGACGCTTCAGATAATGTCTCGGGTCACAGAATCGTTAATATCTCGG

TACAGCTACCAGACGGCCCAGCCTTCTACTGGAAAACGTTTGACACGGGA

GACGAACAGCACACAGCGGAGAATTGGGTGAAGCTGATATGGGGAGAAAT

GCAGCAACTGTGCGGCGGTGATCTCTCCAGAATCAATTCTATCTGTACAG

ATACCGAAAACACGATGCGCTCTGTACACGGCTTGCTAGGGAGATTCCCA

GAGCTCTCACATATCAACTTCTCTCTATGCGATTCTCACGGCCTTCAGCT

TCTAATTAAAGATATCCTTCTTCTTCCATTCTTTGAGGATCTCTGTAATA

ACGTCAGCACCCTTCTCAAGTTCTTCTCACGGTCTAAATTACAATTGCAA

AGATTGAGAATGTGCCAACGCACAAGGTGGAACGGAGTAACTCGCGCGCT

GATCAGAAGGTACCGgtaaatcgttagctgtgactgagaaatcgatatac

taactggctcgttccgaatagTATAATTACACGCTGGGGTTCTCAGTACA

ATTCTTTCTTCTCCCTCCTCCGTTCTCGAGACCCTGCCAGAGACTGGTCT

ATTCGAAAAGACGTACGGGATGAGCTGCGATCTCAAGATTGCCCCGTTCT

CCTGCCCGAAGCGGTTCGAATCATCAAAGACAACAGCTTCTGGCTAAAAC

TGGAGGCTGCGATCGCTGTGCTAAAACCCGTGAACGAATTTCAACACGCT

TCCGAGGCTGATGGGGCGGGAATCGCACACGTTGTAAATCGCTGGCTGCA

AATCAAAAGCAAATGGTCTGAGATGGGAGAGGCTGATCAGTTTCCTGATA

TTCCGTGGGACGACATTGACGCTATATTCAAAGCCCGGCTCGATAAGCAG

ACGTACGATATACATTGGATCGCCGATGCCCTCCGGCCTGATACAACAGG

TCCAAATTCGAAACTGCCGCCTAGCGTCTTTGCGCGCGTACAAGAGTATA

TGCAGAAACAGCTGAAAAACGACGACGAATATCACCGTGCCCTTTCCGAA

TTCACACACTTCCGAATGCGTACAGGCGGCCCAGACGGCTTGTTTAATAA

GCACAGCGCTGTATACGATGATGGCTTCAAGCCAGCAATGGCGTGGCAGT

GCCTCCTCAACCAGGGTTCGATTCTGGCTAGAGTAGCCGTGAAGGTAATG

AACACGCTTGCGAACTCTGTGCCCTCAGAACGAAGTTTCTCTGCTATTAA

TTTCATACACACGAAAGCTCGAAATCGCCTTACGCCCATGCACGCTGACA

TGCAGGCTTTCATTTTTATGAACGACCGCGTATTAGATCGCCTTAAGGAT

CAGAAGTACGCCCATAAAAAGCGCTGGGCGGATCTTGAGGAGAAGGACTG

GCTAGAACTCGAAGATTCATATCTCGAGTTATTTGTGAATGCGCAGGGCA

AGAAGGTCTGGATGGATGGTGTGATGGCCTCCACCAGTGGAGATTTTGAG

TGGGAGGGTGTATTACAGTCGACGGGTGATATATTGGGGGTTGGCACTGA

GGTGGAATCAGAGGTTTGAggccaaaatggcagaagacgaggaccgtgag

aagcctactaagttaccagtattacctaaaaaggaagggggacatttgta

tggtacatatttgtacgtacaataaggttgtacgtacgtacagcggctaa

aatacagtacgtacgtacgtacgtgtacaagaaattgcatcacta

>YahAT6

caaggttccgatcaactacaccgaaacccaccggttgatcgattggtgta

ttactcatacacttacatcaatcaacctgaaccctcgacgtgatcaacta

ccctacatcgacgaccctggttgatgtaggtgtagtggatttttgcagcg

gtggatttttagtgggacagggtatccagttttggacatctggatccacc

ctgttaggaggtactgtcttttgtgagggcagggccacacagactttagt

atttcctaacttattttcgggcccccagtagttatagaggatatcctggg

gtaatttcccgtacaggtccaaattttcatcgatctgtttgacgcattca

cacctcccgtaATGACCGATCCTTTAGAAGACAAAGTCGTTGAGACCTCT

CCACCAATTCTTTCACCATCAACGTTACTAGCTGATGCCCAACAGCTACA

GGAAGAACGTCTGTTTCGTCATTTCCGCGGCTGGCTCTTCTCCGAGCGCG

CAAAGGACACCTCCTCCTGGACTTGGGACTACGGGTATGATATCCAGCGG

GCTCATGAGCGAAGATGGGTATGCAAACTCTGCATTCACCAACGAGTCCC

CCAGCCGAAGAACTTCACGCATACTGGTCTTCAAAATGCCAGCAAACATC

TGTTCAAAGAACATCATATTCGGGCGCCAGAGGGCAAGACGAAGTCTTCG

ACGCAGTTGAAAGCGGAAAGCGTCGAGAAGCAGCACCGCTCAATAGCTGA

TACCTTGACGCTCGATCCGGAAAAGCCGCGGGAGCAGGCCATGGCGAACT

CCTTCGTCAAGAACTTTGACAGGGACCATTTTCAACGGATGGTGATGCAA

TGGATCGTTAAGAGTAATCTTTCCTTTCTCACGGTCGAAGACGAGGATCT

CCGAGCCATATTCGATTATCTTAGTCCATCAGTGTCAATACGTGGTGGCC

ACTTATCTGCAGATACCCTCAGGACCAGGATTATCACTGAGTATCAACGA

CACCGCCACACCGTTATCGACGTCCTTCGACAATCTCCCGGCCTGATTCA

CATCTCTTTCGATGGCTGGACGTCGGGAAACCGGCATACCTTGTACGGAA

TCGCTTGCTTCTTCAGAGATGAGCACAACAAGCCCCGTAAGATTGTGCTG

GGTGTGCCCGAGGTTTCCGTACGCCACAGTGGATCGAATATTGCAGCTCA

AGTCCTTGATATCTTGACAGCCTACCAGATTACCGAGAAAATAGGATATT

TCACGCTTGACAACGCAGAGAATAATGATACAGCGATGGGGGCCATTGGT

CGTGAACTAGGCTTCACTGGTGCGTCTCGCCGAGGCCGCTGTTTCGGTCA

CACGGTCAACTTGTCAGCAAAGGCGTTGCTTTTTGGCAAGAACGCAAGCG

CTTTCGAAGAGCAACTCTCCGGCGCGTCGGCCCTGCCGGAAGCAGACTGG

GAATCATGGAGGAGCAAAGGCCCCGTTGGGAAGCTTCATAACCTTGTCTA

TGACATCTTTCGGTCCAACAGATTGATGTACTTGCTGCGAGATCTTCAAC

AAGATGCAATCAGCAAAGCCAGTTCGTTAAGGGAACGATCGAGGAAGCCA

CTTACTGTGGTGCGAGACAATAATACCCGCTGGCTTTCCCAACTGTACAT

GATCCGTAGGGCACTCAAAATTCGGCCATATCTTATCTTACTGGTGGTAA

AGTACAAACAGGAGTGGGAAGATGAGAATAGGTCGAAGAGGACGGGTCAG

GTCAGGAAGTCCGCAAAACTGCCACGTATCTGCGAGGAGGAGAACAAGCT

AACGGACAGCGACTGGGAAGCCCTCCAGTGCCTGGAGGAGATATTGACGC

ATTATGAGAATGTAGTGAGGACTTTGGAGGGTGACGGACAAGTTAGAAAG

AGAAGGCGAGGGTTTCTGGGCTCATATGGTAACATATGGGATGTGATTCT

AGGTTTCGAAGAGCTCCTCTCGAAACTAGAGCAATACAAATTGCTTGCAG

CAGGCTTTCCCGACGCCGAACAGTTCAGGATCGGGATTAATCTCGCCTGG

GACAAGCTTGAAAAGTATTATTCAGCTCTAGATGAAACACCCATCTACTA

TACCGCTCTTGCTCTCCATCCTGCATACAAGTGGGCGTATTTCGAGCACA

CTTGGGACACCCATCCAGAATGGGTCGACAAGGCTAAGGAGATGGTTTAC

CGAGTTTGGAGGACCGAGTACGCGAATTCCGATATCCTCTCTCAAGCAGA

CGAGTCAGACGACCAACGTCCAGCAAAAAGACGCAAGTTCTTCAGCCCAT

TCGAGGCCAACAGTCGTCATATGCCGGCAAGATACGACTCTTCAGACGAT

GAAGCAGTCAAAGGTGATGAATACGAGACATGGCAAGGTAGCCGCGAAGT

GAGTGATAGGAGAGTTCGCGATCCTATTACGTACTGGCATGAAAGGAGGT

TGAAATACCCTCGACTTTCGCGGATGGCGTTGGACTTTTTGACCATTCAG

CCAATGTCTGCAGAGTGCGAGAGGCTCTTTTCAGCTGCCGGAAGAATGGC

GGTGCCGTCGCGAAGCCTTCTGGATGCCCAGATTATCTCCATCTGTCAGG

TATCGCGGTCATGGTATAGGGCTGGGATCGTCAAGGAACTGGATCCGATG

TTGATCTCGTGGCGCGAGGAAGAGCAGATTTATGAGGGTCTTGGGATGAG

TGATGGCGAGTTAGCAGAACAAGCAACGGCATGGCTGAGGGCTCAGGGCG

ATGAGGGAGAGAGTGAGGGGTAGtaggctgggcaagtaccatccatgatc

aagagcgcaagtcacgtgagcgaagtgtacgacaaaccaaccgtcaacta

catccaccaaaggcgctggtgtagcacactacacccaccacaccggacag

ttgcttacataatttacatttacactactctgggtcgaaaagtgtagttg

gtgtagttgatcggaaccttg

>YahAT7

cagtgatcggaatggtctggtatgagttggtattggtatggtaaataccg

ccaagacttggtatggtttgaagaaatctcaataccatggtattaccggt

ataccaagtcataccagtaaggctaaaatgggctaaaaataggctgagtt

ggtggcgctaggcggctgggttatggaccaaactggctagcgcctcaagg

tcaagaattttctcggcttaaaattacaatcctacaacacgtagttgacc

tatttacggtattgaggtatcacatatggaatctccagttccaacctcgc

cgtcctctctggctacgagctcaacgccattatccttcaagactcctaca

cgttcgcctgctcccacactacctgtctcgacgccatcacctgctgccct

tcagtatcccgagcctccagacgaatttgtacaagacaatatcacgtact

tgaatcgcgaaaagaccacaaagaaagcagcccgtctgggctcatcacat

gtatggaagtatggtcttgctacatccgagacagatgtattactgccacg

agtgtgcagccggaaagcacaagcaagagctatttattatcaatggcacg

tctggagtgagaaaccatcttgaacagaagcatcagatcgaccctcagag

tggtatcaagaaacgtagtcggacacggaagtctgtactggagcagcaga

ggagcgcggctgcgactaataccttcttctggaaagacaattgaaaagtt

caaagaactcctggttcgttggatcgtgtattgccacatcgctttcttcc

aatttgaaaaccagtatttccgcgagctattattctttttaaacccggcg

cttctcagtcatctcccaaaagcaacgaaaactatccgaagttgggttAT

GGATGCCTTTAGGTCGAAAAAGCAACGGCTCAGGGAGGACCTCCGGCAGG

CGAGGAGTAGAATCTCCATTTCCTTTGATCTTTGGACTTCTCCAAACCCT

TACGCTGTTCTTGGGGTTATCGCTATGTGGATCGACACTGCCGGCAAGCG

ACAAACTACCGTTTTGGGTATACGACGCGTGTACGGCGAACACACTGGCG

AGAATATTGGGTCAGCGATTCTTGAATTGTTAAGAGAATATGATGTTGGC

GGAGATCAGATCGGATACTTCATGCTGGATAACGCCTCGTCGAATGATAC

CGCTGTTGAGTTCATACTCAAGGAGCTCTGCCCATGGATGACGCCCAAGC

AACGCCGCCATCGCCGGCTTCATTGCTTGGGCCATATTATTAATCTCTGC

TGCCAGGCATTCCTTATGGGCCGGGACTGCGAAAGGTATCTAGCAAAGCT

CGAAAAACACTATCAACGCGGAGACTATGCGAAGGTGGAAGAGCTCTGGA

AACGATTTGGGTGCTTGGGTCGGCTTCACAACCTCGTGCGATACATTAGG

CTTACCCCGCAACGTCGTGAGGAGTTTACTGCAATAATTGTCGGCGGAGA

TCTCGCCGAATTCGATAGGCTCGAACTTATCCAGAACAACTCGACACACT

GGAATTCGTGGTTTCATTCAATTACGCGGGCGTTGAATGTTCGTGAACGT

TTGGAGATCTTTTCAGCTCGCCATGTACCTGGGAAAGGCTCTCATGGGAT

TGCGAACTTCAAACTTGATGGACAGCACTGGTTCGAGCTTGAAAAGAGTG

AGCTTGCTCTGAAGGACTTCTACGCCGCAACTTTGCTCTCTGAGGGGAAA

AAAACATCCCTCGCCGACTGGTTTTCAACTCTGGACTGCCTTCTTCGAGA

AATAAATGAAACAAAAGATCACTACGATACAATCGATACCGAAGACGACA

ATAATTTCACATGGAAGTATCTTCAGGGTTGCGCAGATGCTGCGTGGTCA

AAGTGCGAGGAGTATTACAGCAACCAGCAGCTGAACTGGCAGAATCGATT

CCCTGAAGATACTGACCTTCCACCAGCGTACTATGCAGCTCAGATACTGG

ACCCATATCGAAAATGGGCATGGTTCAGGCAAGAATGGGTACTCCAAGCA

GGAAAAGAAAAACAAGAGTGGTTTGATAACGCACAATCTGCGGTGAAGCA

GCTCTGGGAGACAGAGTATAAGGGACGATACGCCGTTGAGATGCTGCCGC

CACTAGTCAGAAAGGAGAGATATCCTGACCCCGCATTTGACCGCCAGAGG

GAACACAAACGCATTCGAATAGACGCTCCGGTCTCTGCAGCCGATTTGTA

TGAACAATACATTTCCACCGACCGGCTTCATGACGACGAGGCAGGTTGCG

ATGAGGCTATCGCATACTGGCTATCCCGTTATGACTCCCAAAGAGATCTT

GCTCGCTTCGCTCTGGATCTGTTTGCGGTCTCACCCATGTCAGATGAGTG

TGAACGCCTTTTCAGCAGTGCAAAGCTTACCATAGTTGATCGCCGTGGTA

GGCTGAAGGCGGATATTATAGAGGCCTGTGAGTGTCTCCGGGCTTGGTAT

GGAAAGCCCCGGGTTGAGGAGAACAACGATAGTGAGGACAGTGAGAACGA

TGACGATTGGGTCACTAGCTAGtagtataatacactttctctcgcccttc

tatagtttttcgacgtcgtacttaaaagtcttggcatccttaatggtatt

attggtcttaccggtaataccataccggttattatgtaagcatctggtat

ggtttggtttgaccccccataccagaccataccagaccattccgatcact

g

>FoCrypton

catgcttatcgatggtgcccgcaattacgtcgtaattcgctccgaccacg

tgacgacttatcccccgcaaaaagcggcccgtgaaatcgtgtgcgcggcg

cctgataagtgcttttgataagttccccgcaacttatcaggccccagaca

ggaaacaaacaccacgtagaaaggccttgatcgaccgagATGGCCGCAGA

CGACTCGTACACGGCCGCAGACGAGCGTGCCCGTGCCACCCGGAGGGCGG

GCCTTCAGGCGAAGAAGGACGAACAGCCGGCGAACACGGCACGGAGTTAT

GCTGCGAAGCAGCGGGAGTGGAAGgcgcgttcccccggccgccgccctct

cttttcccctttgcccttgtctcttttttttttacgtagagtcccatggg

cggctaacagacgaggacttatagGCCTGGTGCCGTACACCTCGGGCTGC

GGCAGACGGCTCACTTTATAGCTGGCCCGACGGCGAGCTCGTAACTCCAG

ACAAGCTAGCAGCGTGGCTCAAAGAGGATATCCTCTTACGACGCGTTGCC

CCGCCGCAAAAGAAACCACGGGCACGGGGTAAGGGCAAGGGCAAAGGAAA

GGCTGTAAAGTTACGGCGGCAGCTCGAACAGGAGCAGCTCGAGGCCGCGG

CCCTGGCAGAAGCCGAAAGCCTAGCCGAAGCTCTAGAGGTCCCCCTGGCC

GAGGCCGCCGAGCTGCTTGCGGACGACCGCGAGGGCTACGTGCCACCCAC

GGCCCTTGCGCCGGCTGCAGCAGACATTGCCGAAGGATCTCTGCTTACGA

GGGGCACTATCGACGCCTATATCGCGGCCGTTATCGAGCTCTGGCGGCTC

CAGGTAGCCCACGGCAACGCAAACACGGAAAACCCCCGGGGCGCCGCCGT

ACGAGGCTTCCTTGAGCAACGCGGCCGGCAGCGGGGGAAGCACGACCGCG

CCTCCTTTAAAGACCGCGGGACTGACGGGATCCAGGCCGGCTATTCACCC

GACGAATGGCTCCGGGTCCAGGATCTCCTTCTCAGCGGGGCCGCATACAT

GCCCCAAAACCTCCGTACGCGAGTTGACCTTCTATTCGGCCACTACTACC

TCTTACGGGGGGAAAACCGCCGTAAGATGGAGCTTGCAGACCTGTCTCTA

CTCGACTATCCGTCTTCAGAAGGCCCGACCCCCTGTTGCTGCCTCGTTAC

CCTTTTGCGAGACGGTAAGCTAAACAAGACGACAAAGAAAGAGTTCATGG

GTGCCCTCCGGCATAAGGACCCGTTGTTCTGTACGCAGGGGGCCTTAGCA

CAGCTCTTCTTCTGGCGCTGGCACGTCGCCGGCGAGCCGTCCCCGTCCTT

CCGGCGCCGCCAGGACTGGTATCGGATCAAGGTTCTTGTCGGACGGGACC

GCGAGCAGGAGCTCTCGTACCCGACGCAGCTACAAGAGACCTGGCGTATC

TTCGGTGCTGCTGGCCTTATGGCGTCAAAGAAGACGCACCTCCCGCGCAG

GGTAGGCGCCCAGGACGCGGAGACCCACGGCACATCGCTCGCCCAGATCT

TGCAGGCCGGCCGCTGGAACCAGAACGTGCTCTGCCAGGCCTATCTTACG

CGTCTACCGCGCCAGTTCATGCGTATTGTTGCCGGCTTCTCGGCGTCACC

CGGGGACTACTTCCTCGCGCGTGCGGCCCACGAGCCCCCGTACGTCTTAC

AAAAGCAGCTCTGGCCGTGGATCGAGGAGTGGGAGCCTCGCTTTGAGGCT

CGCGCGCGCCGGCAATGCTGGGCAGAAGGTGGCCTCGACGACGACGACCT

AGCCGCCGACGGCTTCCTTAAGCTTATGCGGCGCCTGCGCATAGTACTGT

TACAGGACCTGGCTGTATTGCAGCCTCGCTATCCGTCGCTACCCTTCTTC

GCCTACGCCCCTTTTAACGGGCCCGAATGGGATGAGTTCGCCGTCGCCGT

TCGCTCTGACGCGGTAGGGGCTACGGGGCCGTTAAGCCTGCTCGTACAAC

GCGCGCTGCCAGAGCTTAGCGGTGTGTTAGAGAGCACACGCGAGGCCGTC

TTACAGAATAGCCAGCGGCTGGCCATCCGGCTAGAGGCCCGGCTAGAAGG

AATTCAGGACGGCCTCGATGCCCTCCTCCAAGGCAAGGTCCCTGTCACCT

TTACCGGCCACTTTGGAGCCGGGCCAGCAGTGTCGCTGGCGCCGGCGCCG

GCGCCGTCGACAGCCCCTACCTTGAACTTCAATACGGCTCCGGCTCCGGC

TCCAGAGCCTCCAGTACCTGGTATGCCCGTCGTTGCAGCCCTGGCCAAGG

TCTTTACAGTGCGGGATGTCTGGAAGGAATGGGAGGAAGGGATTGCAGGT

CAGCCGGCCGTACGGGTGCTAGAAGAGACGTGGGGAAGCCGCTGGCGCCC

GGGGAACGGGATCAGGGTGCAGTTCTGTCGCCGGAAGGTGATCTGGGATG

AGCTCTTGGCCCGCACGGCGTCCGGCAAGAACGAGGAGGAGGCAGTTGCA

GAGCTAGAGCTCTTACGCGCCGGCCGGAGCTTGAACCGGCTCGTCGATGA

GCTCAAACAGCGCCGCCGGCGGGGCCAGGGCCAGGGCAGGATACGGGTAC

AGGTAGGGACCCCCGTGCCCGATGACTCAGGCCCGGGACCAAGGCCGACT

CGGGGCCAGGGCCATCGGGGGCGCTGGGCTCGGCTAGGGAGGAGGCGGAC

CGCCCCTCGACGACGGTAAgctggctgtcaggtaggatctttatatattt

atagctataactagtccgtatctatctatctatctagaaagcgcattttt

ttttattgaatcttttttttttctttctttttttttgaatattaaggagc

agcaggatcagtgcattgctatatcgtagtccttgcagcgaagcctggcc

ccccagccgctgcacacgcaaatatctcagcgcccgctcgtctatacagc

atcgctcttgaaccaaacggaccgccagagcgtagcagtcctccagccct

gaaattgcatcgatttggctcctaaccgtcgagaaattcgtgtgcgcggc

gccgggcacttatcagatggcagttgtgtaatcacatacgatttcccgaa

attactccgcaaagcgaccccacgat

>Fot6

TACCATCCAGGACCACCTAATGCATAACCCCACCTAACGCATAACCAAAA

TTTCGCTCCTCCCATACAAAATCCAAATTTTCAATTGCATTGTATTCGGC

CAGGCAGGAAGATACTCGCGAACGAAGACCTACACTATATTGCTTTTTTC

ATCGAATTTTCATTTCGGCTTGAATTTTTCCCGTTCCGCCCCGTAGCCTG

GTTCTCTAGCCCTTTGAATTTTGTCGCATTTCACCTCATGTACCCTAGGC

GACTCCATCCACTCAAGCCGAACTTTCACCTAATTTACTTGCCACAACTC

CTTTCGTAATGGTTCACCGTTATTATACCGAAGATGACGTAGCTGAGGCT

ATATTCGACACCACCGATAGAGGCCTCTCACAGAATGAGGCGGCCCAGAA

ACGTGGAGTACCTCAGTGGACCATTTCGAGACGACTTTCCGGTCAAGCAA

GCAGGATTGAACGTATTCAGGCCCACCAGCGTATTACAAAGAGCCAGGAG

GAGACGCTTATTCGTTGGGTGCTGCGACAAGAGTCTCTAGGCTATGCTCC

CTCGCACAGCCAGGTCCGCGCCTGTGTCGAGGCCATCCTCCAATAACAAG

GCGATAACAAACCTTTAGGCAAGCACTGGACCACCAGGTTCGTCAAACGT

CACCCCAAGCTATCTACCAAGATTGGAAAACGTCAAGAAGCCGCCAGATT

TGACGGCTTTACTCCGAAGGCGGTCAATTGGTACTTCGATATCAGGGAGA

ACGAGTACGGCTGGATCAAGCCTGAGAACACAGTTAACGTGGACGAGGGC

GGTATAATGGCGGGTTTCGGTAAGTCTTCTGCAAATACTCTATAGGCTTT

TATAGGGCATAGCTAATTGGTTGTCCATGAGGTCTGGACAGCCTCGTGAT

CGGGAGCTCTGACCCGAAGAAGAAGGCGATGTTGAAAGGCGTTCAGTCGC

GCACCTGGACTTCGTTTATTGAGGCTGTCACCGCAACTGGCCGTGCTTTG

AAACCGGGAATTATCTTTAAAGGAAAAGAGCTGCAGAAGCAGTGGTTCCT

TAACGAATTCGAATTAATAGCGGACTGGCACTACATCACATCACCGAACG

GCTGGACAGACAACCATATTGCTCTCGAGTGGTTGAAGGACGTTTATCTA

CCACAAACGGAGCCTCGTGATGCATCGGATGCGAGACTTATTATCCTTGG

CGGTCACGGGAGCCATGCGCAGGTGAGCATTTCGGTGTTTTCGCCGCTGA

GTCACCCGCTTTAGGGTCGCTGACGAGCAGTCAGGACGAGTGGATGGCTA

CGTGCTTTCTGAATAACGTCTACTGCTGTTACTTACCAGCACATTGTTCC

CATGGTCTGCAGCCATTAGATAATGGGATTTTTAATGTCATCAAAGGTGC

GTACCGGAAAGAACTCCAAAAGCTGGCTAGTTTGACCGATTCTGCGCCGG

TTGACAAAGCCAACTTTATCCGGGCATATGCGAAAGCCAGGGAGGCGGGC

ATGAGGAAAGATGTTATCTTATCCGGCTGGAGGTTTACTGAAAATTGGCC

AATAAATCGGTATAAAGCTCTAGCACATCCTGAAATTCAACGGGACAAAG

AAAAACTGCTGGAAGAGTTAAAGACGCTGAGCCCTCCTCAACTGCACTCA

GATGATACGCCGAAAACGAGTCGGCAAGTAAAGGAAATGGCCAAACACCG

AAGCCGTCCGACCAGGCGGAAGTACAGTAAGATCGCAAAGGGATTGGAAG

CTTTAGAGATGAAGGTGGCCGTTCAGAATGCGCGGATTACTGGCCTCGAG

GAGCAGATGGCCCAGGTGCGGCGAGGGAAGAAAAGAAAGGCGGTACCAAA

CCCGAACCGACAATTCATGGCTCTGTCGGAGAATCTAGCGGCTGGAGAAG

CTCTCCCTGACTCAAAGGAGGCAGAAATAGAGGTGGATGTGGATGAGGAG

GTCGAGTCGGTGATTGAGGTGGGTGTCAGGTCAGAGGACGAACGTGATGA

CTTTATGGTCGTAGCAAAGCACTGCCAGCGCACACGGAGCGGTCGGGAGG

TCAAAAAGCCACGCAGACAGTAAAAATTGAGCAATTTTCTAGAAACTTTC

TCCAAAGCCTCATTTATTAGTTGAGAATCGGGCCTGTTTTTGTGGACATC

AAAATTTGGATTTTGTATGGGAGGGGCGAAATTTTGGTTATGCATTAGGT

GGGGTTATGCATTAGGTGGTCCTGGATGGTA

>Fot8

agtcgcccaccctagttatggcccacccctggctttggtccgccccacaa

acgcgctccgtcccacgcgtcagatcagcgctaccacctacgataaattg

tcaaaaatcgaatttttctctcgatacatacctttataatataaactcta

agggccaggctagttccagcgtccctcgaagggctcgtgctatcattttt

cgagcgcatgggaggacttgtgctccgggtgtttttggttcttagttggt

gttgctgacggagtgtatggttgtagaggaagaaggggaggatcgagagg

tcgaTTGAATGGTGCCCAACCTCAACGTACGGCACACGACGACCAACAGC

GGCTCACGGCCAATCAAGAAGAACATCTTAAGCAATGGATTTTGCGGCAG

GAGGCCCTCGGTTACGCGCCAACACACGCACAAGTGCGAGCAATCGCTAG

CAGCGTGCTTAAACAACAAGGTGACCACAAGCCCCTAGGGAGGAAGTGGT

CCAGTCACTTCGTGGAGCGCCATTTGGCCGTCAAGACAAAATTAGGCCGT

CGAACTGACTGGAAACGTATAAATGCGGCCACTCCGGATAATATCAGGCA

CCTGTTCAACCTATACGAGACTGTGAGCTGGATCCCTCCCCGGCGGAGGT

ACAACGCCGACGAGGGCGGCATTATGGAGGGCCAAGGCATTAACGGGCTT

GTTATCGGCTCCTCGCAGGAGAACCCTAACACGGTGCCAGTCAAAACGAT

AAATGCCCGGACTTGGACGTCTATTGTGGAGTGCATCTCGGCGCTCGGGG

TCGCTTTGAACCCCCTGGTCATCTTCAAGGCCAAAAGTATCCAAGAACAA

TGGTTCAAGAAGGATTTCCTAGCTAAGCATCCTGGCTGGCATGTTACCTT

TTCGGAAAACGGGTGGACAAGCAATGATATTGCTGTCGAGTGGCTAGGGA

AGGTGTTTTTACCCCAAACGCAACCCGAGGATCCAGCCGATGGTCGCCTA

CTCATCGTCGACGGTCACGGCAGTCATACCTCGGACGAATTTATGACTAT

GTGTTACCTAAACAACGTTCACTTACTCTTTTTCCCTGCTCATACTTCCC

ATGTCCTCCAGCCCTTGGATCTCGGCTGCTTTTCCAGTTTAAAAACGGCC

TATCGTAGGTTAATTGGGGAGCATACGGCTTTGACAGATGCGACAAAGGT

TGGAAAGGCAAACTTTCTCGAATTTTATGCGAAAGCTCGAGAAATTGGTC

TTCGAAAGGAAAACGTTCAATCTGGGTGGAAGGCGACAGGCTTATACCCT

AAAAGCGTAGCCAAACCTCTAAATTCTCGATGGGTCGTGGTGGCTAAACG

GCCAGCTATACCACTACGTGTCACCTCGGATATTTCAACTCCAAAACGTG

GCGGCGACGTCGTAAAGCTATTTGCCGAGAAGAGCGGCTCTCCCACTTCA

CGACTGTCTATTCGAAAAGCCGCTGCGGCGTTGGATAAGGTTGCGATGGA

GGTTATACTAAGGGACCGTGAGATCGAACGCTTGCGGGTGCAACTCGCCC

AGGCAAAACCGACTAAACGTCGTAAGATCGTCCAAGATCCGAACGAACGC

TTTGCAAGCCTTGCGCAGATACTTGCGCAAGCTAATCAAGAGCCTCAACA

ACGTGTTCGGAAGGCAAGAAACGCGGTACAAGAGGTCATTATCGTGGAAG

GGGAGAGTAGCTCTGAGTCGGAAGAGGAGCCGGCACTGGCTCGACGGTCG

GCACGCAATCGGCGACCAACAAAATGCTATATGGAGCGAGATTCATGTGC

AGACGAAGAAAGCGACTAAagagcgccaaatgcgattttccttgcatttt

taccatttaatttgactttgttgcgacctagttacgtcgatataccagtt

gtgcgatgtaggctaaatagagcgggccaaagccaggggtgggccaaaac

tagggtgggcgact
